# Diastereoselective synthesis of dispirooxindoles *via* [3+2] cycloaddition of azomethine ylides to 3-phenacylideneoxindoles and evaluation of their cytotoxicity†

**DOI:** 10.1039/c8ra04375b

**Published:** 2018-07-02

**Authors:** Ying Huang, Yi-Xin Huang, Jing Sun, Chao-Guo Yan

**Affiliations:** College of Chemistry & Chemical Engineering, Yangzhou University Yangzhou 225002 China cgyan@yzu.edu.cn; College of Medicine, Yangzhou University Yangzhou 225001 China

## Abstract

The three-component reaction of 1,2,3,4-tetrahydroisoquinoline, isatins and 3-phenacylideneoxindoles in refluxing ethanol afforded dispiro[indoline-3,1′-pyrrolo[2,1-*a*]isoquinoline-3′,3′-indolines] (4a–4x) in good yields *via* 1,3-dipolar cycloaddition of *in situ* generated azomethine ylide with the exocyclic double bond of 3-phenacylideneoxindoles. ^1^H NMR spectra and single crystal structures indicated the reaction has high regioselectivity and diastereoselectivity. Furthermore, their biological activities have been preliminarily demonstrated by *in vitro* evaluation against mouse breast cancer cells 4T1 and human liver cancer cells HepG2 by MTT assay. The results demonstrated that some of the compounds showed cytotoxicities to cell lines of 4T1 and HepG2, and indicated that novel spirooxindoles may become potential lead compounds for further biological screenings of their medicinal applications.

## Abstract 2

The three-component reaction of 1,2,3,4-tetrahydroisoquinoline, isatins and 3-phenacylideneoxindoles in refluxing ethanol afforded dispiro[indoline-3,1′-pyrrolo[2,1-*a*]isoquinoline-3′,3′-indolines] (4a–4x) in good yields *via* 1,3-dipolar cycloaddition.

## Graphical abstract

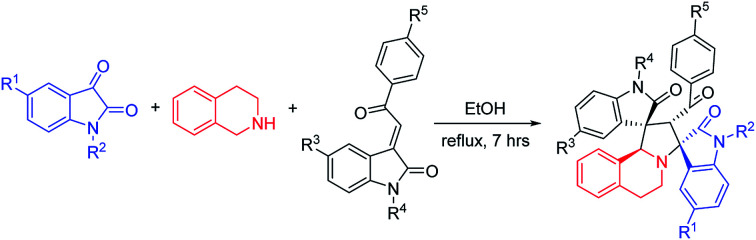

## 1. Introduction

**Fig. 1:**
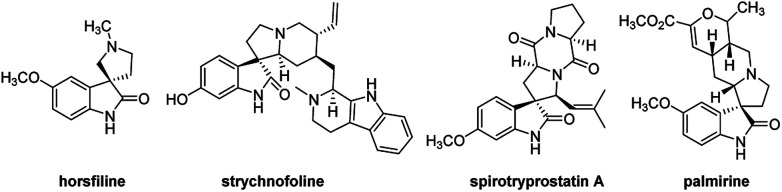
Representative spiropyrrolidinyl oxindole alkaloids.

The spirooxindole core is one of the most privileged heterocyclic rings, which not only exists in a number of naturally occurring substances, but also is featured in many medicinally relevant compounds with wide biological applications.^1,2^ Among various carbocyclic and heterocyclic spirooxindole systems, spiropyrrolidinyl oxindoles are frequently encountered in natural alkaloids and are often considered as attractive templates for drug discovery.^3^ Spiropyrrolidinyl oxindole is the spiro ring fusioning at the 3-position of the oxindole core with various substitutions around the pyrrolidine and oxindole rings. Some of them exhibit significant bioactivities such as horsfiline, strychnofoline, spirotryprostatin A and palmirine^4^ (Fig. 1). Apart from natural oxindole alkaloids, synthesized spiropyrrolidinyl oxindoles have been widely studied for their antiviral, antibacterial and anti-cancer activities.^5,6^ Their remarkable pharmacological activity and unique molecular architecture have made spiropyrrolidinyl oxindoles and their derivatives attractive synthetic targets. As a consequence, many efficient synthetic procedures have been developed for the preparation of the diversely structural spiropyrrolidinyl oxindoles.^7^

1,3-Dipolar cycloaddition reaction is an efficient and high-yielding, regio- and stereo controlled method for the synthesis of heterocyclic compounds. For the preparation of five-membered nitrogen-containing cyclic compounds, in particular pyrrolidines, dihydropyrroles, and pyrroles, [3+2] cycloaddition of azomethine ylides with alkenes is very effective and has been studied widely.^8,9^ If the azomethine ylides are generated from isatin derived compounds and α-amino acids through the thermal decarboxylation, pyrrolidine-containing spirooxindoles with high regioselectivity and stereoselectivity will be obtained.^10,11^

Heteroaromatic N-ylides such as pyridinium, thiazolium, quinolinium, isoquinolinium methylides which are readily available from the alkylation of azaaromatic heterocycles and sequential deprotonation reaction have also been used as one kind of reactive azomethine ylides extensively in cycloadditions for the synthesis of the fused heterocycles with a nitrogen at the point of fusion.^12^ Our group had reported the green synthetic methods for complex heterocyclic compounds, such as efficient synthesis of spiro[indoline-3,1′-pyrrolo[2,1-*a*]isoquinolines *via* 1,3-cycloaddition reactions of 3-phenacylideneoxindoles with aza-aromatic N-ylides generated from isoquinolinium salts.^13^ Wang's group synthesized some analogues by using 3,4-dihydroisoquinolinium salts.^14^ In addition, the 1,3-dipolar cycloaddition reactions of isatin, benzylamine and chalcone derivatives or benzylideneacetones are reported, in which azomethine ylides are *in situ* generated from isatin and benzylamine.^15^ Using 1,2,3,4-tetrahydroisoquinoline instead of benzylamine, the spiro compounds with key structure of spiro[indoline-3,1′-pyrrolo[2,1-*a*]isoquinolines could be obtained.^16^ In this paper, we wish to report an efficient 1,3-dipolar cycloaddition reaction of isatins, 1,2,3,4-tetrahydroisoquinoline and 3-phenacylideneoxindoles for regioselective and diastereoselective synthesis of novel functionalized spirooxindoles. Additionally, their biological activities have been preliminarily demonstrated by *in vitro* evaluation against mouse breast cancer cells 4T1 and human liver cancer cells HepG2 by MTT assay.

## 2. Results and discussions

### 2.1. Synthesis of dispirooxindole derivatives

**Scheme 1:**
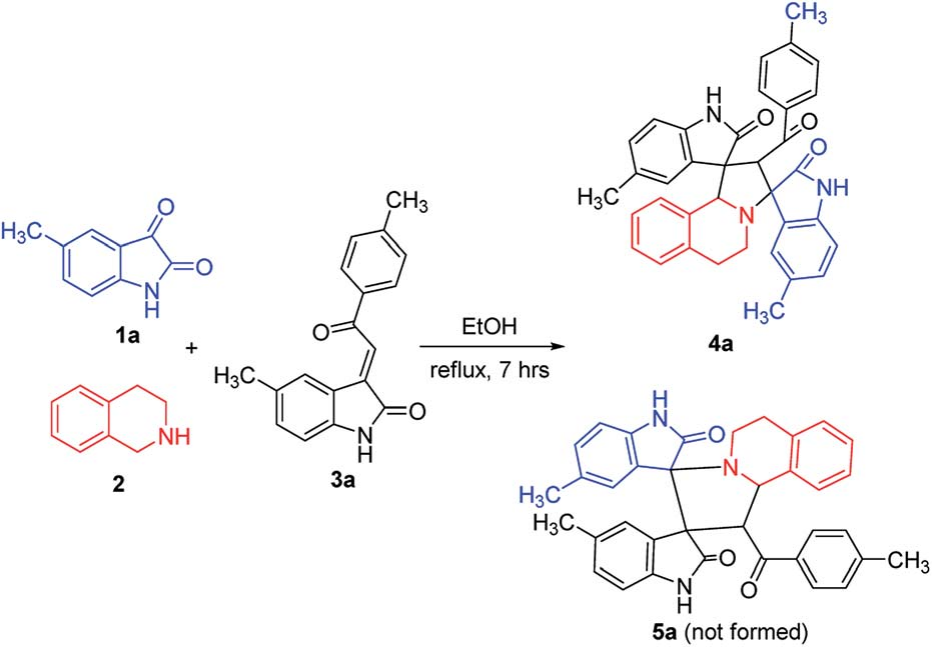
Three-component reaction for dispirooxindole 4a.

According to previously established reaction conditions for the domino reactions for the efficient synthesis of the functionalized spiro[indoline-3,1′-pyrrolo[2,1-*a*]isoquinolines,^13^ we have started the reaction of 5-methylisatin (1a), 1,2,3,4-tetrahydroisoquinoline (2) and 5-methyl-3-*p*-methylphenacylideneoxindole (3a) in a one-pot, three-component procedure depicted in Scheme 1. After refluxing the mixture of substrates in ethanol for about seven hours, it was pleased to find that the resulting precipitates were collected by filtration and characterized as the expected dispiro[indoline-3,1′-pyrrolo[2,1-*a*]isoquinoline-3′,3′-indoline] (4a) in 70% yield, in which the two scaffold of oxindolines exist on the 1,3-positions in the newly formed pyrrolidinyl ring. It should be pointed out that the isomeric product 5a, in which the two scaffold of oxindolines were connected together, was not formed in the reaction. This results also indicated this three-component reaction has high regioselectivity. Therefore, the expected three-component reaction successfully proceeded in very simple reaction conditions without adding other catalyst. When other solvent such as methanol, acetonitrile and toluene was used, the yield of the product (4a) was decreased.

**Fig. 2:**
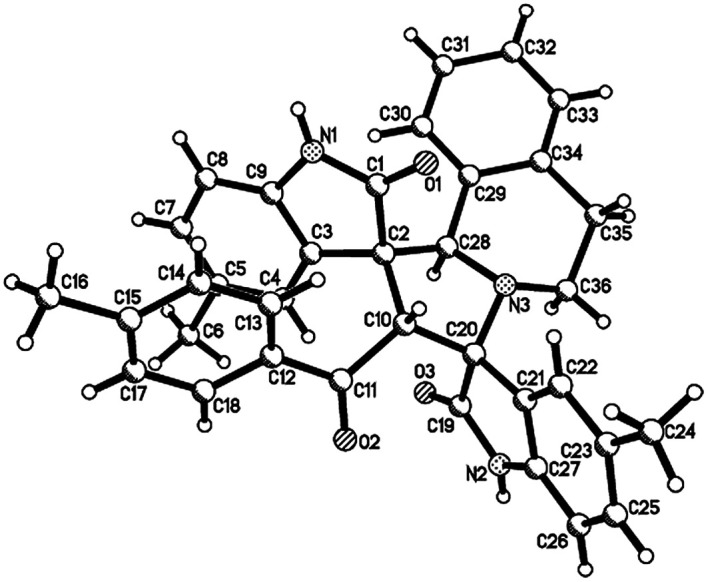
Single crystal structure of compound 4a.

**Fig. 3:**
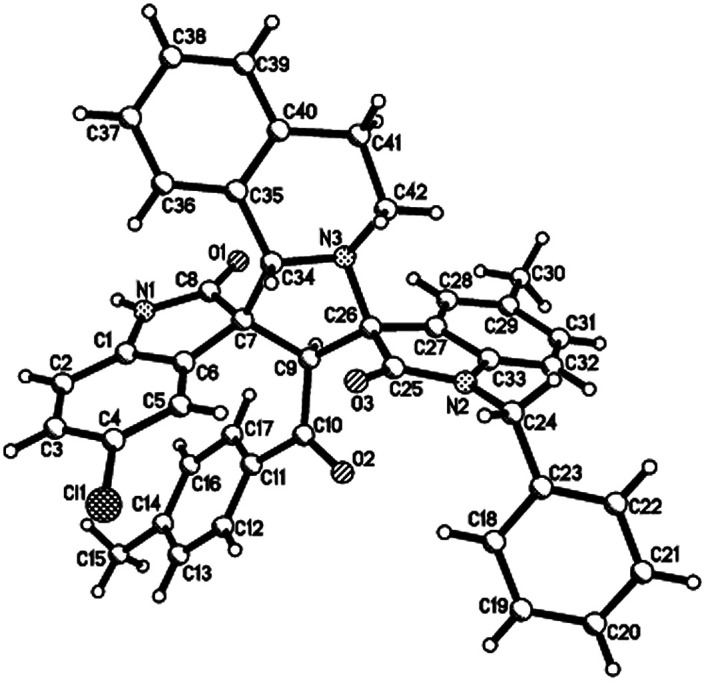
Single crystal structure of compound 4u.

**Fig. 4:**
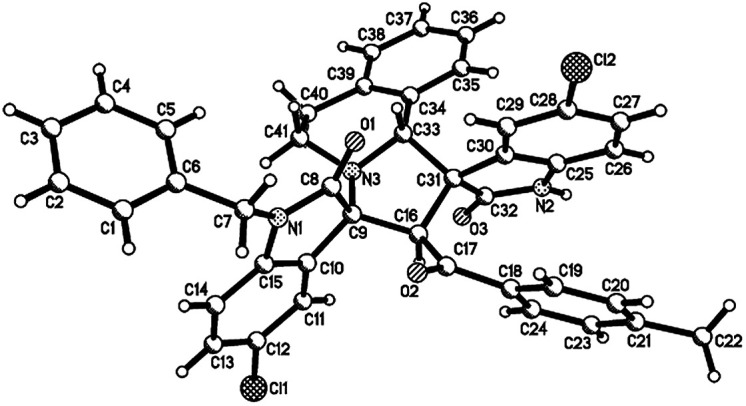
Single crystal structure of compound 4v.

Then, we extended the scope to the reaction under this simple reaction conditions. Various isatins (1a–f) and 3-phenacylideneoxindoles (3a–l) with both electron-withdrawing and electron releasing-substituents were employed in the three-component reaction under same conditions. The corresponding novel dispirooxindoles (4b–x) were successfully synthesized in high yields. The substituents on the both oxindoles showed little effect on the yields of the products. The results are summarized in Table 1. It should be pointed out that the pure products were usually obtained by filtration of the formed precipitates. The products were fully characterized by the spectroscopic methods. For examples, the IR spectrum of 4a showed three peaks of the carbonyl groups at 1725, 1676, 1626 cm^−1^. Because there are four chiral carbon atoms in the newly formed ring of pyrrolidine, several diastereoisomers might be formed in the 1,3-dipolar cycloaddition reaction. In the ^1^H NMR spectrum of 4a, two singlets appeared at *δ* 5.59 and *δ* 4.89 ppm were the signs of two cyclic CH unit in newly-formed pyrrolidine, and the two characteristic signals appeared at *δ* 10.38 and *δ* 10.34 ppm were the corresponding signs of NH protons in two oxindole rings. This result clearly showed that only one diastereoisomer existed in the obtained product. In ^13^C NMR spectrum of 4a, the two carbonyl group of the oxindole ring showed signs at *δ* 179.1 and 177.4 ppm, and the carbonyl group in benzoyl group showed sign at *δ* 196.1 ppm. The mass spectrum displayed a distinguished peak at *m*/*z* 554.2447 which further supported the formation of cycloadduct 4a. Other spiro compounds also displayed similar spectroscopy. But the ^1^H and ^13^C NMR of the compounds 4t and 4u clearly indicated two diastereoisomers existed in the obtained samples. The major/minor ratios of 4t/4t′ (90 : 10) and 4u/4u′ (75 : 25) were determined by integral of signs in the ^1^H NMR spectra. For determining the relative configuration of the spiro compounds 4a–4x, the single crystal structures of three compounds 4a, 4u and 4v (Fig. 2, 3 and 4) were successfully determined by X-ray diffraction. From the figures, it is clearly seen that the three single crystal structures have same relative configuration, in which two oxindole units existed at *trans*-position. The two protons in the ring of pyrrolidine also existed in *trans*-configuration. On the basis of NMR spectra and single crystal structures, we can concluded that this three-component reaction predominately give this kind of the diastereoisomer as major product and the other diastereoisomers were formed as minor product in few cases.

**Table 1:** Three-component reaction for synthesis of spirooxindoles 4a–x^a^

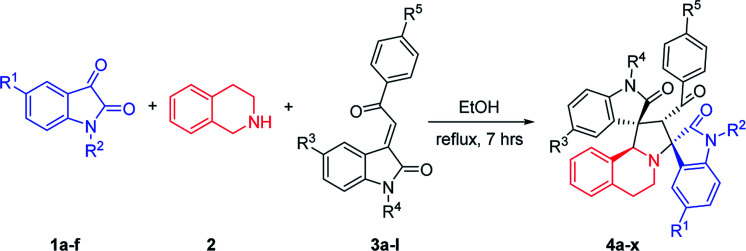							
Entry	Compd	R^1^	R^2^	R^3^	R^4^	R^5^	Yield^b^ (%)
1	4a	CH_3_	H	CH_3_	H	CH_3_	70
2	4b	CH_3_	H	Cl	H	OCH_3_	76
3	4c	Cl	H	CH_3_	H	CH_3_	81
4	4d	CH_3_	H	H	H	H	90
5	4e	CH_3_	H	CH_3_	H	Cl	80
6	4f	CH_3_	H	Cl	H	CH_3_	50
7	4g	Cl	H	CH_3_	H	Cl	82
8	4h	Cl	H	Cl	H	OCH_3_	67
9	4i	Cl	H	F	H	CH_3_	57
10	4j	CH_3_	H	Cl	CH_2_Ph	Cl	57
11	4k	CH_3_	H	H	CH_2_Ph	H	58
12	4l	CH_3_	H	CH_3_	CH_2_Ph	CH_3_	62
13	4m	CH_3_	H	CH_3_	CH_2_Ph	Cl	66
14	4n	CH_3_	H	CH_3_	C_4_H_9_	CH_3_	90
15	4o	Cl	H	Cl	C_4_H_9_	CH_3_	72
16	4p	H	CH_3_	CH_3_	H	CH_3_	85
17	4q	H	CH_3_	Cl	H	CH_3_	63
18	4r	H	CH_3_	CH_3_	CH_2_Ph	Cl	58
19	4s	CH_3_	CH_2_Ph	CH_3_	H	CH_3_	79
20	4t	CH_3_	CH_2_Ph	Cl	H	OCH_3_	80^c^
21	4u	CH_3_	CH_2_Ph	Cl	H	CH_3_	62^d^
22	4v	Cl	CH_2_Ph	Cl	H	CH_3_	76
23	4w	CH_3_	CH_2_Ph	CH_3_	CH_2_Ph	Cl	60
24	4x	Cl	C_4_H_9_	CH_3_	H	CH_3_	50

a Reaction conditions: isatin (0.30 mmol), tetrahydroisoquinoline (0.30 mmol), 3-phenacylideneoxindole (0.25 mmol); reflux, 7 h.

b Isolated yields.

c Ratio of 4t : 4′t = 90 : 10.

d Ratio of 4u : 4′u = 75 : 25.

### 2.2. Viability of two cancer cells of compounds 4a–4x

All newly synthesized compounds were subjected to a preliminary evaluation on their *in vitro* cytotoxic activity, which was represented as the inhibition rate of the tested compounds to mouse breast cancer cells 4T1 and human liver cancer cells HepG2 at the concentration of 200 μg mL^−1^. The cytotoxic activity to 4T1 cells was not good as we expected. Among the tested compounds 4, compound 4i (R^3^ = F) displayed highest cytotoxic activity to HepG2 cells with the inhibition rate of 73.50%, followed by 4h (R^3^ = Cl) and 4d (R^3^ = H) (Table 2). Then the inhibition rate of human liver cancer cells line HepG2 of these three compounds were tested at three concentrations of 50, 100 and 200 μg mL^−1^ (Table 3).

**Table 2:** Preliminary evaluation on the anticancer activities of synthesized compounds 4a–x on 4T1 and HepG2 cancer cell lines (200 μg mL^−1^)^a^

Compound	4T1 cell death				HepG2 cell death			
	24 h		48 h		24 h		48 h	
	%	Mean ± S.D.	%	Mean ± S.D.	%	Mean ± S.D.	%	Mean ± S.D.
4a	2.31	0.915 ± 0.011	25.80	0.870 ± 0.015	2.84	0.868 ± 0.024	32.86	0.506 ± 0.004
4b	2.23	0.916 ± 0.016	15.05	0.984 ± 0.014	3.99	0.858 ± 0.023	19.34	0.591 ± 0.110
4c	6.99	0.871 ± 0.015	20.36	0.928 ± 0.010	5.67	0.843 ± 0.016	19.71	0.589 ± 0.060
4d	6.35	0.877 ± 0.040	20.82	0.923 ± 0.014	7.16	0.830 ± 0.016	**66.72**	0.145 ± 0.046
4e	7.88	0.863 ± 0.022	20.64	0.925 ± 0.060	2.97	0.867 ± 0.014	13.27	0.629 ± 0.058
4f	6.45	0.876 ± 0.012	19.43	0.937 ± 0.098	4.59	0.853 ± 0.013	14.22	0.623 ± 0.045
4g	/	0.940 ± 0.061	27.64	0.851 ± 0.034	3.71	0.861 ± 0.012	23.16	0.567 ± 0.056
4h	5.23	0.887 ± 0.045	23.25	0.896 ± 0.137	6.12	0.839 ± 0.013	**70.11**	0.098 ± 0.006
4i	10.38	0.839 ± 0.025	33.31	0.791 ± 0.068	5.34	0.846 ± 0.028	**73.50**	0.088 ± 0.004
4j	3.42	0.904 ± 0.020	17.87	0.954 ± 0.051	5.02	0.849 ± 0.019	25.25	0.554 ± 0.066
4k	3.43	1.329 ± 0.112	10.88	1.237 ± 0.033	/	1.193 ± 0.024	2.36	1.211 ± 0.106
4l	/	1.423 ± 0.013	10.54	1.241 ± 0.017	/	1.208 ± 0.012	/	1.563 ± 0.177
4m	8.16	1.268 ± 0.032	20.71	1.099 ± 0.084	/	1.184 ± 0.003	4.22	1.188 ± 0.012
4n	4.28	1.318 ± 0.018	15.68	1.169 ± 0.057	0.66	1.170 ± 0.019	11.82	1.094 ± 0.026
4o	0.23	1.370 ± 0.021	12.69	1.211 ± 0.020	/	1.208 ± 0.030	/	1.441 ± 0.229
4p	/	1.377 ± 0.011	22.86	1.070 ± 0.040	/	1.180 ± 0.028	/	1.393 ± 0.424
4q	/	1.514 ± 0.009	4.65	1.322 ± 0.018	23.9	0.897 ± 0.022	9.88	1.118 ± 0.137
4r	/	1.402 ± 0.027	5.11	1.316 ± 0.021	/	1.217 ± 0.018	/	1.285 ± 0.113
4s	/	1.376 ± 0.016	8.20	1.273 ± 0.007	/	1.292 ± 0.025	7.23	1.151 ± 0.048
4t	/	1.481 ± 0.016	/	1.465 ± 0.028	/	1.385 ± 0.020	10.09	1.115 ± 0.069
4u	2.75	1.108 ± 0.039	/	1.131 ± 0.001	2.75	1.117 ± 0.154		1.053 ± 0.019
4v	/	1.125 ± 0.065	/	1.211 ± 0.021	/	1.172 ± 0.018	/	1.036 ± 0.010
4w	2.40	1.187 ± 0.060	5.249	1.197 ± 0.005	2.40	1.121 ± 0.038	5.25	0.947 ± 0.026
4x	/	1.185 ± 0.033	/	1.201 ± 0.002	/	1.194 ± 0.042	/	1.314 ± 0.110

a /: no activity.

**Table 3:** Preliminary evaluation on the anticancer activities of 4d, 4h and 4i on HepG2 cancer cell lines

Compound	200 μg mL^−1^		100 μg mL^−1^		50 μg mL^−1^	
	Cell death		Cell death		Cell death	
	%	Mean ± S.D.	%	Mean ± S.D.	%	Mean ± S.D.
4d	67.40	0.325 ± 0.018	30.93	0.691 ± 0.062	16.12	0.838 ± 0.040
4h	59.74	0.402 ± 0.014	30.64	0.693 ± 0.028	20.57	0.794 ± 0.028
4i	79.30	0.209 ± 0.040	70.40	0.474 ± 0.056	44.08	0.559 ± 0.003

In detail, compounds 4a–4i which have no substituents on N atom of isatin (R^2^ = H) and 3-phenacylideneoxindole (R^4^ = H) inhibited the growth of HepG2 cells at the concentration of 200 μg mL^−1^, displayed promising cytotoxicity to HepG2 cells with inhibition rates varying from 13.27% to 73.50%. These results were much better than the other compounds (4j–4x) which have N-substituents such as CH_3_, C_4_H_9_ and CH_2_Ph. Compounds 4j–4x tend to precipitate out during the dilution process. These results would suggest that the polarity or lipo-hydro partition coefficient (log *P*) of the compound has a significant effect on its activity (Table 2).

**Fig. 5:**
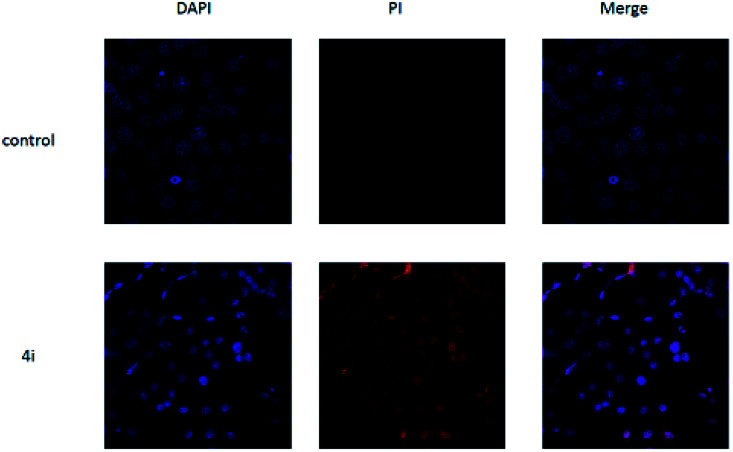
The morphological features of survival status were monitored by fluorescence microscopy after staining with DAPI.

**Fig. 6:**
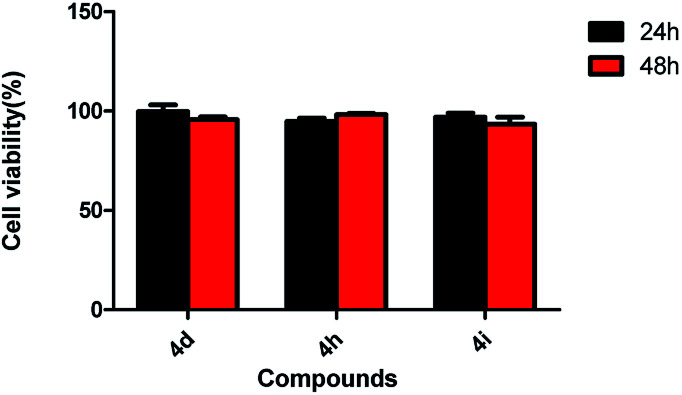
Effect of compound 4d, 4h and 4i on 3T3 cell viabilities (200 μg mL^−1^).

As shown in Fig. 5, untreated cells exhibit regular blue colour. In contrast, cells treated with 4i at the concentration of 200 μg mL^−1^ showed clear red colour. It indicated that large amount of HepG2 cells died after treatment with 4i. Compounds 4d, 4h and 4i showed a tendency of concentration-dependent cytotoxicity because their inhibition rates were greatly enhanced with increasing concentration. Among them, compound 4i (R^3^ = F) was the most powerful to inhibit the growth of HepG2 cells to 44.08% at the concentration of 50 μg mL^−1^ (Table 3). The cytotoxicity of compounds 4d, 4h and 4i was tested on 3T3 cells at the concentration of 200 μg mL^−1^, the results showed that their cytocompatibility were good, which meant that they have cytotoxicity to cancer cells and were not toxic to normal cells (Fig. 6).

## 3. Conclusions

In summary, we have developed an efficient strategy for the synthesis of complex spiro compounds by a 1,3-dipolar cycloaddition of 3-methyleneoxindoles with 1,2,3,4-tetrahydroisoquinoline and isatin derivatives. This three-component reaction afforded the anticipated spirooxindoles in excellent yields with high regio- and stereoselectivity. This synthetic protocol has advantages of mild reaction conditions, easily accessible starting materials and easy purification of the products, which makes it a useful and attractive method for the synthesis of the complex spiro heterocycles in synthetic and medicinal chemistry. Furthermore, the bioactivity detection of these spirooxindoles has led to the discovery of three compounds with promising cytotoxicity to HepG2 cells. The preliminary bioassay of these compounds will enlighten further structural modification and bioactivity studies on spirooxindoles for their medicinal applications.

## 4. Experimental

### 4.1. Chemistry

All reactions were performed in open atmosphere unless stated. All reagents, unless otherwise indicated, were obtained from commercial sources. ^1^H and ^13^C NMR spectra were recorded on a Bruker AV-600 instrument or Variance 400 spectrometer with DMSO-*d*_6_ or CDCl_3_ as solvent and tetramethylsailane (TMS) was used as internal standard (*δ* in ppm). IR spectra were obtained on a Bruker Tensor 27 spectrometer (KBr disc). HRMS were recorded on a Bruker UHR-TOF maXis spectrometer. X-ray data were collected on a Bruker Smart APEX-2 diffractometer. Melting points were taken on a hot-plate microscope apparatus and were uncorrected.

### 4.2. General procedure for the preparation of 4a–4x

A mixture of isatin (0.3 mmol), 1,2,3,4-tetrahydroisoquinoline (0.3 mmol), and 3-phenacylideneoxindole (0.25 mmol) in ethanol (10 mL) was stirred at reflux for seven hours. After completion of the reaction, as indicated by TLC, the solid was separated by filtration, washed with cold ethanol and vacuum dried. In some cases, additional purification by TLC was necessary.

5,5′′-Dimethyl-2′-(4-methylbenzoyl)-6′,10*b*′-dihydro-2′*H*, 5′*H*-dispiro[indoline-3,1′-pyrrolo[2,1-*a*]isoquinoline-3′,3′-indoline]-2,2′′-dione (4a). White solid, 70%, mp. 244–245 °C; ^1^H NMR (400 MHz, DMSO-*d*_6_) *δ*: 10.38 (s, 1H, NH), 10.34 (s, 1H, NH), 7.63 (s, 1H, ArH), 7.23 (s, 1H, ArH), 7.10–7.00 (m, 7H, ArH), 6.88 (brs, 1H, ArH), 6.82–6.77 (m, 2H, ArH), 6.36 (d, *J* = 7.6 Hz, 1H, ArH), 6.24 (d, *J* = 7.2 Hz, 1H, ArH), 5.59 (s, 1H, CH), 4.89 (s, 1H, CH), 2.86–2.78 (m, 2H, CH), 2.64–2.60 (m, 2H, CH), 2.29 (s, 3H, CH_3_), 2.21 (s, 6H, CH_3_); ^13^C NMR (150 MHz, DMSO-*d*_6_) *δ*: 196.1, 179.1, 177.4, 142.7, 141.1, 139.1, 135.2, 134.5, 134.2, 130.4, 130.3, 130.2, 129.9, 128.7, 128.5, 127.2, 126.9, 126.3, 126.1, 125.4, 124.8, 123.1, 109.1, 108.6, 70.6, 68.7, 66.1, 58.2, 41.4,29.3, 21.0, 20.9, 20.6; IR (KBr) *ν*: 3355, 3170, 3029, 2916, 2832, 1725, 1676, 1626, 1607, 1574, 1494, 1428, 1373, 1341, 1296, 1248, 1202, 1166, 1042, 1008, 947, 905, 810, 756, 730 cm^−1^; MS (*m*/*z*): HRMS (ESI) calcd for C_36_H_32_N_3_O_3_ ([M + H]^+^): 554.2438, found: 554.2447.

### 4.3. Biology

Three cancer cell lines are obtained from experimental cell resource centre of Shanghai Institutes for Biological Sciences. The relative cell viability was recorded by a microplate reader (SYenergy 2). The cell death stain was imaged by laser scanning confocal microscopy (LSCM, TLS SP8 STED).

#### Cytotoxic evaluation of compounds 4a–4x to mouse breast cancer cells 4T1 and human liver cancer cells HepG2.

##### Cell viability assay

The cell viability was measured by MTT assay. Mouse breast cancer cells 4T1, Human liver cancer cells HepG2 and Mouse fibroblasts cells 3T3 were separately cultured in DMEM medium containing 10% fetal bovine serum and 1% penicillin–streptomycin at 37 °C and 5% CO_2_. For *in vitro* cytotoxicity assay, 4T1, HepG2 and 3T3 cells were seeded into 96-well plates at 8 × 10^3^/well until adherent and then incubated with various concentrations of 4a–4x for 24 and 48 h. Ten microliters of 5 mg mL^−1^ MTT solution was then added into each well, followed by incubation for 24 and 48 h at 37 °C in the presence of 5% CO_2_. At the end of the incubated time, 100 μL of DMSO was added to dissolve the formazan crystals. Finally, the absorbance at 490 nm of each well was recorded by a microplate reader (SYenergy 2). The final cell viabilities were calculated by [OD_(tested compounds group)_/OD_(control group)_] × 100%.

##### Cell death stain

To investigate the cell survival status, HepG2 cells (1 × 10^5^/well) suspended in 1 mL of DMEM medium were seeded in a 24-well plate with cell climbing slices on the bottom of the plate for 12 h, and then incubated with 4i (4h, 4d) (the final concentration was 200 μg mL^−1^) at 37 °C for 48 h. The cells were then rinsed twice with 0.01% PBS, fixed with 4% paraformaldehyde for 15 min, 0.4% Triton for 5 min and stained with DAPI and PI for 10 min. After rinsing with PBS three times, cell climbing slices were placed on the glass slides and imaged by laser scanning confocal microscopy (LSCM, TLS SP8 STED).

## Conflicts of interest

There are no conflicts to declare.

## Supplementary Material

RA-008-C8RA04375B-s001

RA-008-C8RA04375B-s002
