# Supplementary material for: Diastereoselective synthesis of dispirooxindoles via [3+2] cycloaddition of azomethine ylides to 3-phenacylideneoxindoles and evaluation of their cytotoxicity
Source: RSC Adv. 2018 Jul 2;8(42):23990–5. doi: 10.1039/c8ra04375b (PMC9081698; doi:10.1039/c8ra04375b)
Supplement: RA-008-C8RA04375B-s001 [file RA-008-C8RA04375B-s001.pdf]

Electronic Supplementary Material (ESI) for RSC Advances.
This journal is © The Royal Society of Chemistry 2018

Diastereoselective synthesis of dispirooxindoles via [3+2] cycloaddition of azomethine ylides to 3-phenacylideneoxindoles and evaluation on their cytotoxicity

Ying Huang, Yixin Huang, Jing Sun and Chao-Guo Yan*

Supporting Information

^{1}H NMR, ^{13}C NMR and HRMS spectra of all compounds 2-49.

Characterization data and spectra of the compounds:

5,5''-Dimethyl-2'-(4-methylbenzoyl)-6',10b'-dihydro-2'H,5'H-dispiro[indoline-3,1'-pyrrolo[2,1-a]isoquinoline-3',3'-indoline]-2,2''-dione (4a)

white solid, 70%, m.p. 244~245℃; ^{1}H NMR (400 MHz, DMSO-d_6) δ: 10.38 (s, 1H, NH), 10.34 (s, 1H, NH), 7.63 (s, 1H, ArH), 7.23 (s, 1H, ArH), 7.10~7.00 (m, 7H, ArH), 6.88 (brs, 1H, ArH), 6.82~6.77 (m, 2H, ArH), 6.36 (d, J = 7.6Hz, 1H, ArH), 6.24 (d, J = 7.2Hz, 1H, ArH), 5.59 (s, 1H, CH), 4.89 (s, 1H, CH), 2.86~2.78 (m, 2H, CH), 2.64~2.60 (m, 2H, CH), 2.29 (s, 3H, CH_3), 2.21 (s, 6H, CH_3); ^{13}C NMR (150 MHz, DMSO-d_6) δ: 196.1, 179.1, 177.4, 142.7, 141.1, 139.1, 135.2, 134.5, 134.2, 130.4, 130.3, 130.2, 129.9, 128.7, 128.5, 127.2, 126.9, 126.3, 126.1, 125.4, 124.8, 123.1, 109.1, 108.6, 70.6, 68.7, 66.1, 58.2, 41.4, 29.3, 21.0, 20.9, 20.6; IR(KBr) υ: 3355, 3170, 3029, 2916, 2832, 1725, 1676, 1626, 1607, 1574, 1494, 1428, 1373, 1341, 1296, 1248, 1202, 1166, 1042, 1008, 947, 905, 810, 756, 730cm^{-1}; MS (m/z): HRMS (ESI) Calcd. for $C_{36}H_{32}N_3O_3$ ($[M+H]^+$): 554.2438, found: 554.2447.

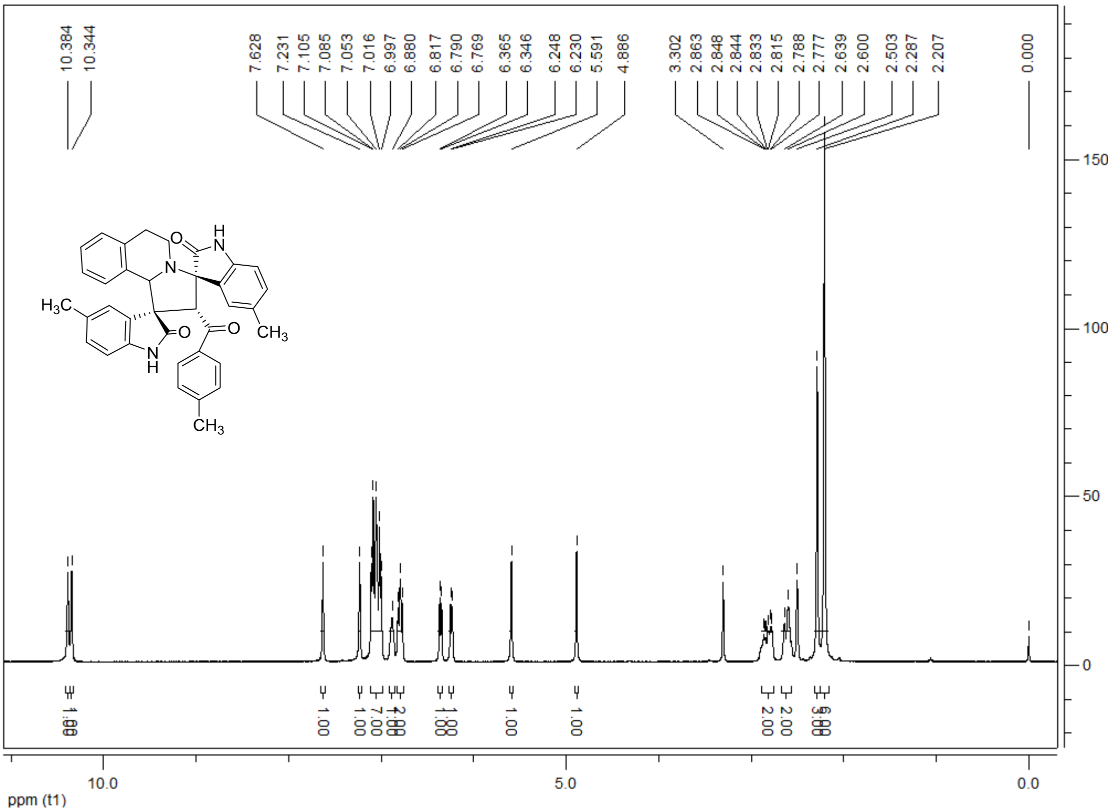

Copy of ^{1}H NMR spectrum of **4a** in DMSO-d_6

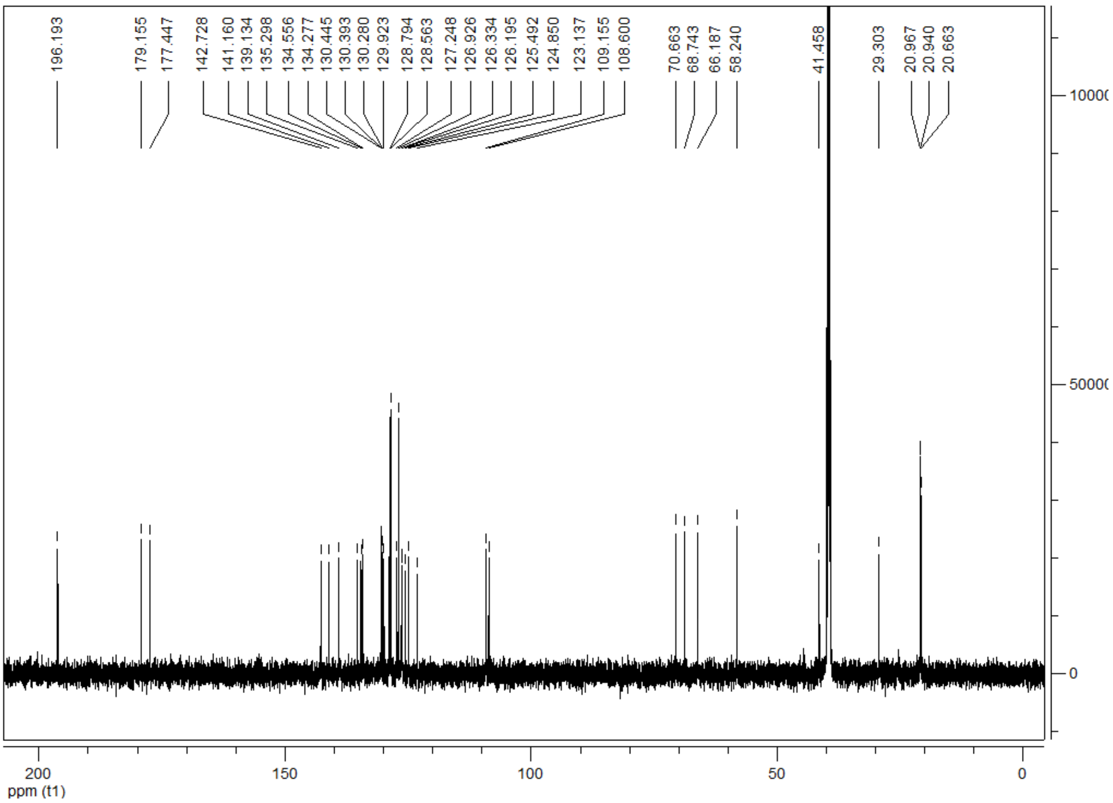

Copy of ^{13}C NMR spectrum of **4a** in DMSO-d_6

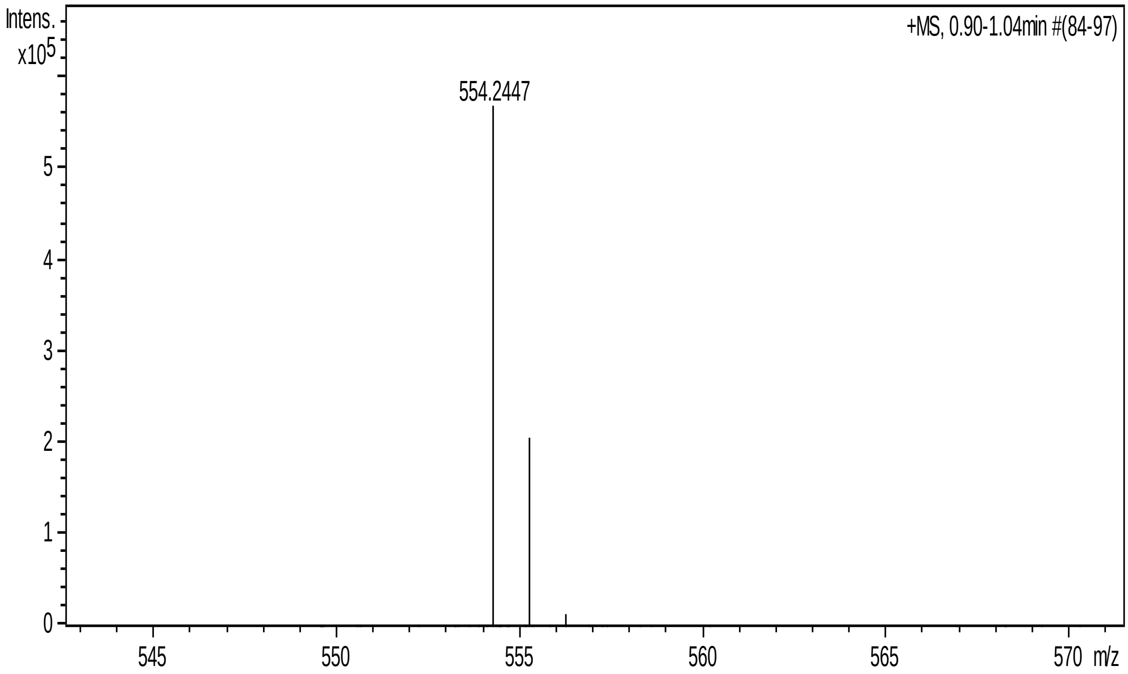

Copy of HRMS of **4a**

5-Chloro-2'-(4-methoxybenzoyl)-5''-methyl-6',10b'-dihydro-2'H,5'H-dispiro[indoline-3,1'-pyrrolo[2,1-a]isoquinoline-3',3''-indoline]-2,2''-dione (4b)

white solid, 76%, m.p. 248~249℃; ^{1}H NMR (600 MHz, DMSO-d_6) δ: 10.71 (s, 1H, NH), 10.46 (s, 1H, NH), 7.88 (s, 1H, ArH), 7.24~7.23 (m, 3H, ArH), 7.09~7.08 (m, 4H, ArH), 6.94~6.91 (m, 1H, ArH), 6.80~6.78 (m, 3H, ArH), 6.53 (d, J = 8.4Hz, 1H, ArH), 6.22 (d, J = 7.8Hz, 1H, ArH), 5.59 (s, 1H, CH), 4.89 (s, 1H, CH), 3.72 (s, 3H, OCH_3), 2.87~2.83 (m, 2H, CH), 2.65~2.60 (m, 2H, CH), 2.29 (s, 3H, CH_3); ^{13}C NMR (100 MHz, DMSO-d_6) δ:195.0, 179.2, 178.0, 163.2, 141.5, 140.9, 135.3, 135.0, 132.7, 130.9, 130.4, 129.8, 129.6, 129.3, 128.7, 127.2, 126.8, 126.3, 126.2, 126.1, 125.4, 123.4, 113.9, 110.8, 109.7, 71.3, 69.2, 66.3, 59.1, 55.9, 41.9, 29.6, 21.1; IR(KBr) υ: 3370, 3171, 3053, 2947, 2898, 2843, 1716, 1661, 1623, 1601, 1571, 1494, 1437, 1325, 1245, 1218, 1172, 1080, 1029, 943, 898, 845, 808, 751, 731cm^{-1}; MS (m/z): HRMS (ESI) Calcd. for $C_{35}H_{29}ClN_3O_4$ ($[M+H]^+$): 590.1841, found: 590.1835.

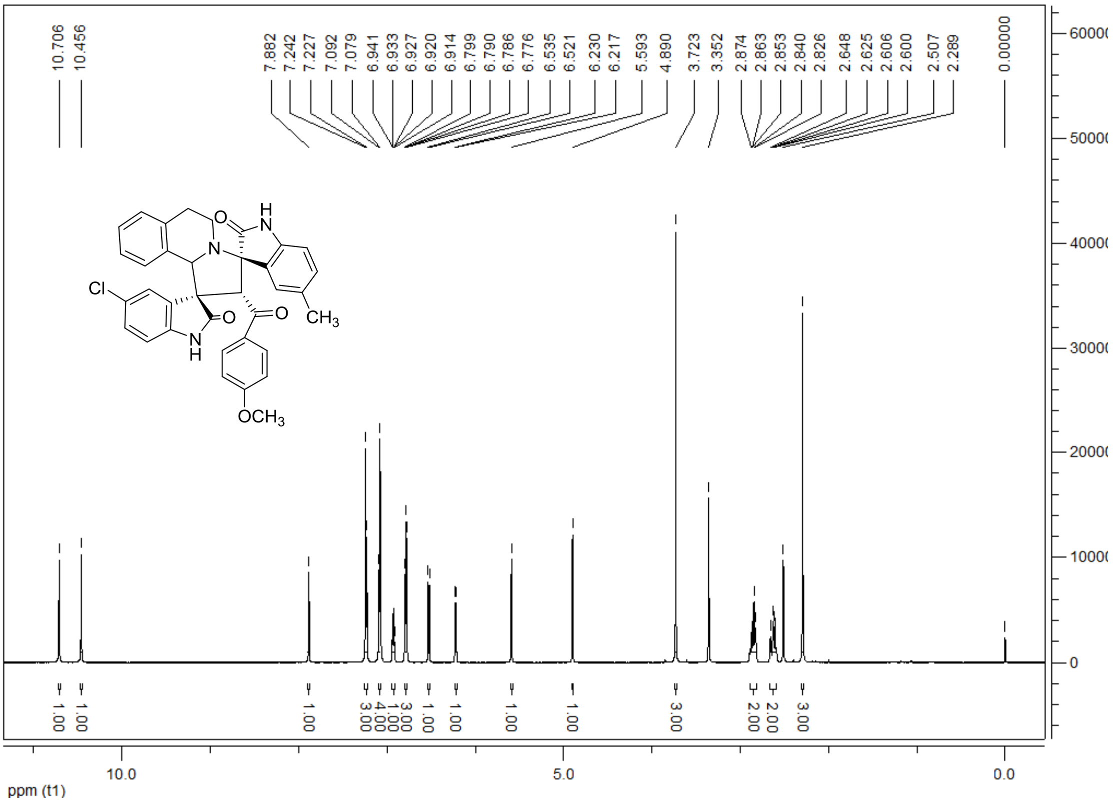

Copy of ^{1}H NMR spectrum of **4b** in DMSO-d_6

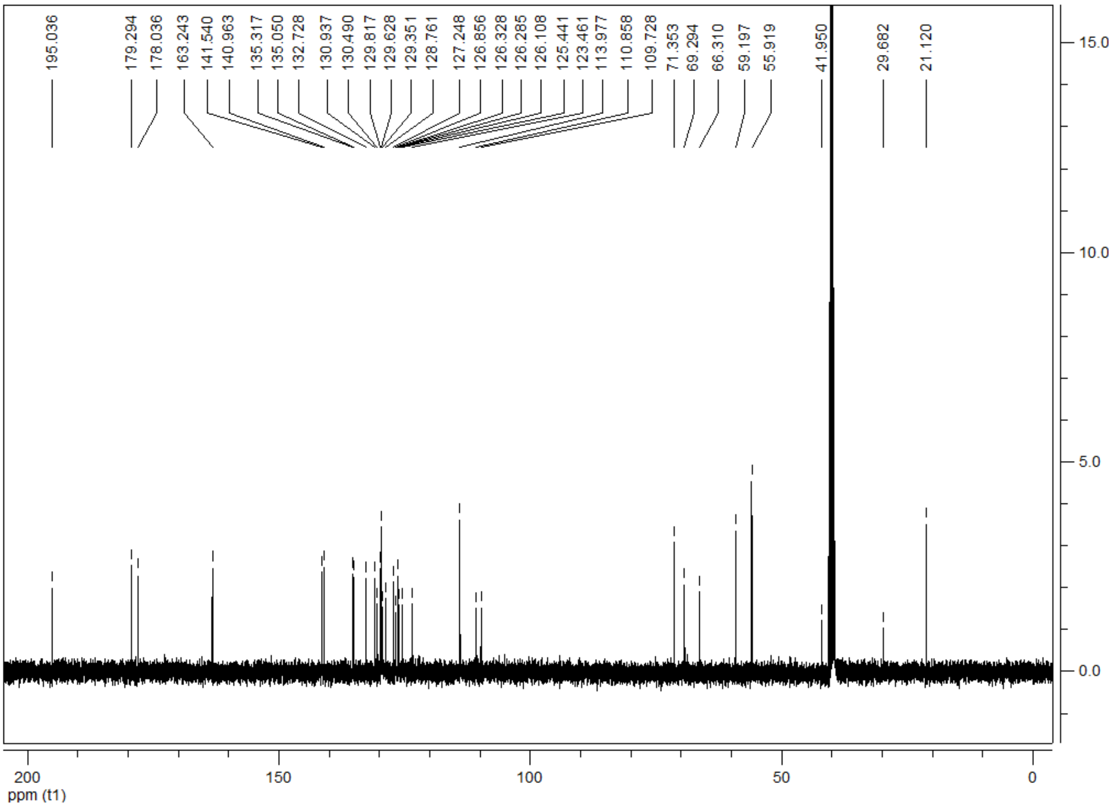

Copy of ^{13}C NMR spectrum of **4b** in DMSO-d_6

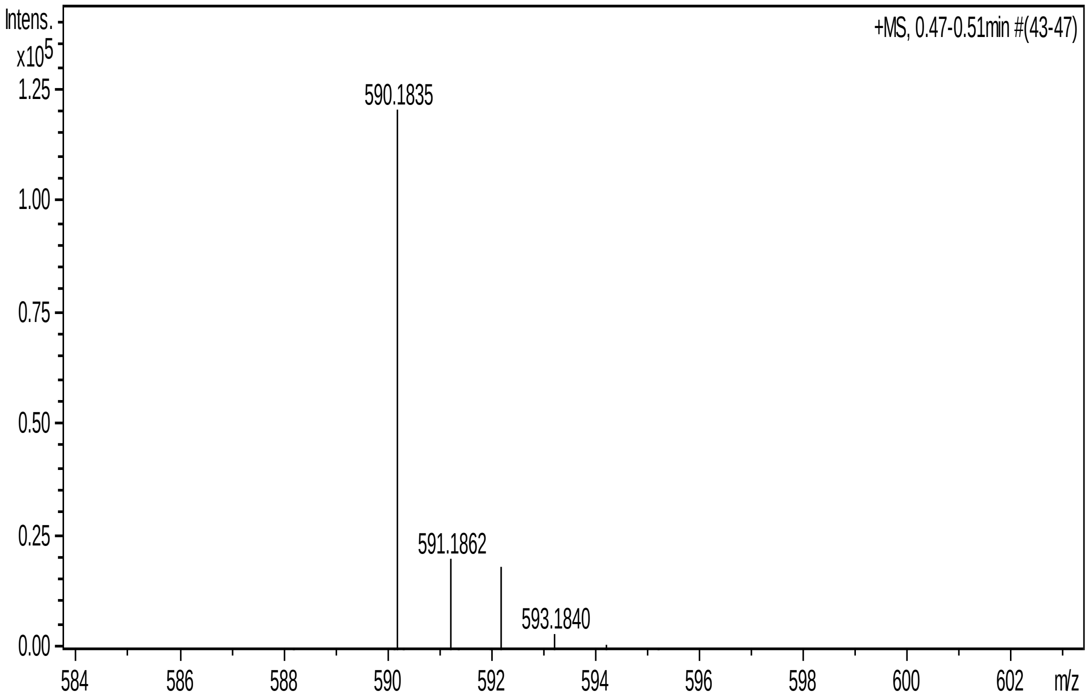

Copy of HRMS of **4b**

5''-Chloro-5-methyl-2'-(4-methylbenzoyl)-6',10b'-dihydro-2'H,5'H-dispiro[indoline-3,1'-pyrrolo[2,1-a]isoquinoline-3',3''-indoline]-2,2''-dione (4c)

white solid, 81%, m.p. 252~254℃; ^{1}H NMR (600 MHz, DMSO-d_6) δ: 10.65 (s, 1H, NH), 10.45 (s, 1H, NH), 7.57 (s, 1H, ArH), 7.41~7.40 (m, 1H, ArH), 7.34 (dd, J_1= 8.4Hz, J_2= 2.0Hz, 1H, ArH), 7.11 (d, J = 8.0Hz, 2H, ArH), 7.06~7.05 (m, 2H, ArH), 7.00 (d, J = 8.0Hz, 2H, ArH), 6.92 (d, J = 8.4Hz, 1H, ArH), 6.9~6.88 (m, 1H, ArH), 6.80 (d, J = 7.8Hz, 1H, ArH), 6.35 (d, J = 7.8Hz, 1H, ArH), 6.24 (d, J = 7.8Hz, 1H, ArH), 5.54 (s, 1H, CH), 4.90 (s, 1H, CH), 2.91~2.86 (m, 1H, CH), 2.77~2.74 (m, 1H, CH), 2.65~2.62 (m, 1H, CH), 2.60~2.56 (m, 1H, CH), 2.21 (s, 3H, CH_3), 2.20 (s, 3H, CH_3); ^{13}C NMR (100 MHz, DMSO-d_6) δ:196.7, 179.3, 177.5, 143.1, 142.9, 139.6, 135.5, 134.9, 134.6, 130.9, 130.5, 130.0, 129.7, 129.2, 129.1, 128.9, 127.4, 126.7, 126.5, 125.9, 125.9, 124.8, 123.5, 111.3, 109.1, 71.0, 69.0, 66.8, 58.3, 41.9, 29.7, 21.4, 21.3; IR(KBr) υ: 3360, 3173, 3031, 2946, 2909, 2837, 1714, 1667, 1607, 1482, 1433, 1338, 1290, 1245, 1183, 1043, 1007, 945, 885, 810, 753, 732cm^{-1}; MS (m/z): HRMS (ESI) Calcd. for $C_{35}H_{29}ClN_3O_3$ ($[M+H]^+$): 574.1892, found: 574.1877.

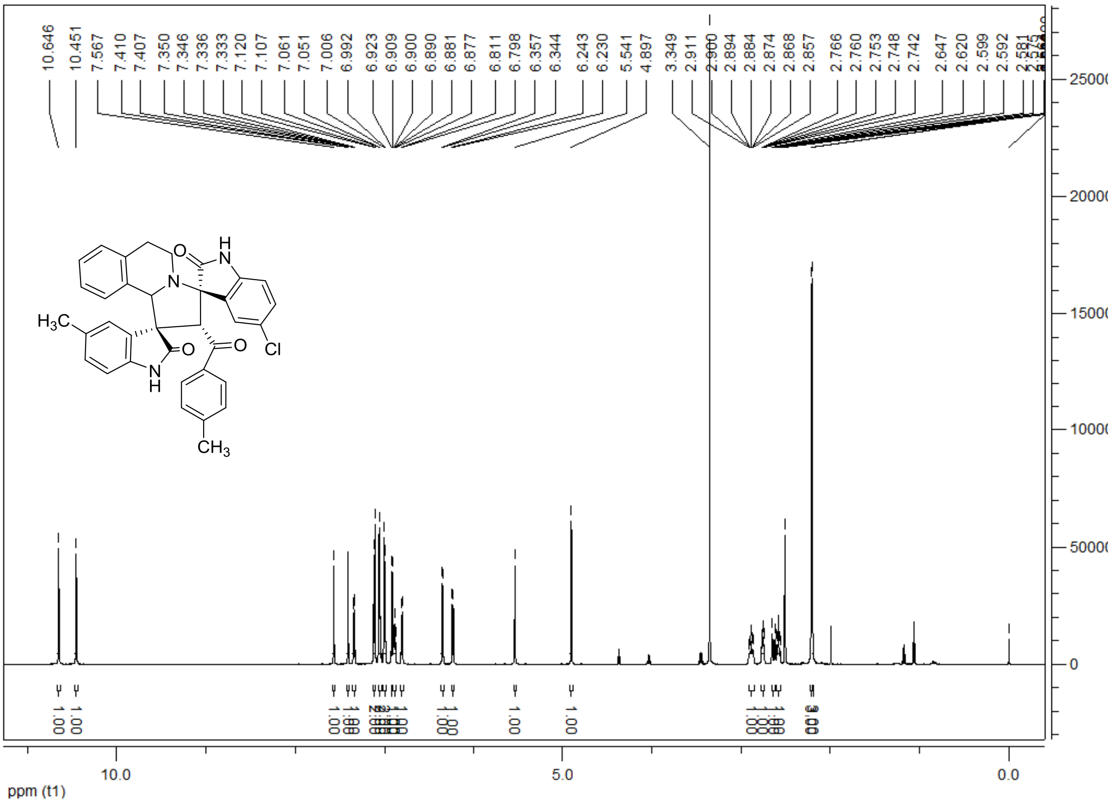

Copy of ^{1}H NMR spectrum of **4c** in DMSO-d_6

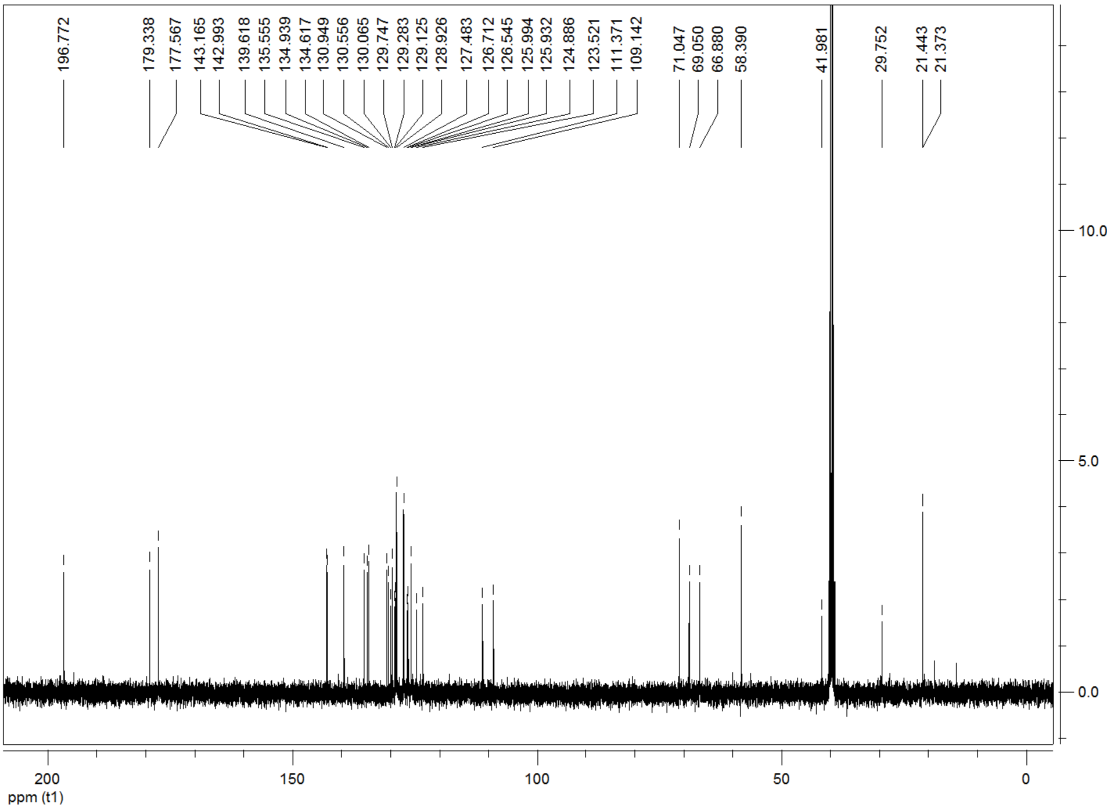

Copy of ^{13}C NMR spectrum of **4c** in DMSO-d_6

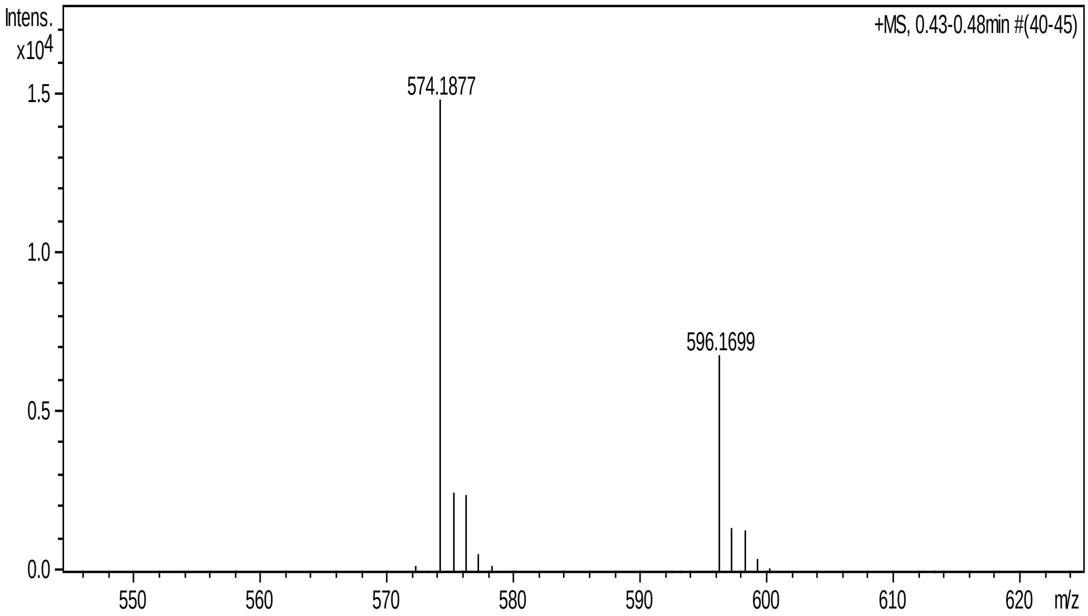

Copy of HRMS of **4c**

2'-Benzoyl-5''-methyl-6',10b'-dihydro-2'H,5'H-dispiro[indoline-3,1'-pyrrolo[2,1-a]isoquinoline-3',3''-indoline]-2,2''-dione (4d)

white solid, 90%, m.p. 246~247℃; ^{1}H NMR (600 MHz, DMSO-d_6) δ: 10.54 (s, 1H, NH), 10.46 (s, 1H, NH), 7.80 (d, *J* = 1.8Hz, 1H, ArH), 7.39~7.36 (m, 1H, ArH), 7.26 (s, 1H, ArH), 7.20~7.15 (m, 4H, ArH), 7.09~7.05 (m, 3H, ArH), 7.00~6.97 (m, 1H, ArH), 6.92~6.88 (m, 2H, ArH), 6.78 (d, *J* = 7.8Hz, 1H, ArH), 6.42 (d, *J* = 7.8Hz, 1H, ArH), 6.22 (d, *J* = 7.8Hz, 1H, ArH), 5.60 (s, 1H, CH), 4.93 (s, 1H, CH), 2.88~2.84 (m, 1H, CH), 2.81~2.79 (m, 1H, CH), 2.64~2.58 (m, 2H, CH), 2.30 (s, 3H, CH_3); ^{13}C NMR (100 MHz, DMSO-d_6) δ: 197.3, 179.5, 177.8, 142.0, 141.5, 137.0, 135.6, 135.0, 132.9, 130.8, 130.6, 130.4, 129.2, 128.8, 128.4, 127.5, 127.1, 126.7, 126.0, 125.9, 125.3, 123.5, 122.2, 109.6, 109.3, 71.1, 69.0, 66.7, 58.5, 41.9, 29.7, 21.1; IR(KBr) υ: 3402, 3177, 3079, 3033, 2919, 2839, 1712, 1622, 1493, 1448, 1421, 1342, 1293, 1246, 1213, 1176, 1010, 945, 809, 750cm^{-1}; MS (*m/z*): HRMS (ESI) Calcd. for $C_{34}H_{28}N_3O_3$ ($[M+H]^+$): 526.2115, found: 526.2117.

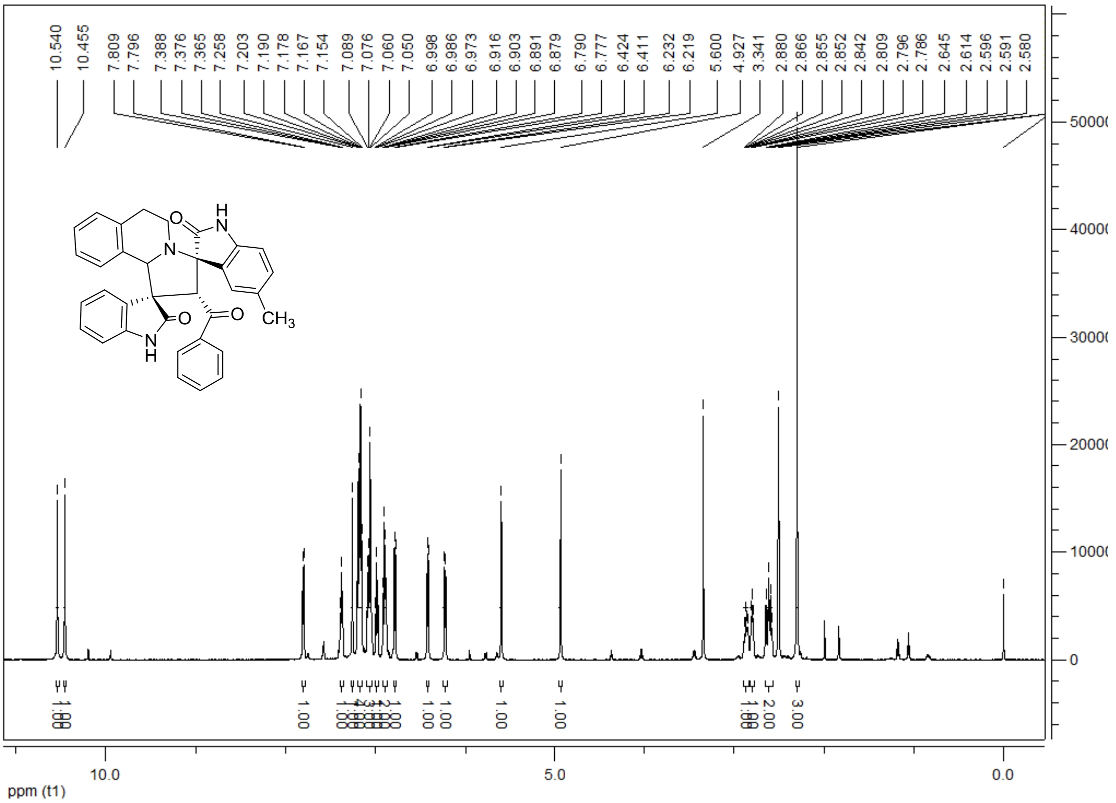

Copy of ^{1}H NMR spectrum of **4d** in DMSO-d_6

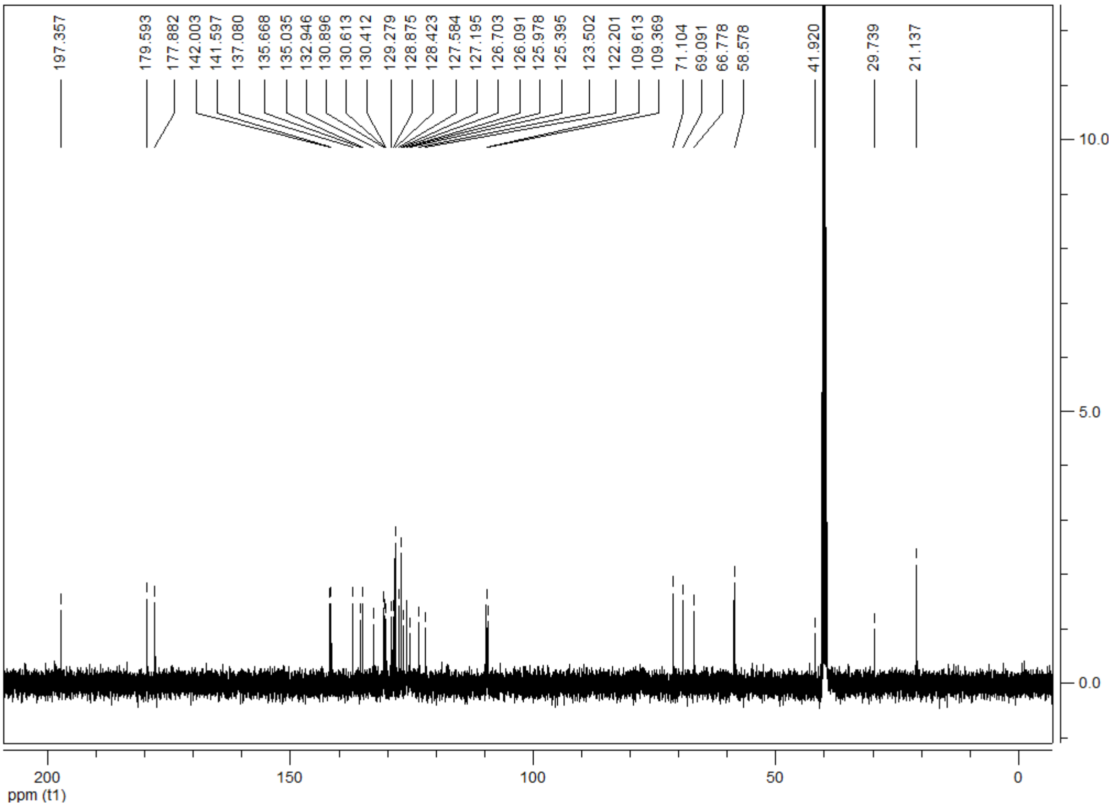

Copy of ^{13}C NMR spectrum of **4d** in DMSO-d_6

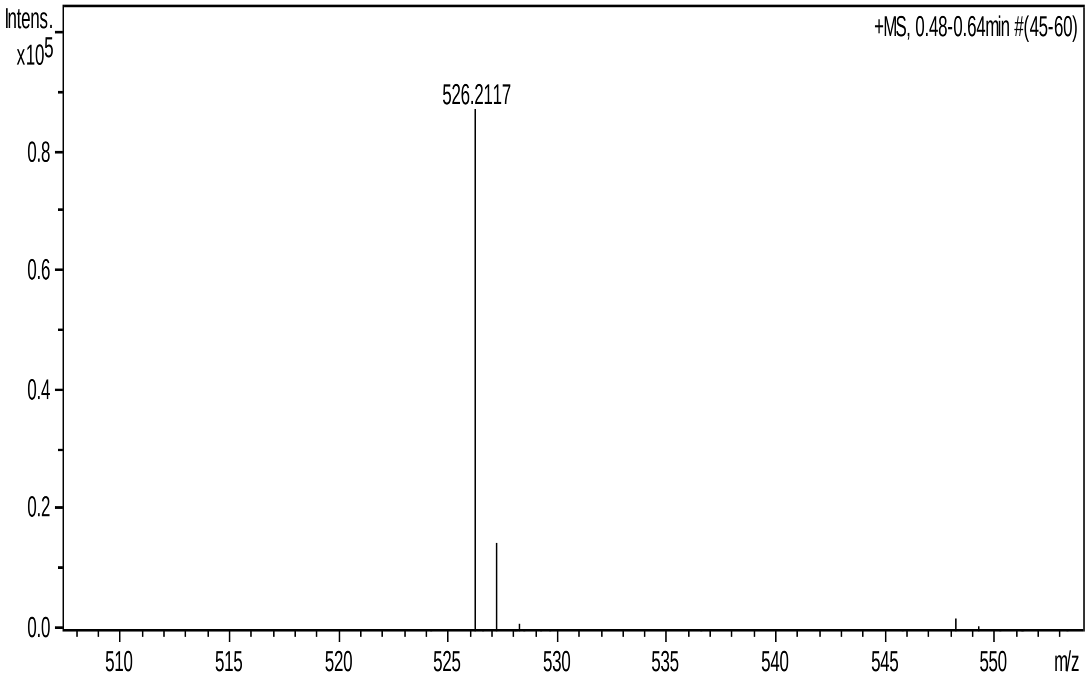

Copy of HRMS of **4d**

2'-(4-Chlorobenzoyl)-5,5''-dimethyl-6',10b'-dihydro-2'H,5'H-dispiro[indoline-3,1'-pyrrolo[2,1-a]isoquinoline-3',3''-indoline]-2,2''-dione (4e)

white solid, 80%, m.p. 235~237℃; ^{1}H NMR (400 MHz, DMSO-*d*$_6$) δ: 10.38 (brs, 2H, NH), 7.62 (s, 1H, ArH), 7.26~7.24 (m, 3H, ArH), 7.14~7.05 (m, 5H, ArH), 6.89~6.88 (m, 1H, ArH), 6.84~6.78 (m, 2H, ArH), 6.35 (d, *J* = 8.0Hz, 1H, ArH), 6.23 (d, *J* = 7.6Hz, 1H, ArH), 5.56 (s, 1H, CH), 4.88 (s, 1H, CH), 2.86~2.76 (m, 2H, CH), 2.64~2.58 (m, 2H, CH), 2.29 (s, 3H, CH_3), 2.22 (s, 3H, CH_3); ^{13}C NMR (100 MHz, DMSO-*d*$_6$) δ:196.7, 179.3, 177.7, 141.5, 139.5, 137.5, 136.0, 135.6, 134.9, 131.1, 130.9, 130.6, 130.4, 129.2, 129.1, 128.9, 128.4, 127.5, 126.6, 126.6, 125.9, 125.4, 123.5, 109.6, 109.1, 70.9, 68.9, 66.9, 58.3, 41.9, 29.7, 21.3, 21.1; IR(KBr) υ: 3378, 3172, 3032, 2946, 2910, 2835, 1716, 1680, 1626, 1589, 1493, 1428, 1336, 1294, 1246, 1202, 1166, 1090, 1040, 1006, 945, 809, 756, 731cm^{-1}; MS (*m*/*z*): HRMS (ESI) Calcd. for $C_{35}H_{29}ClN_3O_3$ ([M+H]$^+$): 574.1892, found: 574.1884.

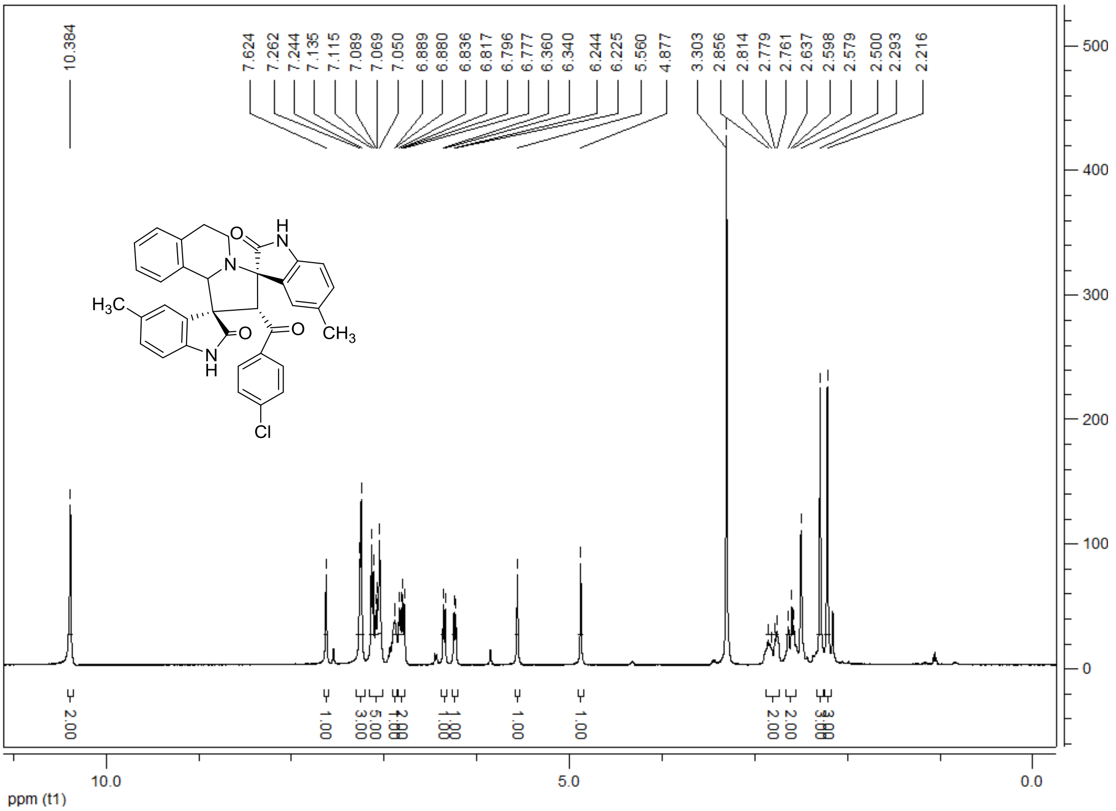

Copy of ^{1}H NMR spectrum of **4e** in DMSO-*d*$_6$

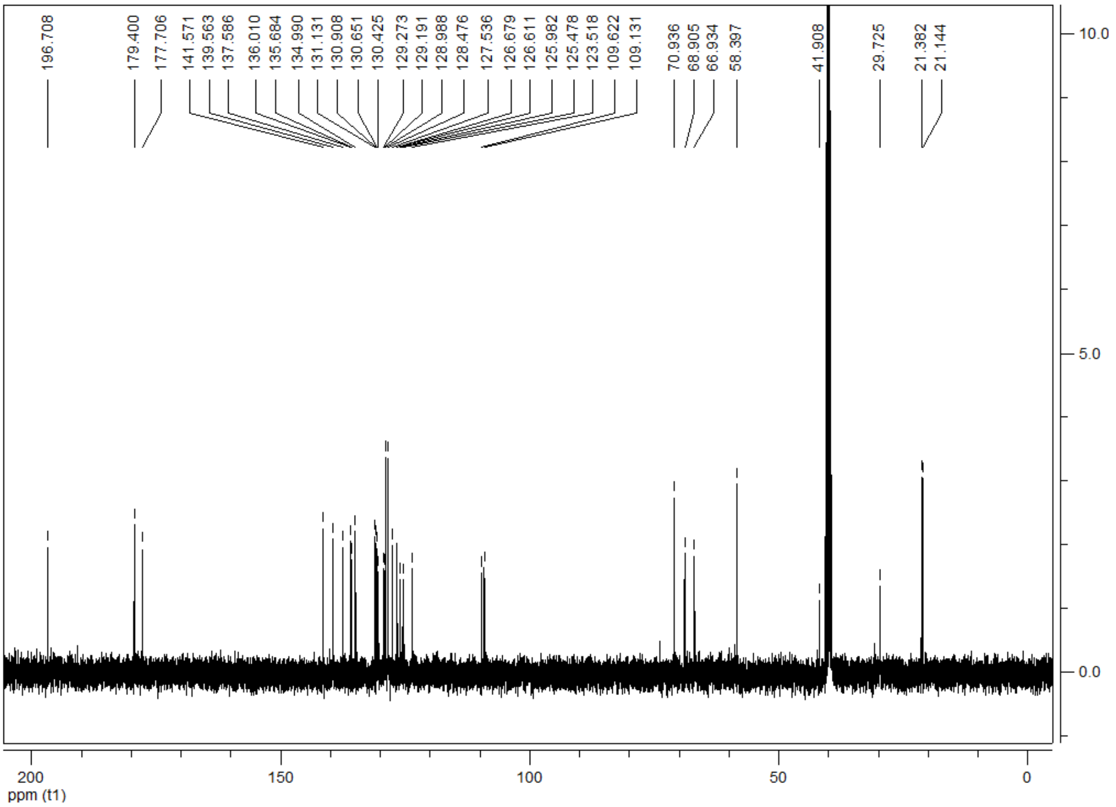

Copy of ^{13}C NMR spectrum of **4e** in DMSO-d_6

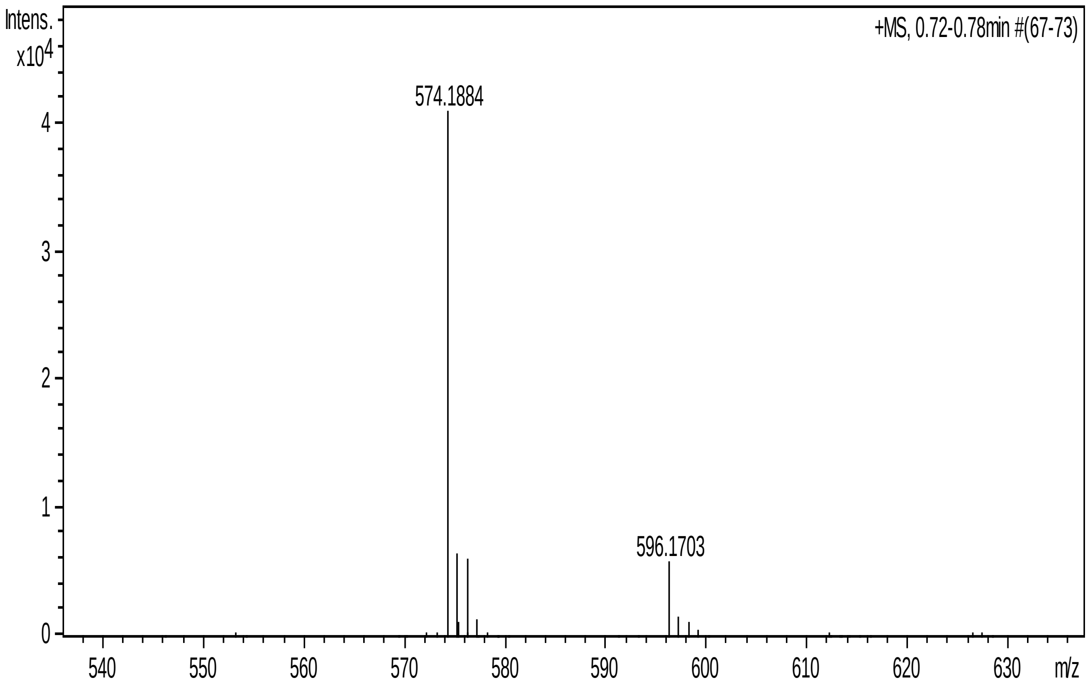

Copy of HRMS of **4e**

5-Chloro-5''-methyl-2'-(4-methylbenzoyl)-6',10b'-dihydro-2'H,5'H-dispiro[indoline-3, 1'-pyrrolo[2,1-a]isoquinoline-3',3''-indoline]-2,2''-dione (4f)

white solid, 50%, m.p. 237~239℃; ^1H NMR (400 MHz, DMSO-d_6) δ: 10.65 (s, 1H, NH), 10.44 (s, 1H, NH), 7.88~7.87 (m, 1H, ArH), 7.25 (s, 1H, ArH), 7.14~7.12 (m, 2H, ArH), 7.10~7.04 (m, 6H, ArH), 6.94~6.90 (m, 1H, ArH), 6.80 (d, J = 8.0Hz, 1H, ArH), 6.48 (d, J = 8.4Hz, 1H, ArH), 6.22 (d, J = 8.0Hz, 1H, ArH), 5.58 (s, 1H, CH), 4.91 (s, 1H, CH), 2.86~2.82 (m, 2H, CH), 2.66~2.61 (m, 2H, CH), 2.29 (s, 3H, CH_3), 2.23 (s, 3H, CH_3); ^{13}C NMR (100 MHz, DMSO-d_6) δ:196.5, 179.2, 178.0, 143.5, 141.5, 140.9, 135.2, 135.0, 134.5, 132.7, 130.9, 130.5, 129.3, 129.1, 128.7, 127.3, 127.2, 126.8, 126.2, 126.2, 126.1, 125.4, 123.4, 110.8, 109.7, 71.3, 69.2, 66.4, 59.0, 41.9, 29.6, 21.4, 21.1; IR(KBr) υ: 3353, 3179, 3028, 2918, 2820, 1714, 1679, 1620, 1494, 1474, 1441, 1342, 1291, 1247, 1183, 1079, 1039, 1008, 944, 900, 813, 750, 728cm^{-1}; MS (m/z): HRMS (ESI) Calcd. for $C_{35}H_{29}ClN_3O_3$ ($[M+H]^+$): 574.1892, found: 574.1879.

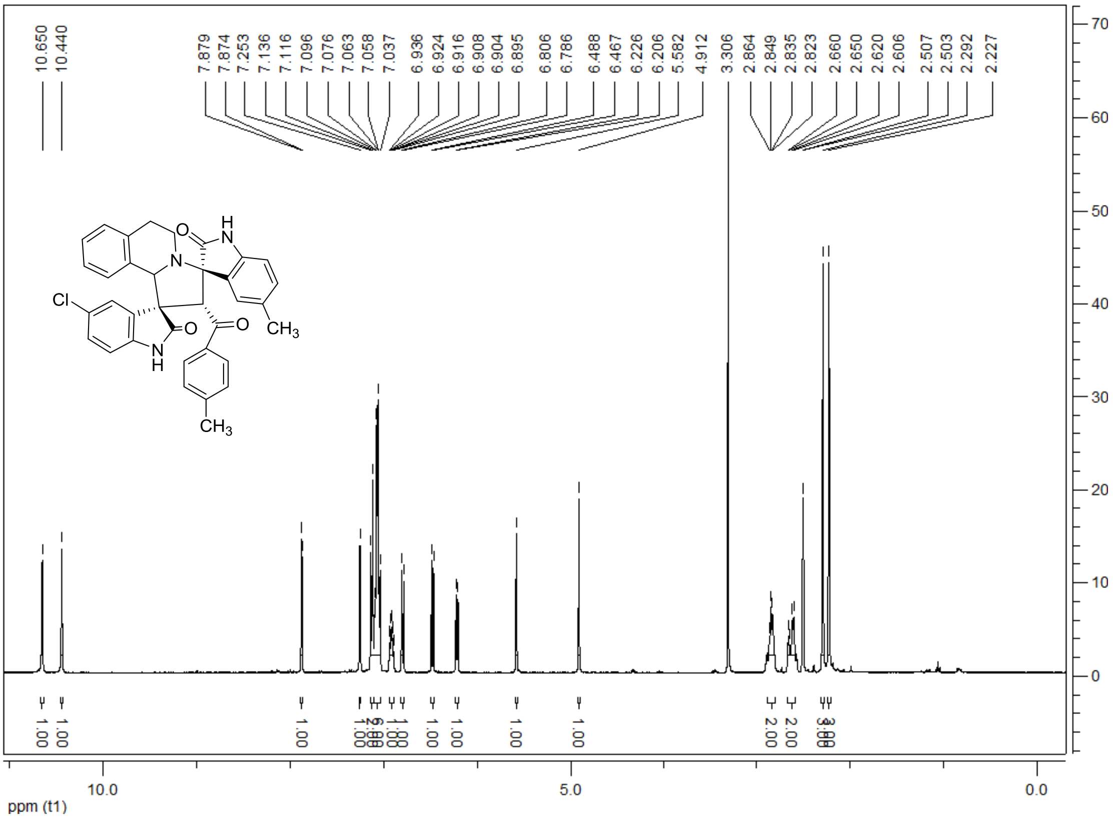

Copy of ^1H NMR spectrum of **4f** in DMSO-d_6

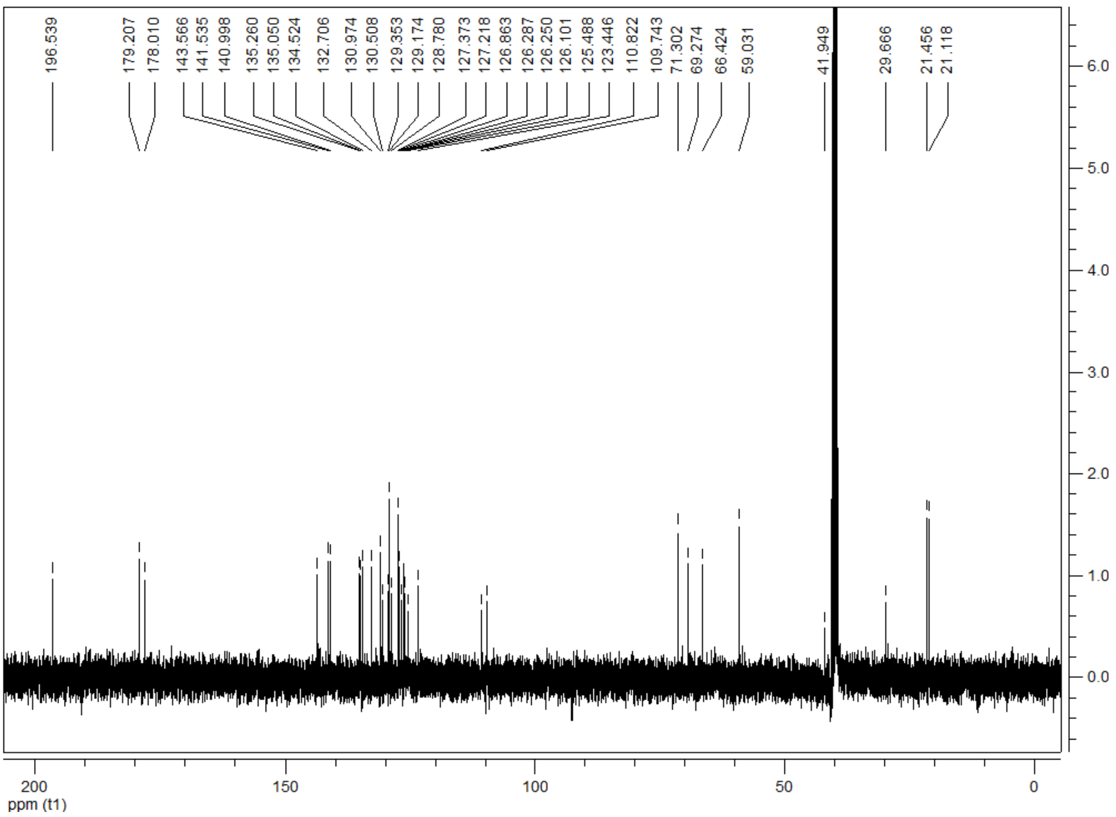

Copy of ^{13}C NMR spectrum of **4f** in DMSO-d_6

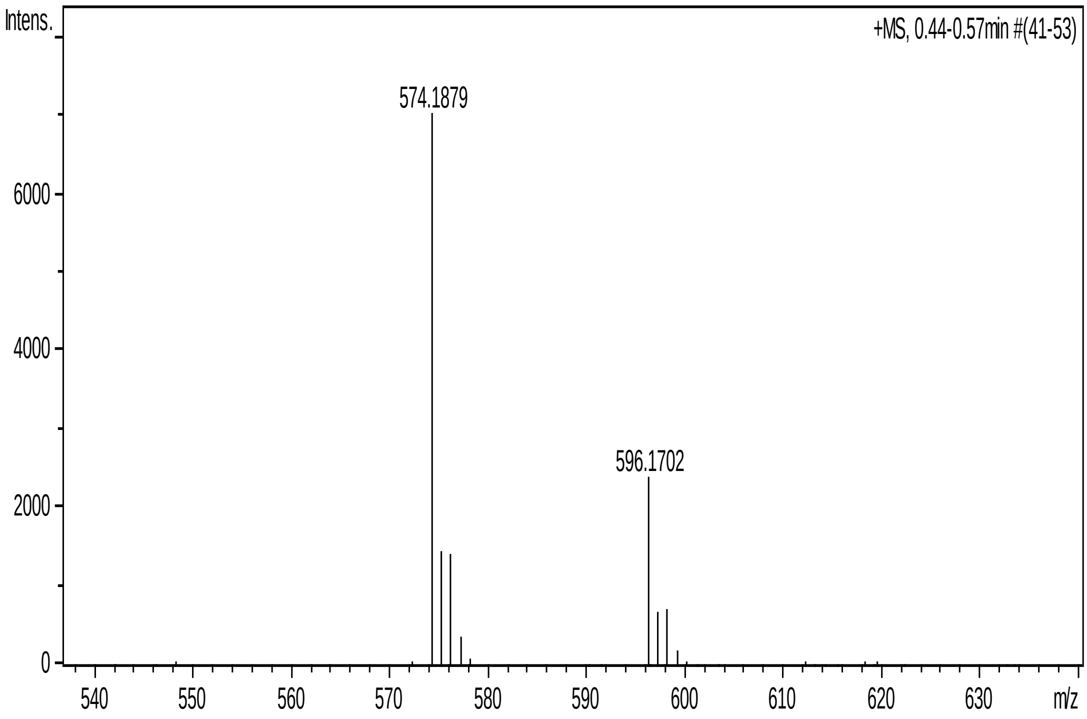

Copy of HRMS of **4f**

5''-Chloro-2'-(4-chlorobenzoyl)-5-methyl-6',10b'-dihydro-2'H,5'H-dispiro[indoline-3,1'-pyrrolo[2,1-a]isoquinoline-3',3''-indoline]-2,2''-dione (4g)

white solid, 82%, m.p. 236~237℃; ^{1}H NMR (400 MHz, DMSO-d_6) δ: 10.64 (s, 1H, NH), 10.40 (s, 1H, NH), 7.57 (brs, 1H, ArH), 7.44~7.43 (m, 1H, ArH), 7.34 (dd, J_1= 8.4Hz, J_2= 2.0Hz, 1H, ArH), 7.24~7.22 (m, 2H, ArH), 7.17~7.15 (m, 2H, ArH), 7.06~7.04 (m, 2H, ArH), 6.92~6.90 (m, 1H, ArH), 6.90~6.86 (m, 1H, ArH), 6.83~6.81 (m, 1H, ArH), 6.34 (d, J = 7.6Hz, 1H, ArH), 6.23 (d, J = 7.6Hz, 1H, ArH), 5.51 (s, 1H, CH), 4.92 (s, 1H, CH), 2.90~2.84 (m, 1H, CH), 2.76~2.72 (m, 1H, CH), 2.65~2.56 (m, 2H, CH), 2.21 (s, 3H, CH_3); ^{13}C NMR (100 MHz, DMSO-d_6) δ: 196.9, 179.1, 177.4, 142.9, 139.6, 137.5, 135.9, 135.5, 134.9, 131.1, 130.5, 130.0, 129.5, 129.3, 129.2, 129.1, 128.3, 126.7, 126.3, 126.0, 125.9, 125.2, 123.4, 111.3, 109.2, 70.9, 68.7, 67.0, 58.0, 41.9, 29.7, 21.3; IR(KBr) υ: 3440, 3250, 2915, 2831, 1716, 1622, 1589, 1483, 1437, 1401, 1343, 1290, 1258, 1204, 1091, 1010, 943, 851, 813, 755cm^{-1}; MS (*m*/*z*): HRMS (ESI) Calcd. for $C_{34}H_{26}Cl_2N_3O_3$ ([M+H]$^+$): 594.1346, found: 594.1337.

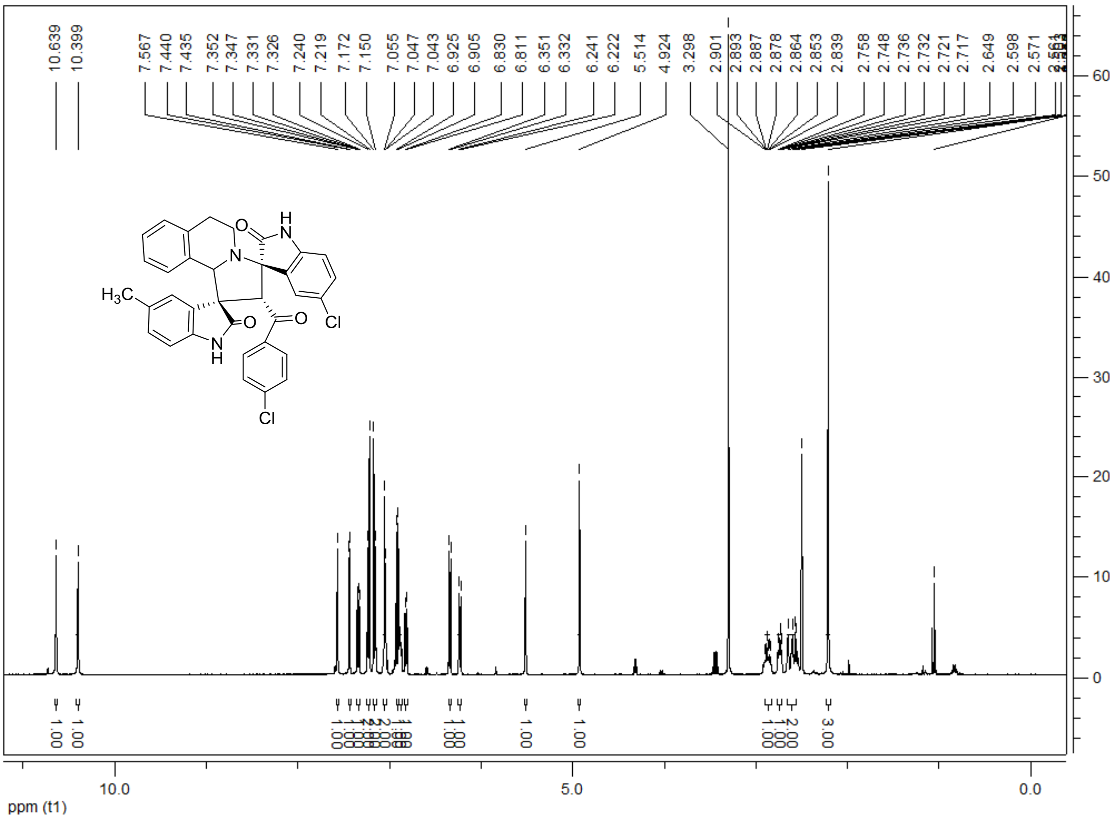

Copy of ^{1}H NMR spectrum of **4g** in DMSO-d_6

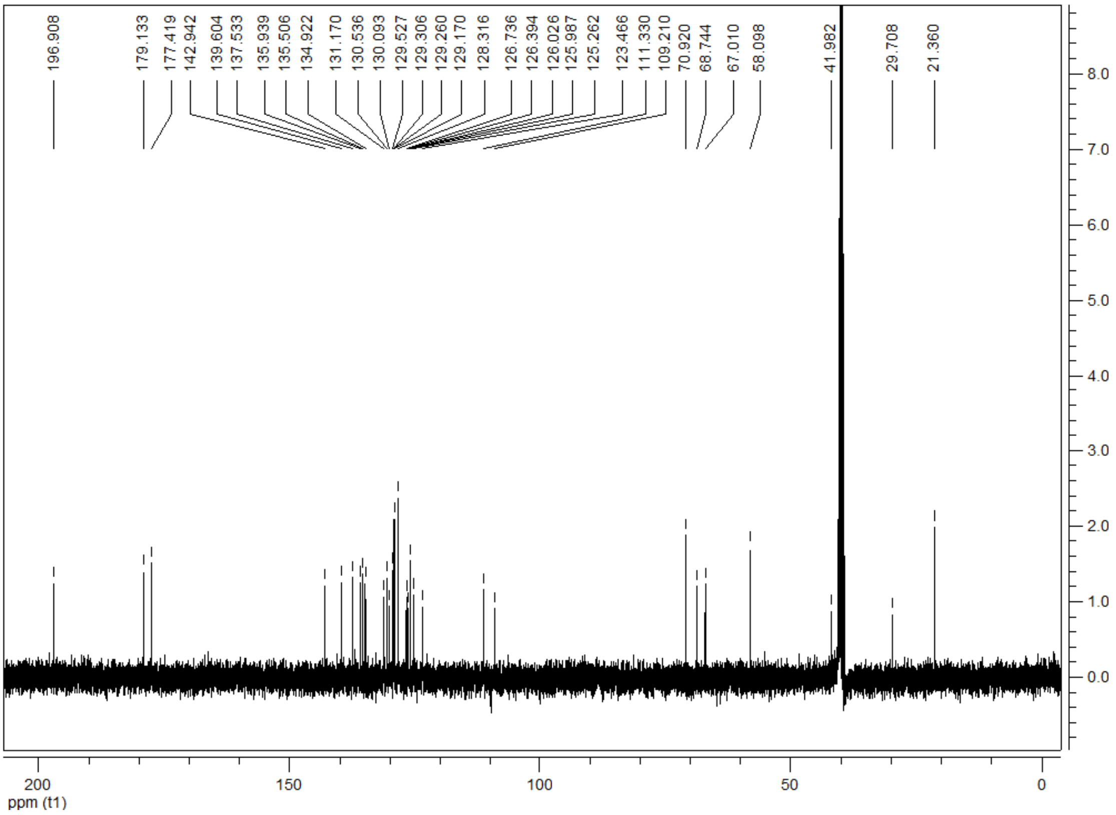

Copy of ^{13}C NMR spectrum of **4g** in DMSO-d_6

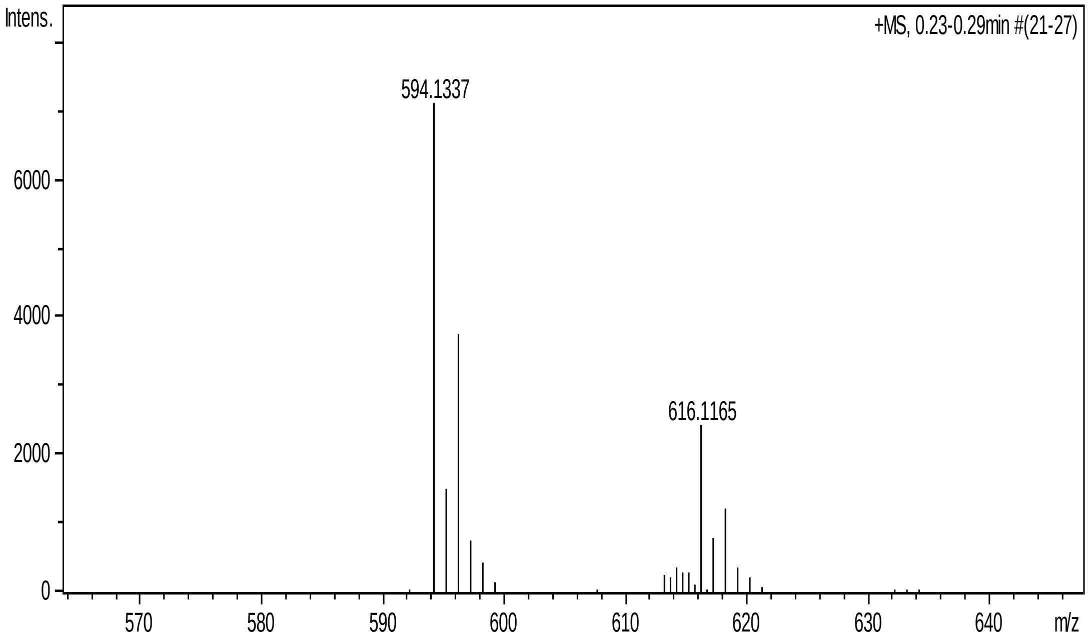

Copy of HRMS of **4g**

5,5''-Dichloro-2'-(4-methoxybenzoyl)-6',10b'-dihydro-2'H,5'H-dispiro[indoline-3,1'-pyrrolo[2,1-a]isoquinoline-3',3''-indoline]-2,2''-dione (4h)

white solid, 67%, m.p. 244~245℃; ^{1}H NMR (400 MHz, DMSO-d_6) δ: 10.67 (s, 2H, 2NH), 7.81 (brs, 1H, ArH), 7.43 (brs, 1H, ArH), 7.35~7.33 (m, 1H, ArH), 7.26~7.24 (m, 2H, ArH), 7.08~7.07 (m, 3H, ArH), 6.93~6.91 (m, 2H, ArH), 6.77~6.75 (m, 2H, ArH), 6.52~6.50 (m, 1H, ArH), 6.23~6.21 (m, 1H, ArH), 5.54 (s, 1H, CH), 4.91 (s, 1H, CH), 3.72 (s, 3H, OCH_3), 2.88~2.78 (m, 2H, CH), 2.66~2.60 (m, 2H, CH); ^{13}C NMR (100 MHz, DMSO-d_6) δ: 195.2, 179.0, 177.7, 163.2, 142.9, 141.0, 135.1, 135.0, 132.6, 130.2, 129.8, 129.7, 129.4, 129.2, 128.9, 126.9, 126.3, 126.2, 126.1, 126.0, 125.2, 123.4, 113.9, 111.5, 111.0, 71.3, 69.1, 66.5, 58.8, 55.9, 42.0, 29.6; IR(KBr) υ: 3357, 3172, 2947, 2842, 1720, 1657, 1600, 1571, 1476, 1438, 1330, 1289, 1246, 1219, 1176, 1073, 1031, 983, 942, 897, 844, 810, 730cm^{-1}; MS (*m*/*z*): HRMS (ESI) Calcd. for $C_{34}H_{26}ClN_3O_4$ ($[M+H]^+$): 610.1295, found: 610.1293.

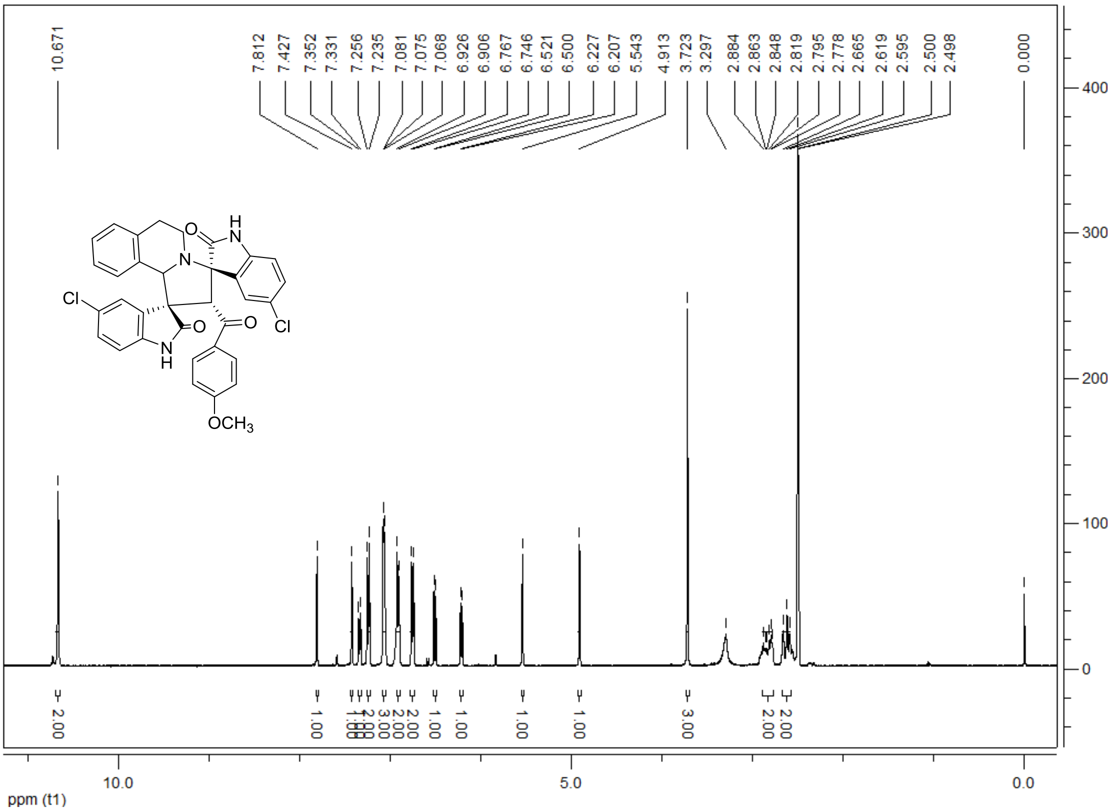

Copy of ^{1}H NMR spectrum of **4h** in DMSO-d_6

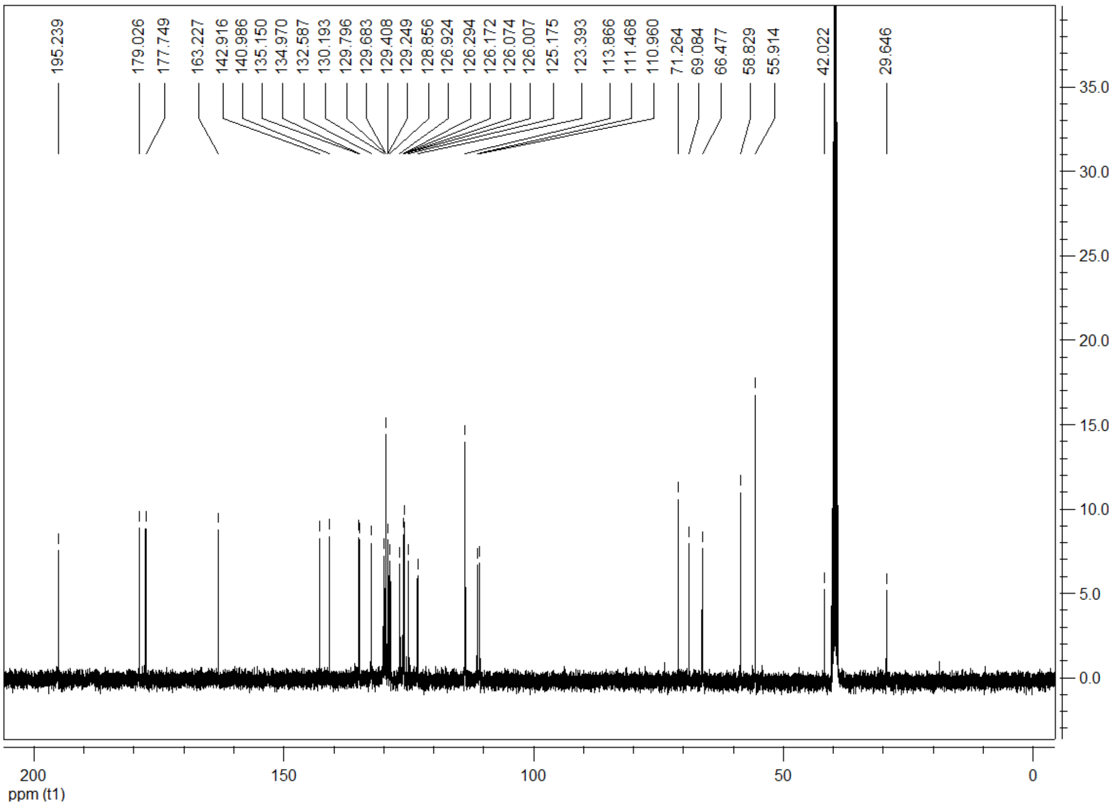

Copy of ^{13}C NMR spectrum of **4h** in DMSO-d_6

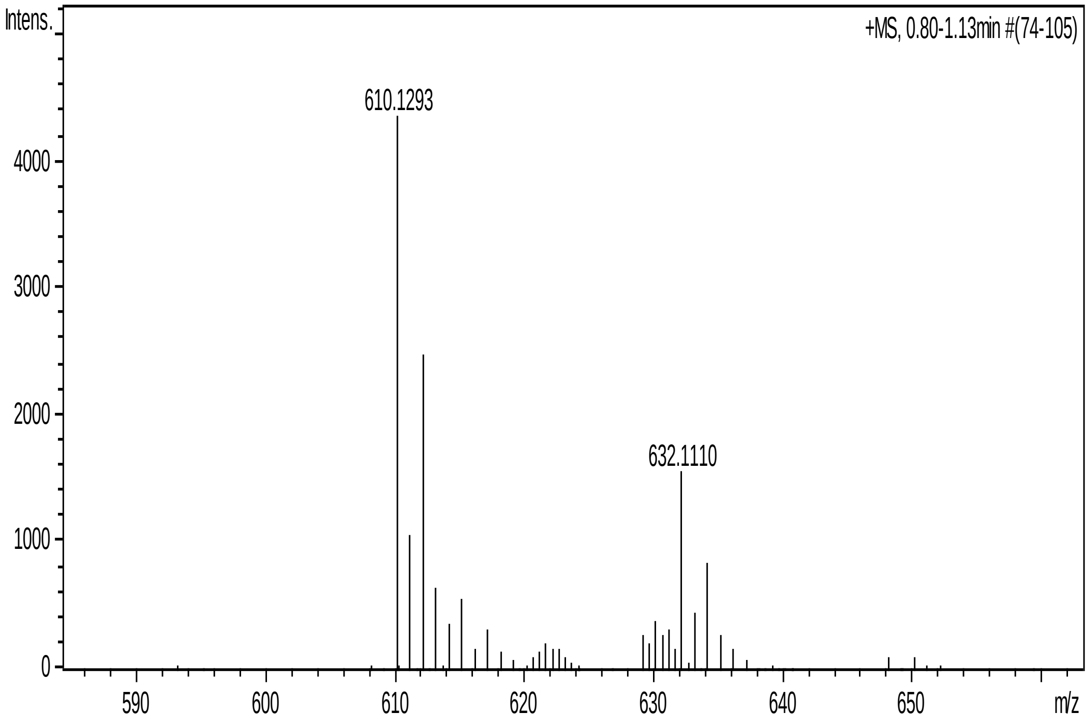

Copy of HRMS of **4h**

5''-Chloro-5-fluoro-2'-(4-methylbenzoyl)-6',10b'-dihydro-2'H,5'H-dispiro[indoline-3,1'-pyrrolo[2,1-a]isoquinoline-3',3''-indoline]-2,2''-dione (4i)

white solid, 57%, m.p. 243~244℃; ^{1}H NMR (400 MHz, DMSO-*d*$_6$) δ: 10.72 (s, 1H, NH), 10.56 (s, 1H, NH), 7.63~7.60 (m, 1H, ArH), 7.44 (brs, 1H, ArH), 7.36~7.33 (m, 1H, ArH), 7.16 (d, *J* = 8.0Hz, 2H, ArH), 7.08~7.02 (m, 4H, ArH), 6.93~6.91 (m, 2H, ArH), 6.87~6.82 (m, 1H, ArH), 6.46~6.43 (m, 1H, ArH), 6.22 (d, *J* = 8.0Hz, 1H, ArH), 5.52 (s, 1H, CH), 4.95 (s, 1H, CH), 2.88~2.81 (m, 2H, CH), 2.66~2.59 (m, 2H, CH), 2.23 (s, 3H, CH_3); ^{13}C NMR (100 MHz, DMSO-*d*$_6$) δ: 196.7, 179.1, 177.7, 158.5 (d, *J* = 23.5Hz), 143.5, 142.9, 138.3, 138.3, 135.1, 134.9, 134.3, 132.3, 132.2, 130.1, 129.3, 129.2, 129.0, 127.5, 126.8, 126.1, 126.0, 125.1, 123.4, 115.3 (d, *J* = 23.0Hz), 113.5 (d, *J* = 25.7Hz), 111.4, 110.3 (d, *J* = 8.2Hz), 71.3, 69.1, 66.5, 58.9, 42.0, 29.6, 21.4; IR(KBr) υ: 3341, 3180, 3066, 2925, 2823, 1715, 1676, 1607, 1481, 1344, 1287, 1258, 1185, 1073, 1038, 982, 946, 894, 815, 751, 727cm^{-1}; MS (*m*/*z*): HRMS (ESI) Calcd. for $C_{34}H_{26}ClFN_3O_3$ ([M+H]$^+$): 578.1641, found: 578.1635.

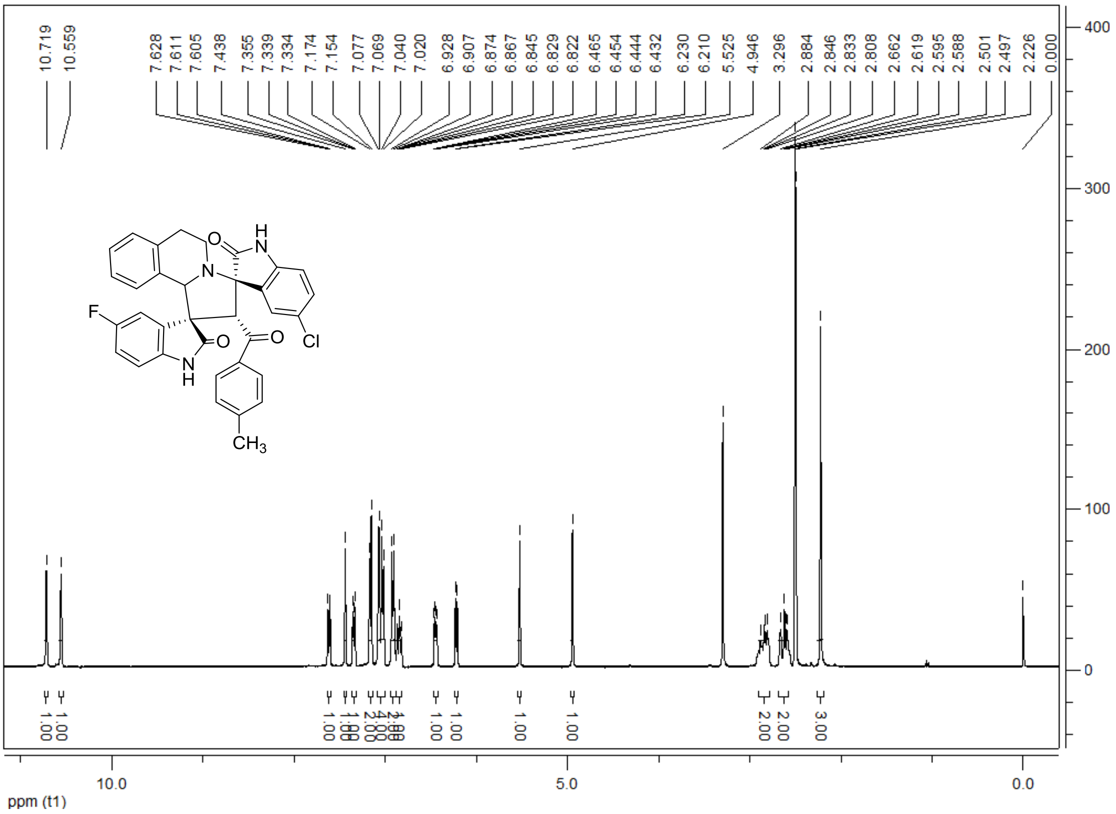

Copy of ^{1}H NMR spectrum of **4i** in DMSO-*d*$_6$

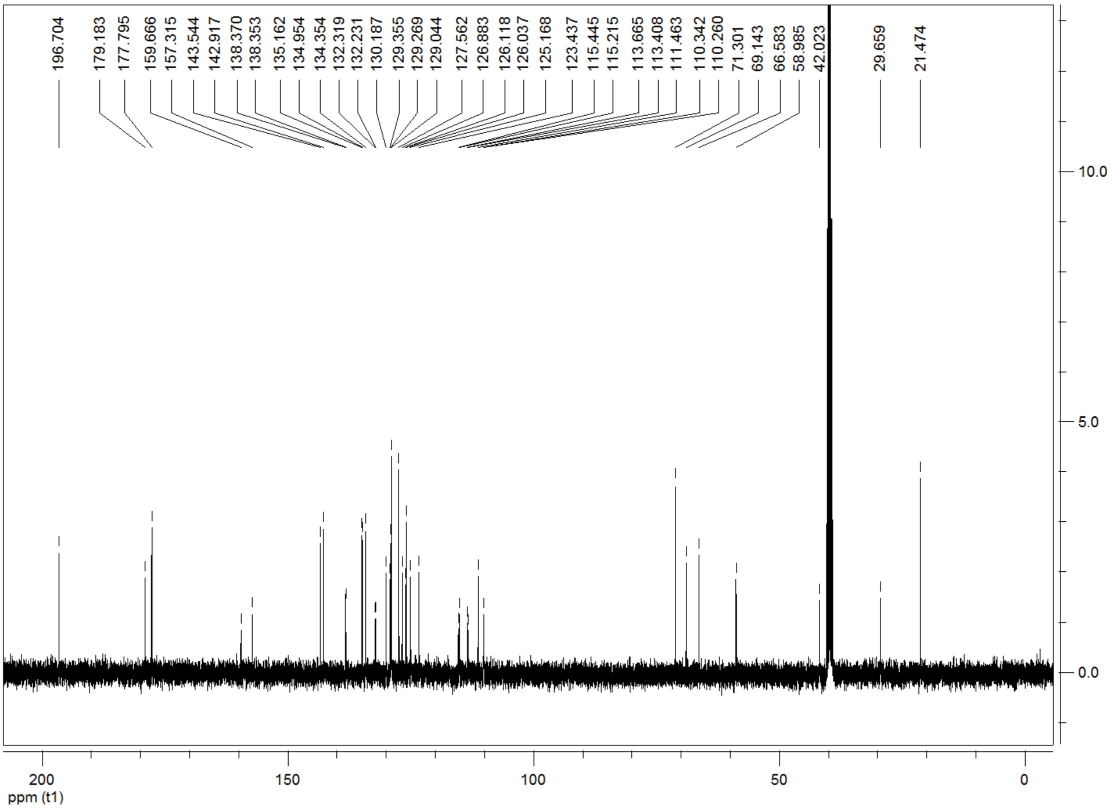

Copy of ^{13}C NMR spectrum of **4i** in DMSO-d_6

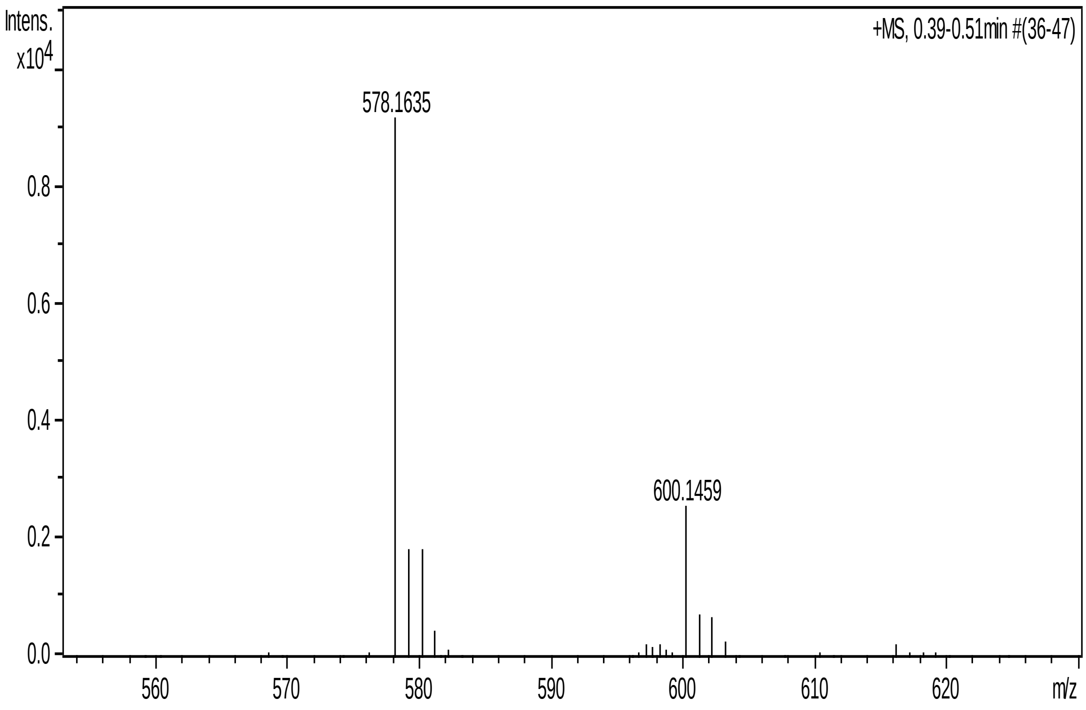

Copy of HRMS of **4i**

1-Benzyl-5-chloro-2'-(4-chlorobenzoyl)-5''-methyl-6',10b'-dihydro-2'H,5'H-dispiro[indoline-3,1'-pyrrolo[2,1-a]isoquinoline-3',3''-indoline]-2,2''-dione (4j)

white solid, 57%, m.p. 234~235℃; ^{1}H NMR (400 MHz, DMSO-d_6) δ: 10.50 (s, 1H, NH), 7.97~7.96 (m, 1H, ArH), 7.32~7.31 (m, 4H, ArH), 7.23~7.16 (m, 5H, ArH), 7.11~7.06 (m, 5H, ArH), 6.81 (d, J = 8.0Hz, 1H, ArH), 6.71 (d, J = 8.8Hz, 1H, ArH), 6.68~6.64 (m, 1H, ArH), 5.97 (d, J = 8.0Hz, 1H, ArH), 5.66 (s, 1H, CH), 4.97~4.92 (m, 2H, CH), 4.21 (d, J = 15.6Hz, 1H, CH), 2.92~2.86 (m, 2H, CH), 2.68~2.64 (m, 2H, CH), 2.31 (s, 3H, CH_3); ^{13}C NMR (150 MHz, DMSO-d_6) δ: 195.6, 177.4, 177.0, 141.2, 141.0, 137.7, 135.4, 135.0, 134.5, 134.0, 131.2, 130.5, 130.1, 129.0, 128.5, 128.5, 128.4, 128.2, 127.5, 126.9, 126.5, 126.3, 125.7, 125.6, 125.4, 123.2, 109.9, 109.3, 70.9, 68.6, 66.3, 57.9, 44.0, 41.5, 29.0, 20.6; IR(KBr) υ: 3434, 3282, 3066, 2922, 2819, 1710, 1609, 1587, 1493, 1429, 1348, 1281, 1211, 1172, 1119, 1088, 1008, 938, 855, 809, 735cm^{-1}; MS (m/z): HRMS (ESI) Calcd. for $C_{41}H_{32}Cl_2N_3O_3$ ($[M+H]^+$): 684.1815, found: 684.1811.

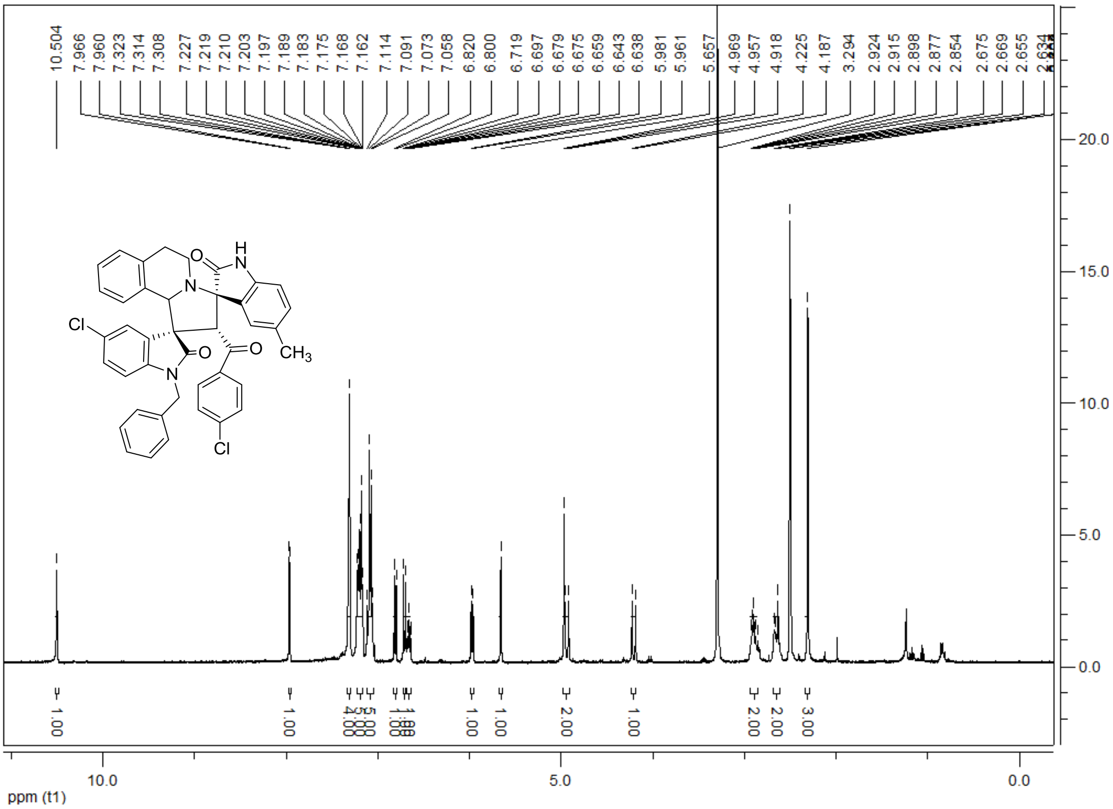

Copy of ^{1}H NMR spectrum of **4j** in DMSO-d_6

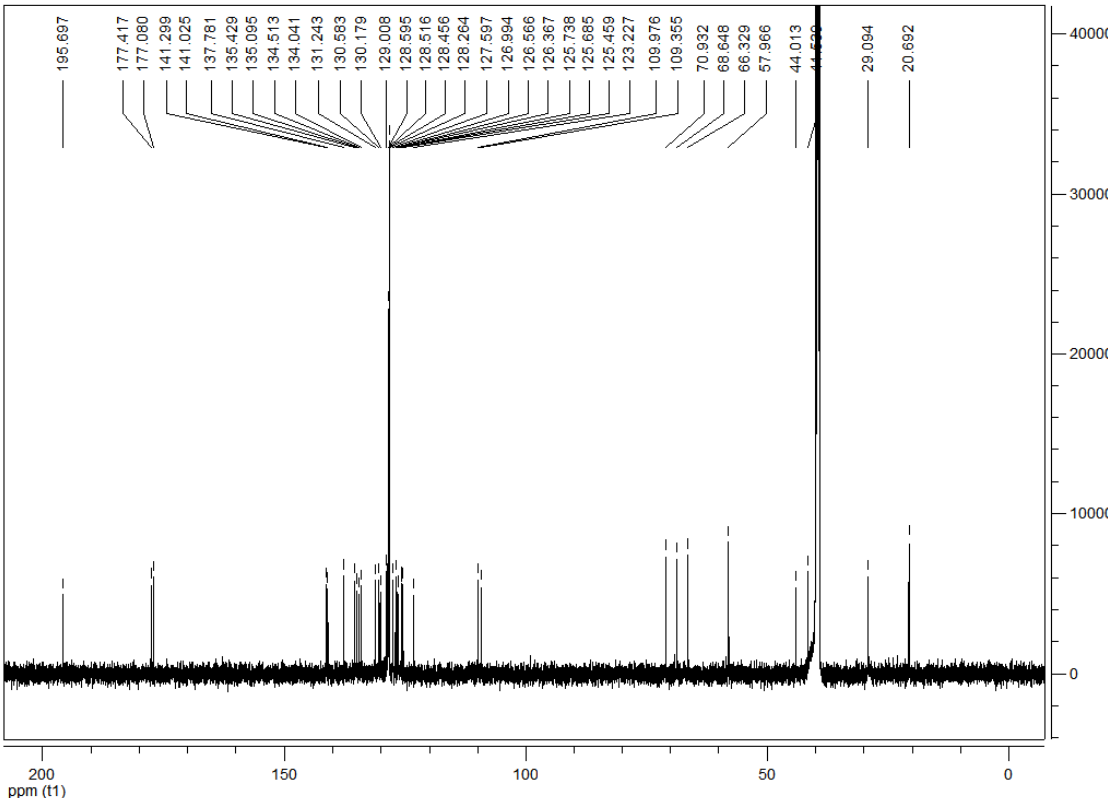

Copy of ^{13}C NMR spectrum of **4j** in DMSO-d_6

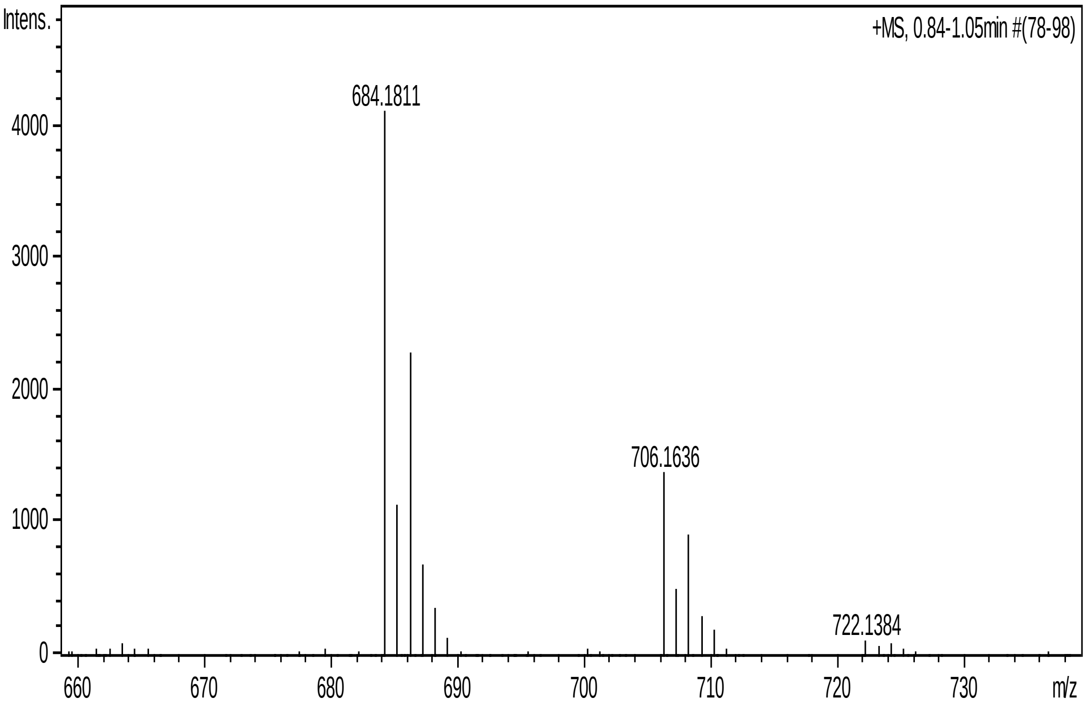

Copy of HRMS of **4j**

2'-Benzoyl-1-benzyl-5''-methyl-6',10b'-dihydro-2'H,5'H-dispiro[indoline-3,1'-pyrrolo[2,1-a]isoquinoline-3',3''-indoline]-2,2''-dione (4k)

white solid, 58%, m.p. 244~245℃; ^{1}H NMR (400 MHz, DMSO-d_6) δ: 10.45 (s, 1H, NH), 7.91~7.89 (m, 1H, ArH), 7.40~7.36 (m, 1H, ArH), 7.29~7.28 (m, 4H, ArH), 7.19~7.17 (m, 2H, ArH), 7.16~7.12 (m, 2H, ArH), 7.10~6.98 (m, 7H, ArH), 6.79 (d, J = 8.0Hz, 1H, ArH), 6.66~6.62 (m, 1H, ArH), 6.50 (d, J = 7.2Hz, 1H, ArH), 5.99 (d, J = 8.0Hz, 1H, ArH), 5.71 (s, 1H, CH), 5.01~4.97 (m, 2H, CH), 4.07 (d, J = 15.6Hz, 1H, CH), 2.92~2.86 (m, 2H, CH), 2.68~2.64 (m, 2H, CH), 2.31 (s, 3H, CH_3); ^{13}C NMR (100 MHz, DMSO-d_6) δ:197.1, 178.1, 177.8, 142.7, 141.5, 137.0, 136.2, 135.0, 134.9, 133.0, 130.9, 130.4, 129.7, 129.3, 128.9, 128.8, 128.4, 128.3, 127.8, 127.3, 127.0, 126.8, 126.0, 126.0, 125.5, 123.8, 123.1, 109.6, 108.8, 71.1, 68.9, 67.3, 58.3, 44.3, 42.0, 29.6, 21.1; IR(KBr) υ: 3344, 3057, 3028, 2909, 2838, 1715, 1611, 1491, 1465, 1364, 1300, 1221, 1175, 1002, 945, 848, 811, 750, 728cm^{-1}; MS (m/z): HRMS (ESI) Calcd. for $C_{41}H_{34}N_3O_3$ ($[M+H]^+$): 616.2595, found: 616.2589.

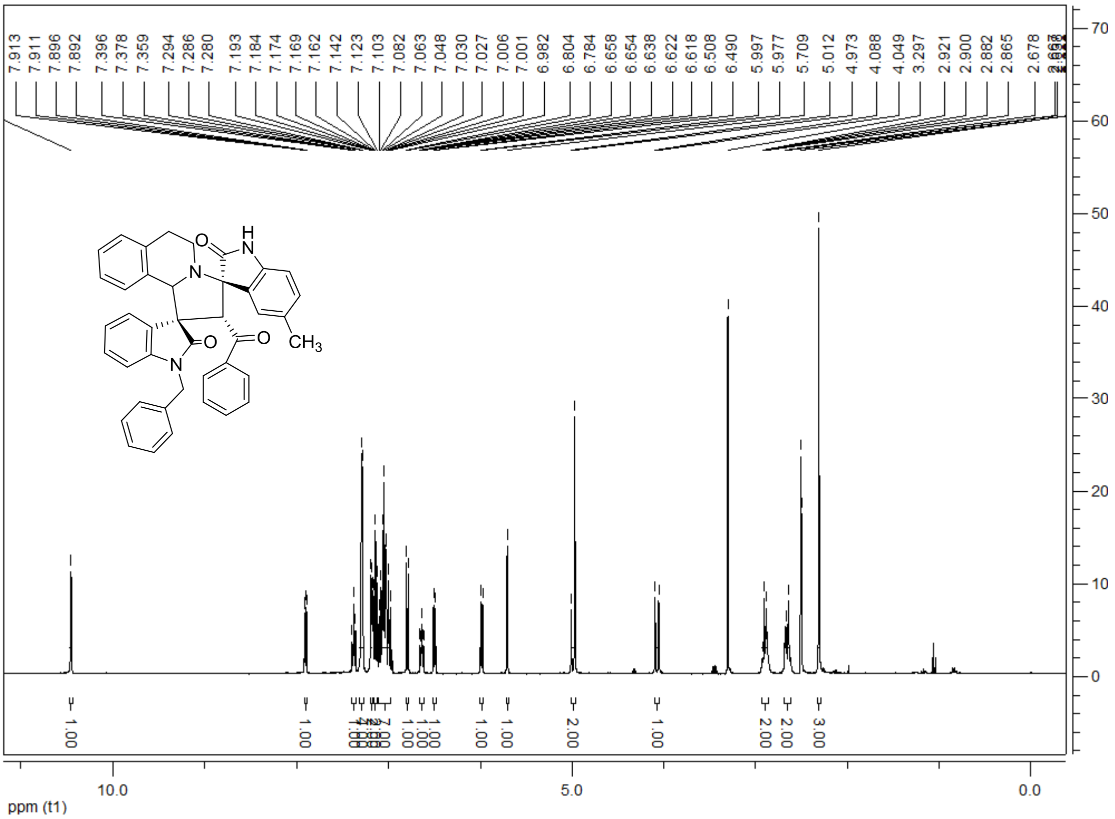

Copy of ^{1}H NMR spectrum of **4k** in DMSO-d_6

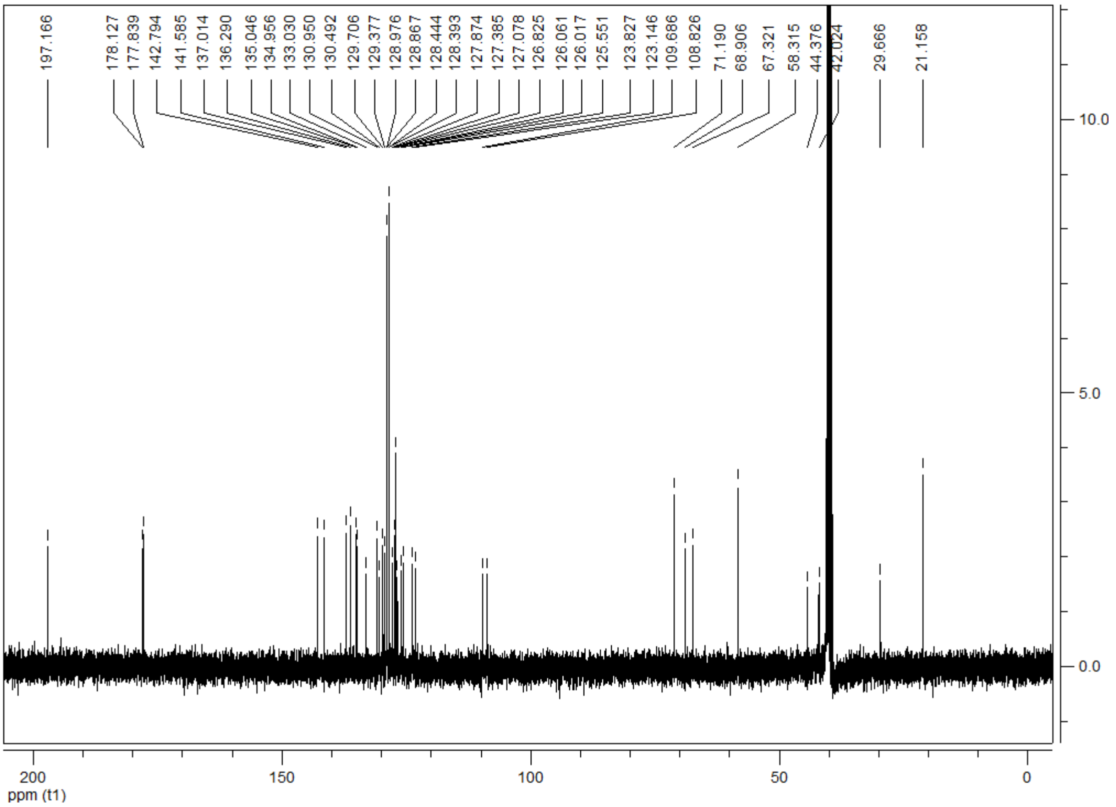

Copy of ^{13}C NMR spectrum of **4k** in DMSO-d_6

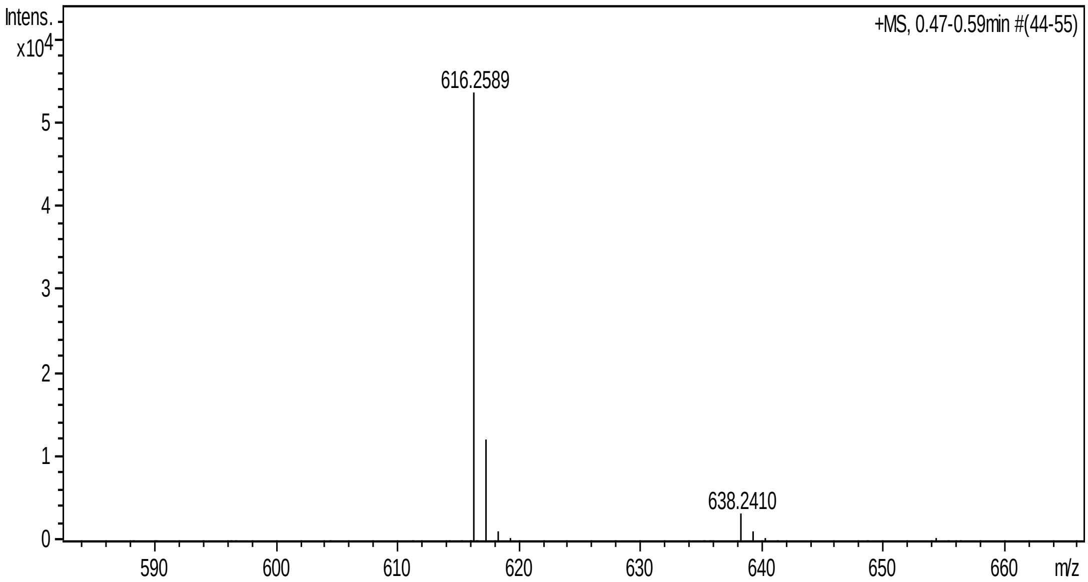

Copy of HRMS of **4k**

1-Benzyl-5,5''-dimethyl-2'-(4-methylbenzoyl)-6',10b'-dihydro-2'H,5'H-dispiro[indoline-3,1'-pyrrolo[2,1-a]isoquinoline-3',3''-indoline]-2,2''-dione (4l)

white solid, 62%, m.p. 243~244℃; ^{1}H NMR (400 MHz, DMSO-d_6) δ: 10.38 (s, 1H, NH), 7.71 (brs, 1H, ArH), 7.30~7.26 (m, 4H, ArH), 7.22~7.20 (m, 2H, ArH), 7.09~7.04 (m, 3H, ArH), 6.96~6.89 (m, 4H, ArH), 6.84 (d, J = 8.0Hz, 1H, ArH), 6.78 (d, J = 7.6Hz, 1H, ArH), 6.78~6.64 (m, 1H, ArH), 6.44 (d, J = 7.6Hz, 1H, ArH), 6.01 (d, J = 8.0Hz, 1H, ArH), 5.68 (s, 1H, CH), 4.94~4.90 (m, 2H, CH), 4.13 (d, J = 15.6Hz, 1H, CH), 2.88~2.84 (m, 2H, CH), 2.67~2.60 (m, 2H, CH), 2.30 (s, 3H, CH_3), 2.22 (s, 6H, CH_3); ^{13}C NMR (100 MHz, DMSO-d_6) δ: 196.5, 178.0, 177.8, 143.3, 141.5, 140.4, 136.3, 135.1, 134.9, 134.5, 131.9, 130.9, 130.4, 129.7, 129.3, 129.0, 128.9, 128.8, 128.5, 127.8, 127.4, 127.2, 126.7, 126.6, 126.0, 125.4, 123.8, 109.6, 108.5, 71.1, 68.9, 67.1, 58.3, 44.3, 42.0, 29.6, 21.4, 21.3, 21.1; IR(KBr) υ: 3434, 3274, 3030, 2918, 2838, 1712, 1684, 1624, 1604, 1494, 1432, 1407, 1360, 1303, 1199, 1169, 1121, 1031, 1005, 940, 859, 812, 727cm^{-1}; MS (m/z): HRMS (ESI) Calcd. for $C_{43}H_{38}N_3O_3$ ($[M+H]^+$): 644.2908, found: 644.2904.

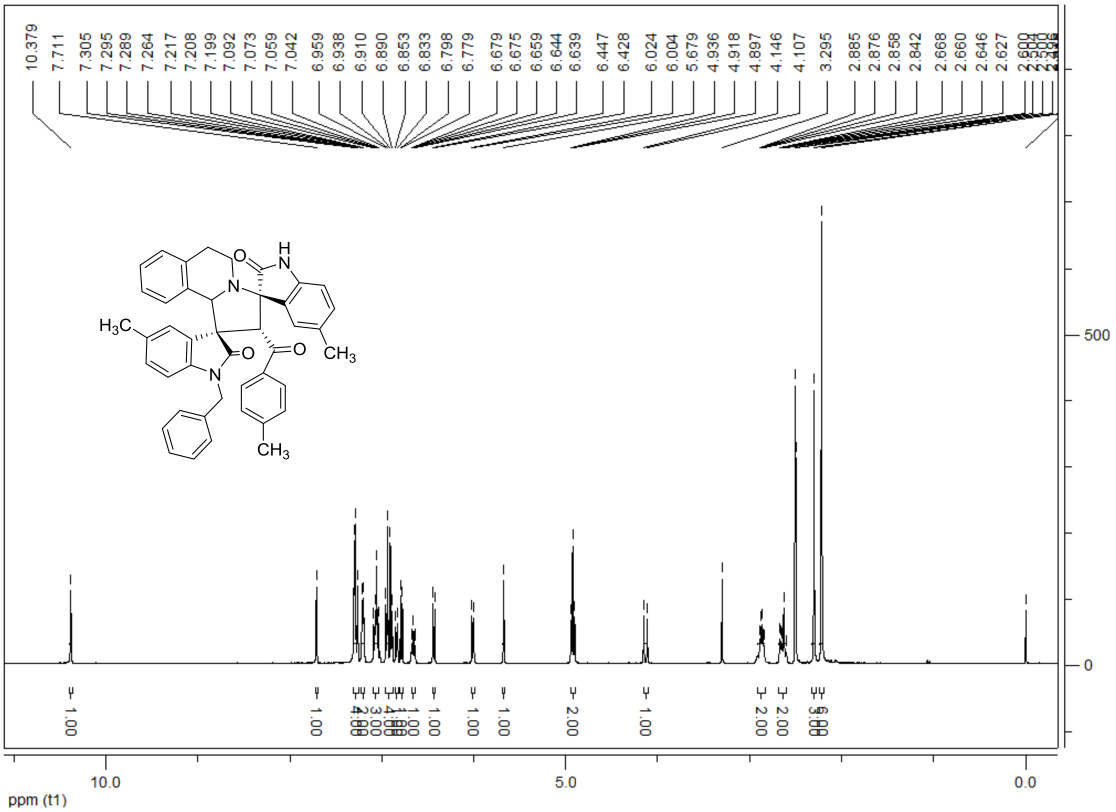

Copy of ^{1}H NMR spectrum of **4l** in DMSO-d_6

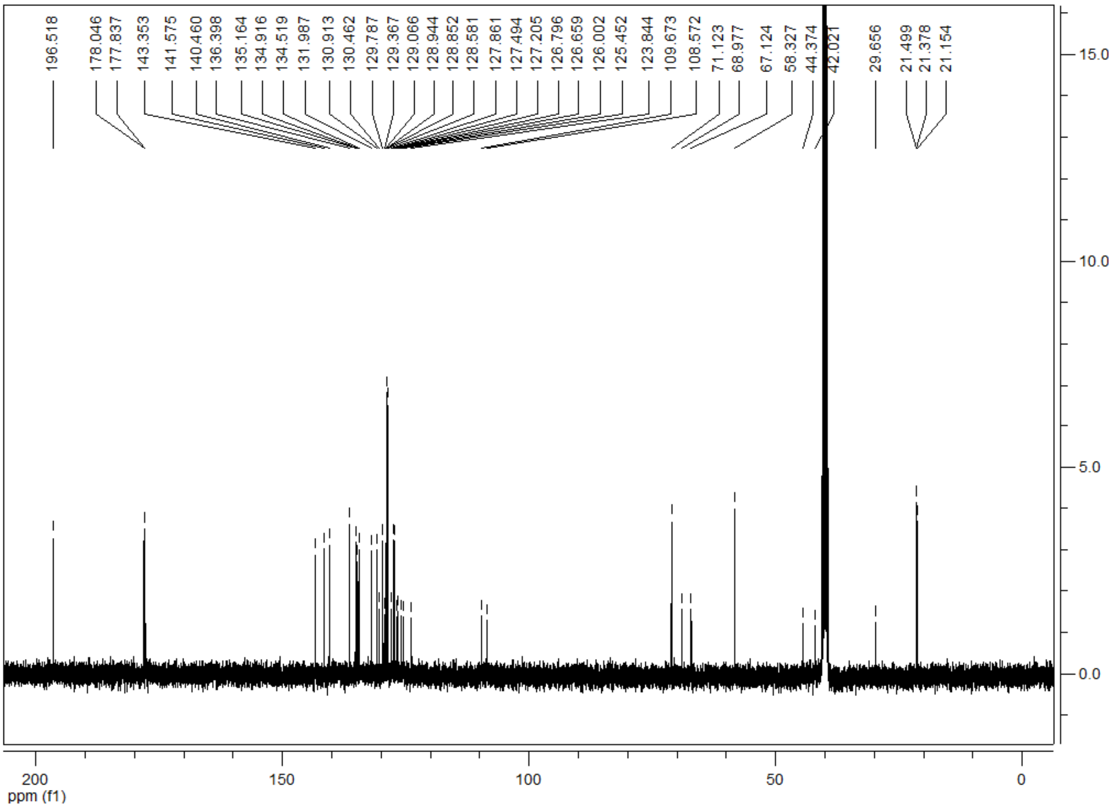

Copy of ^{13}C NMR spectrum of **4l** in DMSO-d_6

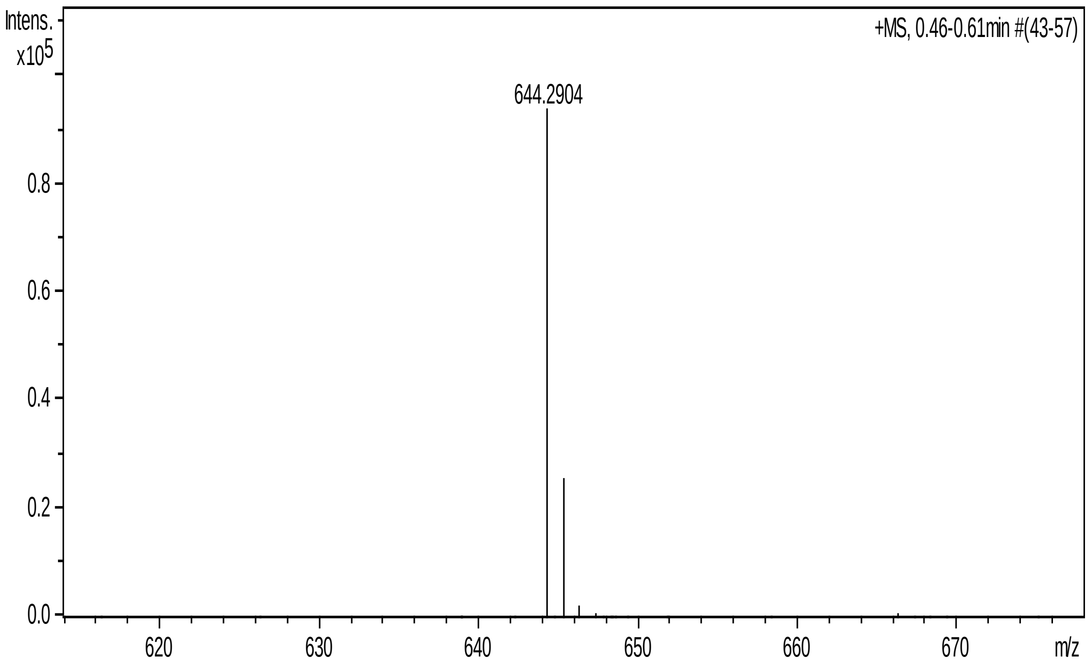

Copy of HRMS of **4l**

1-Benzyl-2'-(4-chlorobenzoyl)-5,5''-dimethyl-6',10b'-dihydro-2'H,5'H-dispiro[indoline-3,1'-pyrrolo[2,1-a]isoquinoline-3',3''-indoline]-2,2''-dione (4m)

white solid, 66%, m.p. 251~252℃; ^{1}H NMR (400 MHz, DMSO-d_6) δ: 10.42 (s, 1H, NH), 7.71 (s, 1H, ArH), 7.31~7.29 (m, 4H, ArH), 7.23~7.22 (m, 2H, ArH), 7.15~7.13 (m, 2H, ArH), 7.09 (d, J = 8.0Hz, 1H, ArH), 7.06~7.01 (m, 4H, ArH), 6.88 (d, J = 8.0Hz, 1H, ArH), 6.79 (d, J = 7.6Hz, 1H, ArH), 6.66~6.63 (m, 1H, ArH), 6.51 (d, J = 8.0Hz, 1H, ArH), 6.01 (d, J = 7.6Hz, 1H, ArH), 5.67 (s, 1H, CH), 4.94 (d, J = 14.0Hz, 2H, CH), 4.16 (d, J = 15.2Hz, 1H, CH), 2.88~2.85 (m, 2H, CH), 2.67~2.62 (m, 2H, CH), 2.31 (s, 3H, CH_3), 2.22 (s, 3H, CH_3); ^{13}C NMR (150 MHz, DMSO-d_6) δ: 195.9, 177.4, 177.2, 141.1, 140.0, 137.3, 135.8, 135.3, 134.5, 134.4, 131.7, 130.4, 130.0, 129.2, 128.9, 128.7, 128.4, 128.1, 128.0, 127.4, 126.8, 126.3, 126.1, 125.5, 125.1, 123.3, 109.2, 108.1, 70.6, 68.4, 66.6, 57.6, 43.9, 41.5, 29.1, 20.8, 20.6; IR(KBr) υ: 3435, 3272, 3031, 2918, 2817, 1710, 1623, 1588, 1494, 1431, 1402, 1361, 1305, 1203, 1169, 1089, 1006, 939, 858, 811, 730, 700cm^{-1}; MS (m/z): HRMS (ESI) Calcd. for $C_{42}H_{35}ClN_3O_3$ ([M+H]$^+$): 664.2361, found: 664.2357.

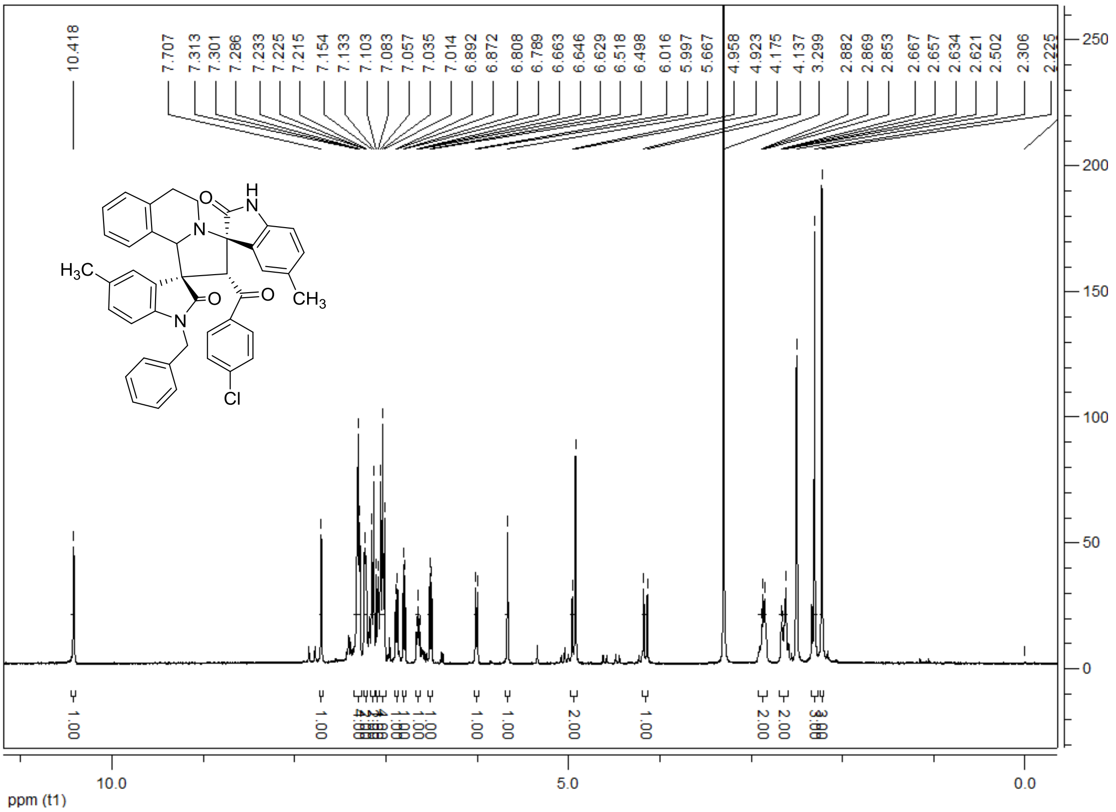

Copy of ^{1}H NMR spectrum of **4m** in DMSO-d_6

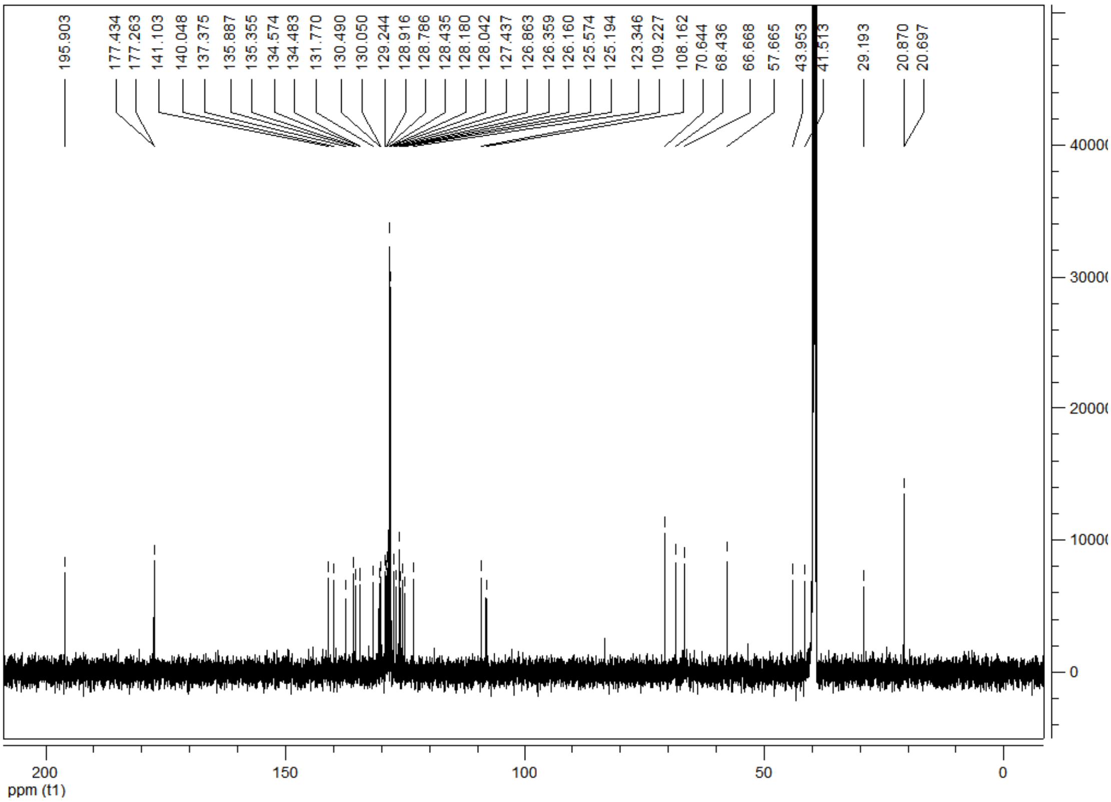

Copy of ^{13}C NMR spectrum of **4m** in DMSO-d_6

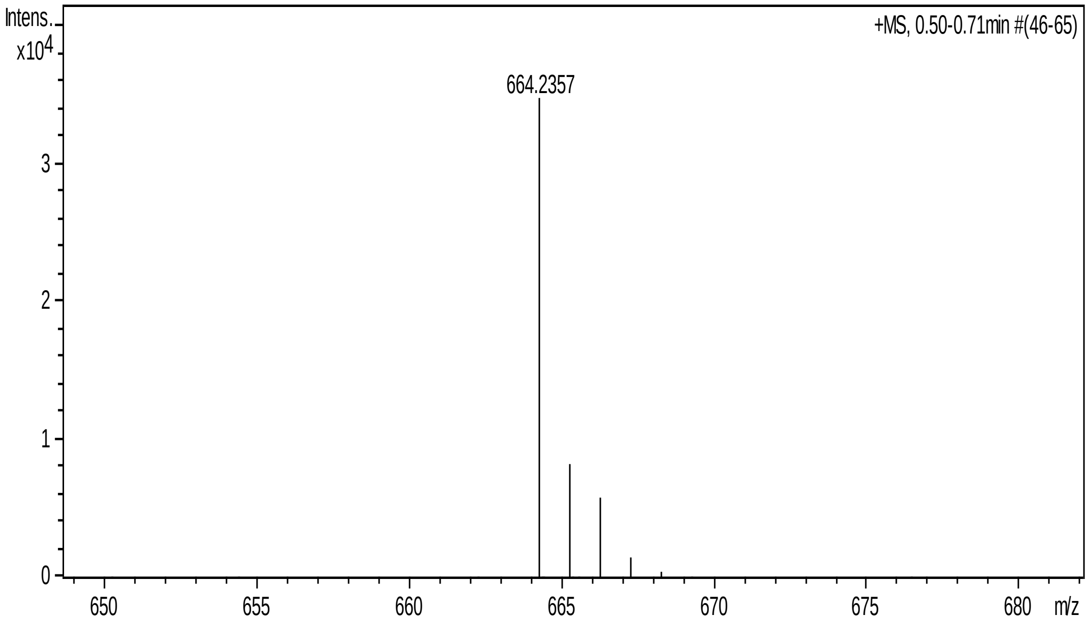

Copy of HRMS of **4m**

1-Butyl-5,5''-dimethyl-2'-(4-methylbenzoyl)-6',10b'-dihydro-2'H,5'H-dispiro[indoline-3,1'-pyrrolo[2,1-a]isoquinoline-3',3''-indoline]-2,2''-dione (4n)

white solid, 90%, m.p. 243~244℃; ^{1}H NMR (400 MHz, DMSO-d_6) δ: 10.36 (s, 1H, NH), 7.69 (brs, 1H, ArH), 7.23 (brs, 1H, ArH), 7.08~7.04 (m, 3H, ArH), 6.98~6.92 (m, 4H, ArH), 6.90 (d, J = 7.6Hz, 1H, ArH), 6.82~6.77 (m, 2H, ArH), 6.51 (d, J = 8.0Hz, 1H, ArH), 6.09 (d, J = 7.6Hz, 1H, ArH), 5.61 (s, 1H, CH), 4.89 (s, 1H, CH), 3.63~3.56 (m, 1H, CH), 3.10~3.04 (m, 1H, CH), 2.89~2.80 (m, 2H, CH), 2.65~2.57 (m, 2H, CH), 2.29 (s, 3H, CH_3), 2.23 (s, 3H, CH_3), 2.20 (s, 3H, CH_3), 1.46~1.38 (m, 1H, CH), 1.30~1.24 (m, 1H, CH), 1.22~1.16 (m, 2H, CH), 0.86~0.83 (t, J = 7.2Hz, 3H, CH_3); ^{13}C NMR (100 MHz, DMSO-d_6) δ: 196.6, 177.8, 177.5, 143.1, 141.5, 140.7, 135.4, 134.9, 134.6, 131.5, 130.8, 130.4, 129.8, 129.3, 129.1, 128.9, 127.5, 127.2, 126.7, 126.6, 125.6, 125.3, 123.6, 109.6, 108.1, 70.9, 68.9, 66.8, 58.1, 41.9, 29.8, 21.4, 21.3, 21.1, 20.3, 14.1; IR(KBr) υ: 3437, 3263, 3058, 2958, 2925, 2863, 2828, 1708, 1626, 1605, 1494, 1410, 1368, 1308, 1203, 1170, 1113, 1011, 808, 747, 724cm^{-1}; MS (m/z): HRMS (ESI) Calcd. for $C_{40}H_{40}N_3O_3$ ($[M+H]^+$): 610.3064, found: 610.3059.

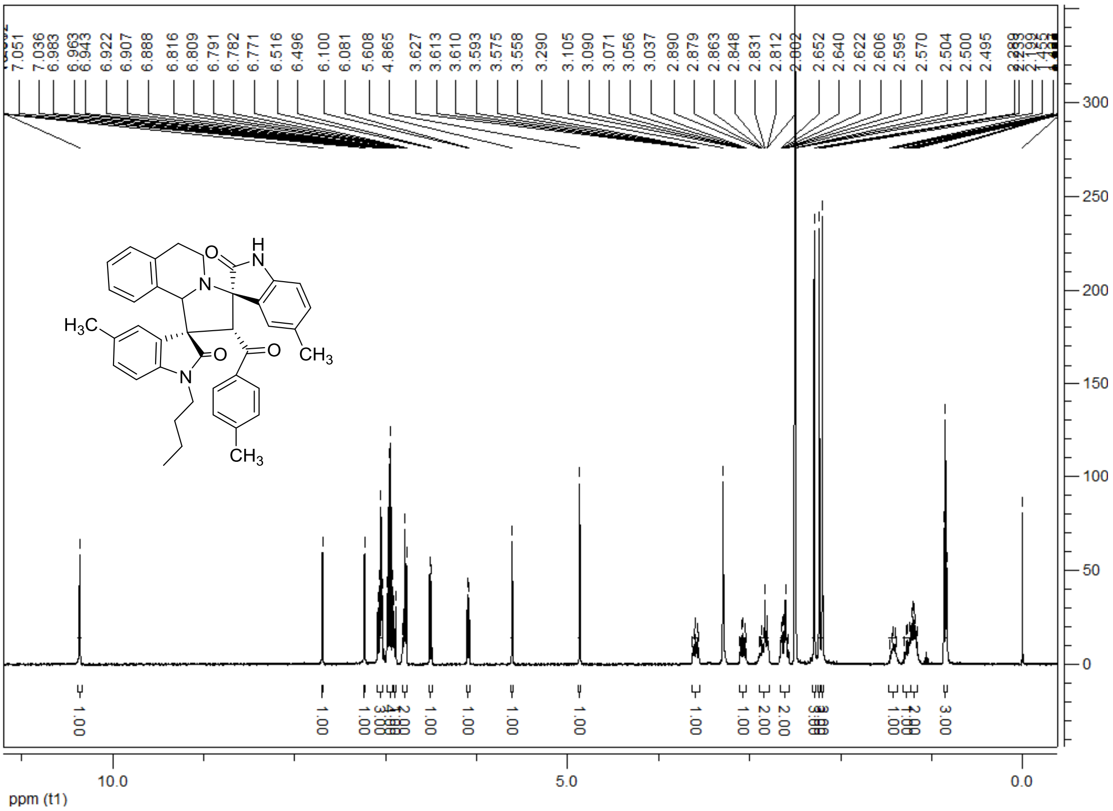

Copy of ^{1}H NMR spectrum of **4n** in DMSO-d_6

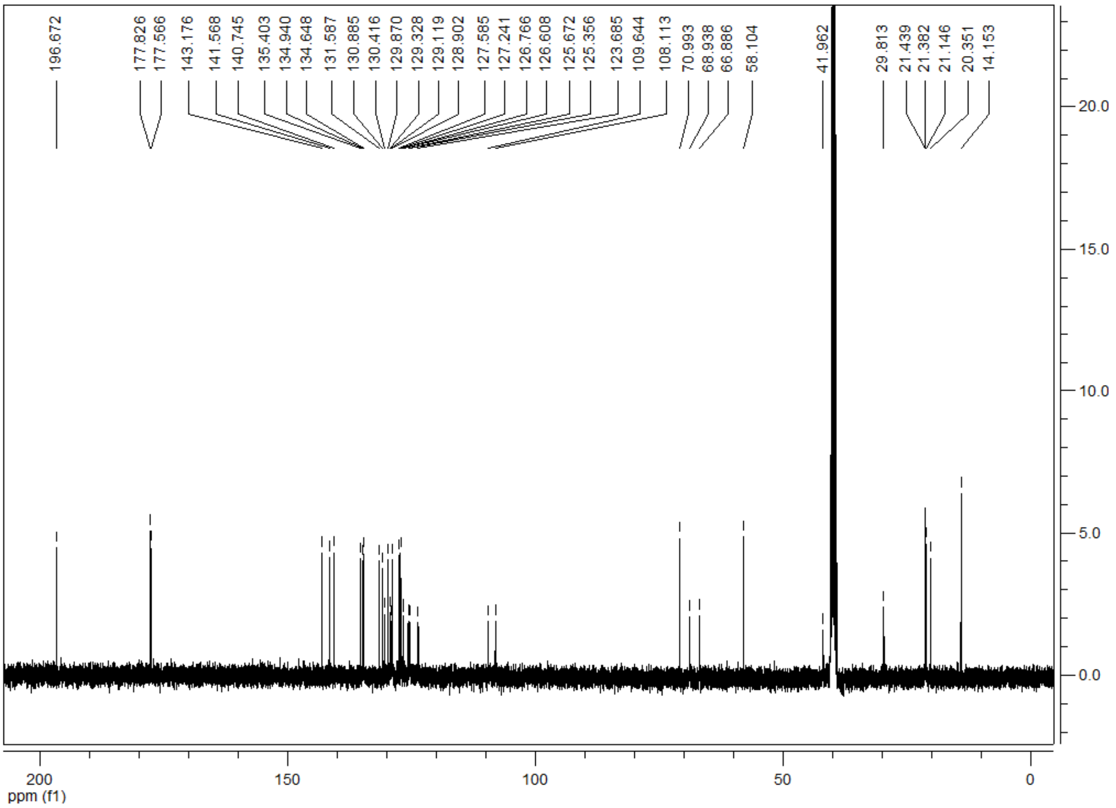

Copy of ^{13}C NMR spectrum of **4n** in DMSO-d_6

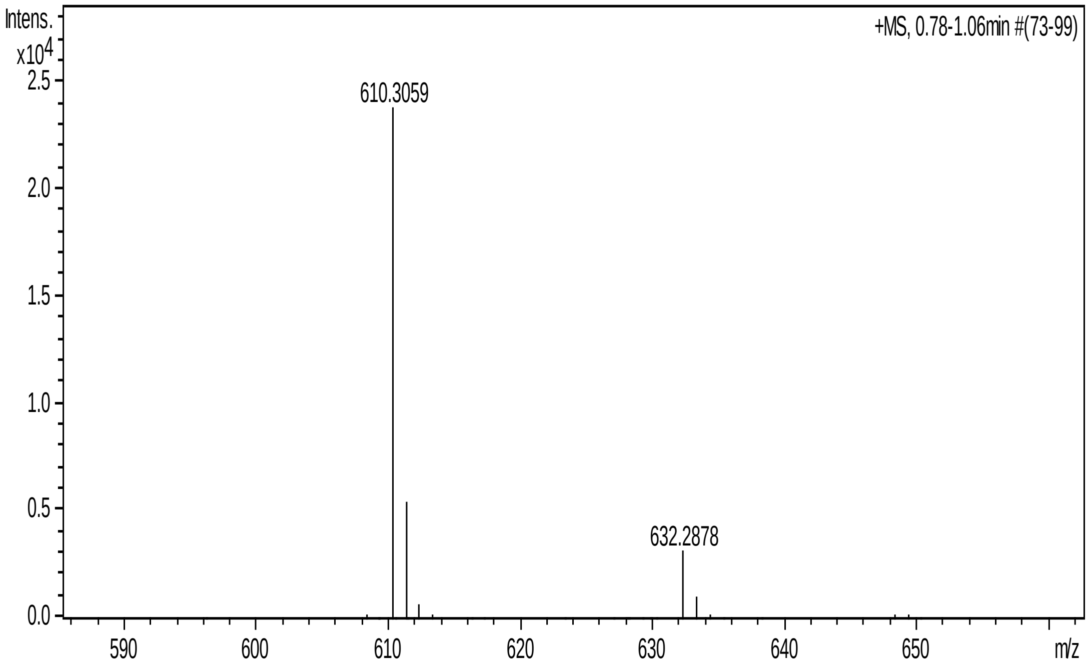

Copy of HRMS of **4n**

1-Butyl-5,5''-dichloro-2'-(4-methylbenzoyl)-6',10b'-dihydro-2'H,5'H-dispiro[indoline-3,1'-pyrrolo[2,1-a]isoquinoline-3',3''-indoline]-2,2''-dione (4o)

white solid, 72%, m.p. 242~243℃; ^{1}H NMR (400 MHz, DMSO-d_6) δ: 10.72 (s, 1H, NH), 7.90~7.89 (m, 1H, ArH), 7.46 (m, 1H, ArH), 7.35 (dd, J_1= 8.2Hz, J_2= 2.0Hz, 1H, ArH), 7.16 (dd, J_1= 8.4Hz, J_2= 2.0Hz, 1H, ArH), 7.07~7.06 (m, 2H, ArH), 7.00 (brs, 4H, ArH), 6.93 (d, J = 8.4Hz, 1H, ArH), 6.85~6.81 (m, 1H, ArH), 6.67 (d, J = 8.4Hz, 1H, ArH), 6.07 (d, J = 8.0Hz, 1H, ArH), 5.56 (s, 1H, CH), 4.94 (s, 1H, CH), 3.64~3.58 (m, 1H, CH), 3.11~3.06 (m, 1H, CH), 2.88~2.83 (m, 2H, CH), 2.67~2.60 (m, 2H, CH), 2.22 (s, 3H, CH_3), 1.44~1.39 (m, 1H, CH), 1.27~1.17 (m, 3H, CH), 0.845 (t, J = 7.2Hz, 3H, CH_3); ^{13}C NMR (100 MHz, DMSO-d_6) δ: 196.7, 177.6, 177.0, 143.5, 142.8, 142.0, 134.9, 134.7, 134.4, 131.7, 130.2, 129.4, 129.1, 128.9, 127.3, 127.0, 126.9, 126.0, 125.8, 125.8, 125.3, 123.5, 111.5, 110.0, 71.2, 68.9, 66.8, 58.1, 42.0, 29.6, 29.5, 21.4, 20.2, 14.0; IR(KBr) υ: 3434, 3185, 3070, 2930, 2867, 1713, 1674, 1610, 1479, 1435, 1350, 1285, 1249, 1223, 1183, 1142, 1115, 1078, 947, 895, 811, 787, 732cm^{-1}; MS (m/z): HRMS (ESI) Calcd. for $C_{38}H_{33}Cl_2N_3O_3$ ($[M+H]^+$): 650.1972, found: 650.1974.

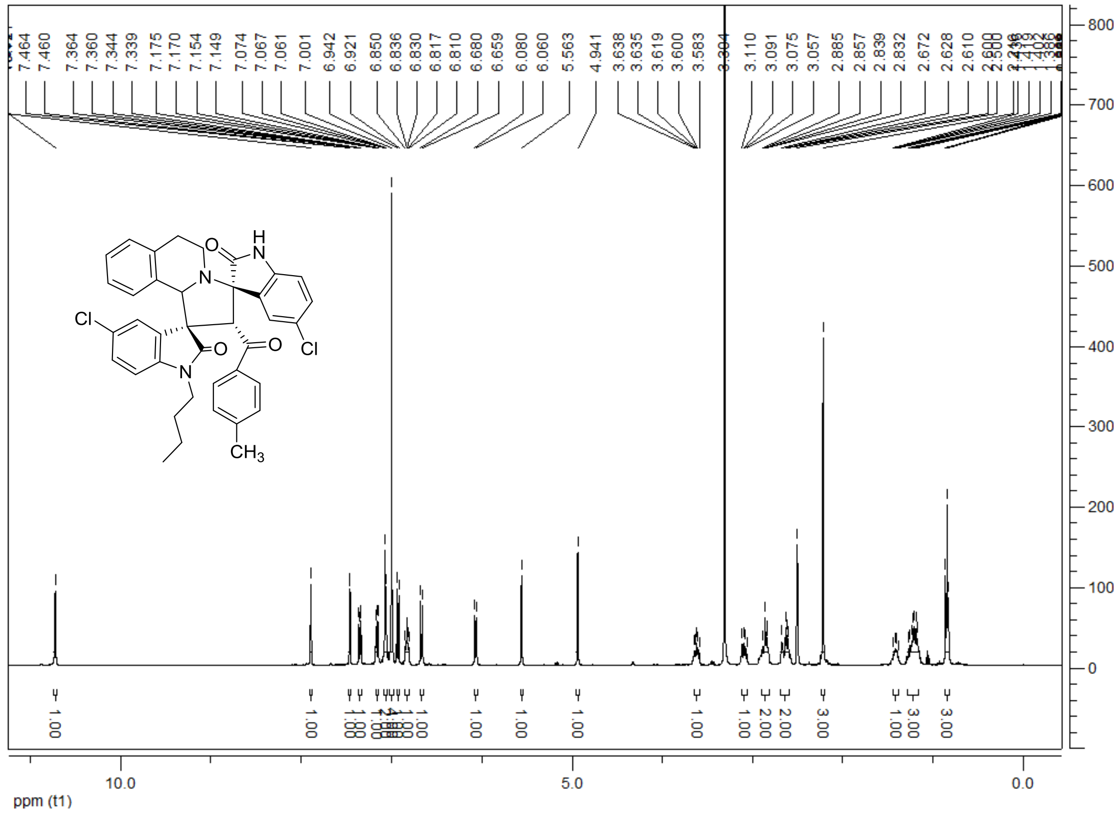

Copy of ^{1}H NMR spectrum of **4o** in DMSO-d_6

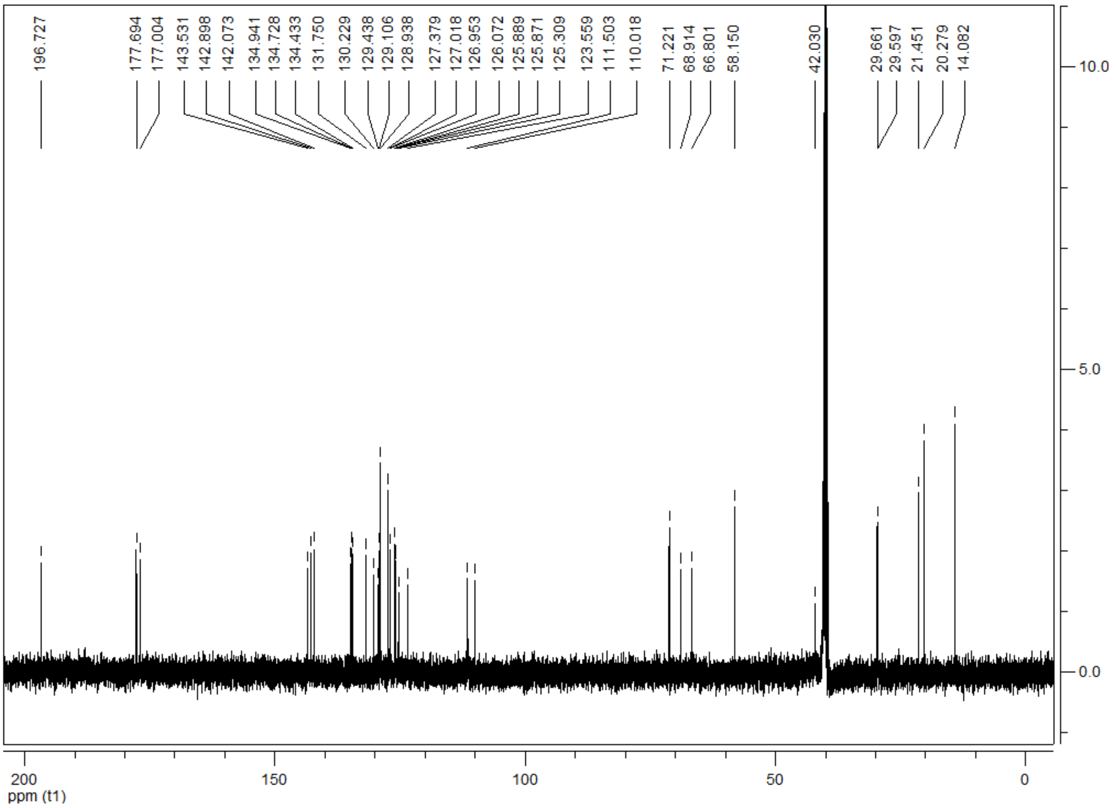

Copy of ^{13}C NMR spectrum of **4o** in DMSO-d_6

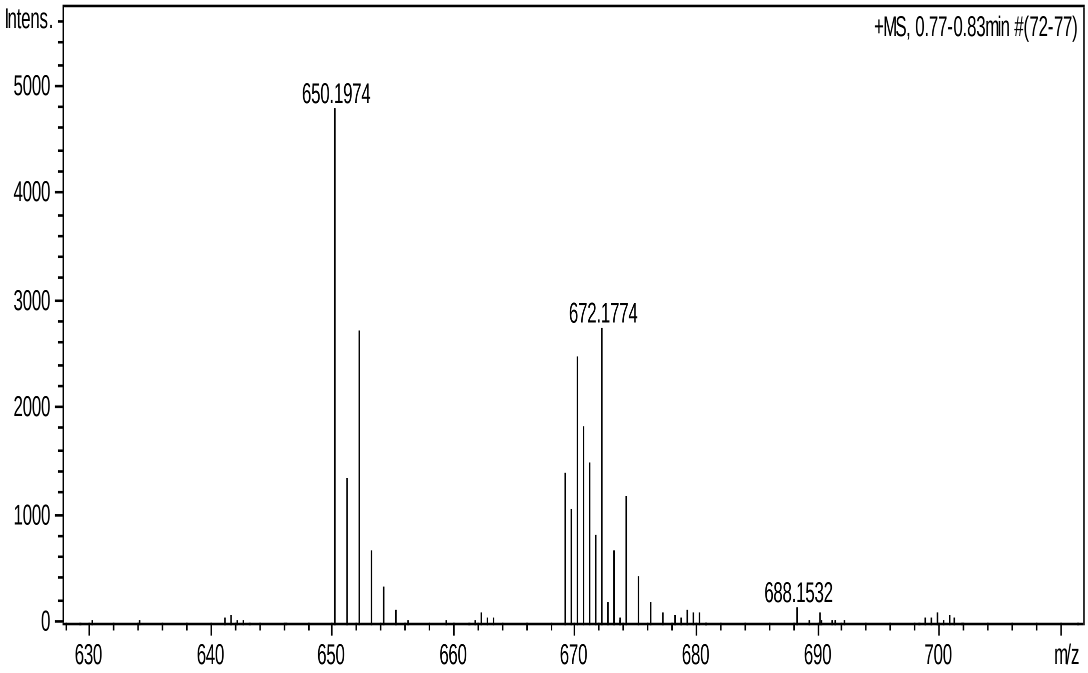

Copy of HRMS of **4o**

1'',5-Dimethyl-2'-(4-methylbenzoyl)-6',10b'-dihydro-2'H,5'H-dispiro[indoline-3,1'-pyrrolo[2,1-a]isoquinoline-3',3''-indoline]-2,2''-dione (4p)

white solid, 85%, m.p. 246~247℃; ^{1}H NMR (400 MHz, DMSO-d_6) δ: 10.40 (s, 1H, NH), 7.63 (s, 1H, ArH), 7.48 (d, J = 7.2Hz, 1H, ArH), 7.40~7.36 (m, 1H, ArH), 7.14~7.10 (m, 2H, ArH), 7.08~7.05 (m, 4H, ArH), 7.00 (d, J = 8.0Hz, 2H, ArH), 6.91~6.86 (m, 1H, ArH), 6.82 (d, J = 8.0Hz, 1H, ArH), 6.37 (d, J = 7.6Hz, 1H, ArH), 6.24 (d, J = 8.0Hz, 1H, ArH), 5.62 (s, 1H, CH), 4.95 (s, 1H, CH), 3.29 (s, 3H, CH_3), 2.87~2.77 (m, 2H, 2CH), 2.63~2.59 (m, 1H, CH), 2.57~2.52 (m, 1H, CH), 2.22 (s, 3H, CH_3), 2.20 (s, 3H, CH_3); ^{13}C NMR (100 MHz, DMSO-d_6) δ: 196.6, 179.4, 176.1, 145.3, 143.3, 139.6, 135.5, 134.9, 134.5, 130.9, 130.5, 130.2, 129.2, 129.1, 129.0, 127.3, 126.8, 126.7, 126.6, 125.9, 124.5, 123.6, 122.7, 109.1, 108.8, 70.9, 69.4, 66.9, 58.8, 41.9, 29.7, 26.4, 21.4, 21.4; IR(KBr) υ: 3424, 3199, 2909, 2821, 1720, 1674, 1607, 1493, 1470, 1415, 1373, 1346, 1291, 1263, 1204, 1182, 1156, 1130, 1039, 976, 945, 810, 752cm^{-1}; MS (m/z): HRMS (ESI) Calcd. for $C_{36}H_{32}N_3O_3$ ([M+H]$^{+}$): 554.2438, found: 554.2456.

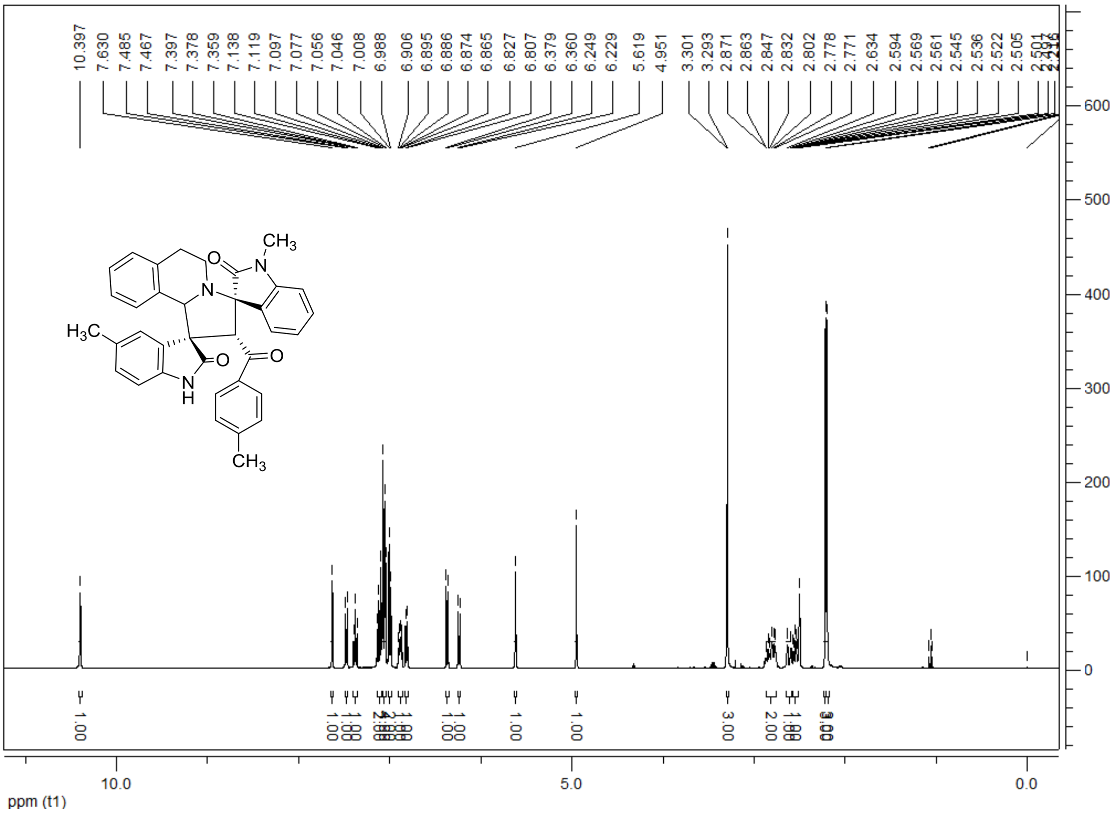

Copy of ^{1}H NMR spectrum of **4p** in DMSO-d_6

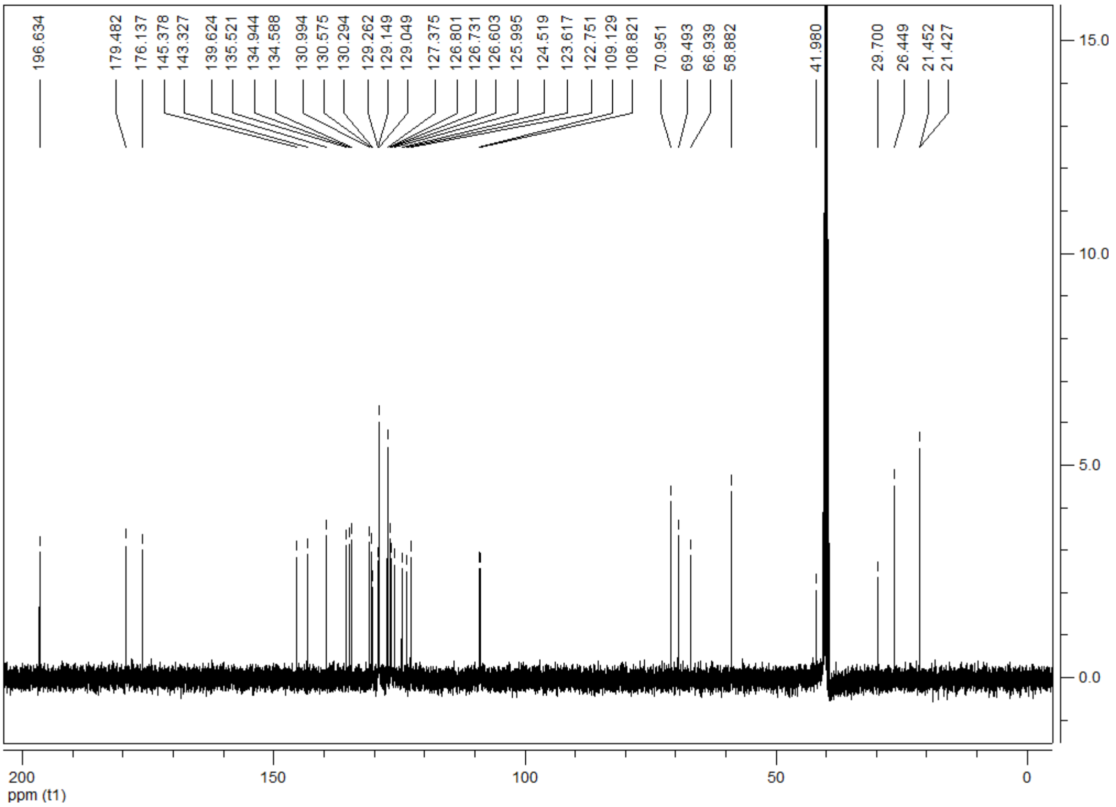

Copy of ^{13}C NMR spectrum of **4p** in DMSO-d_6

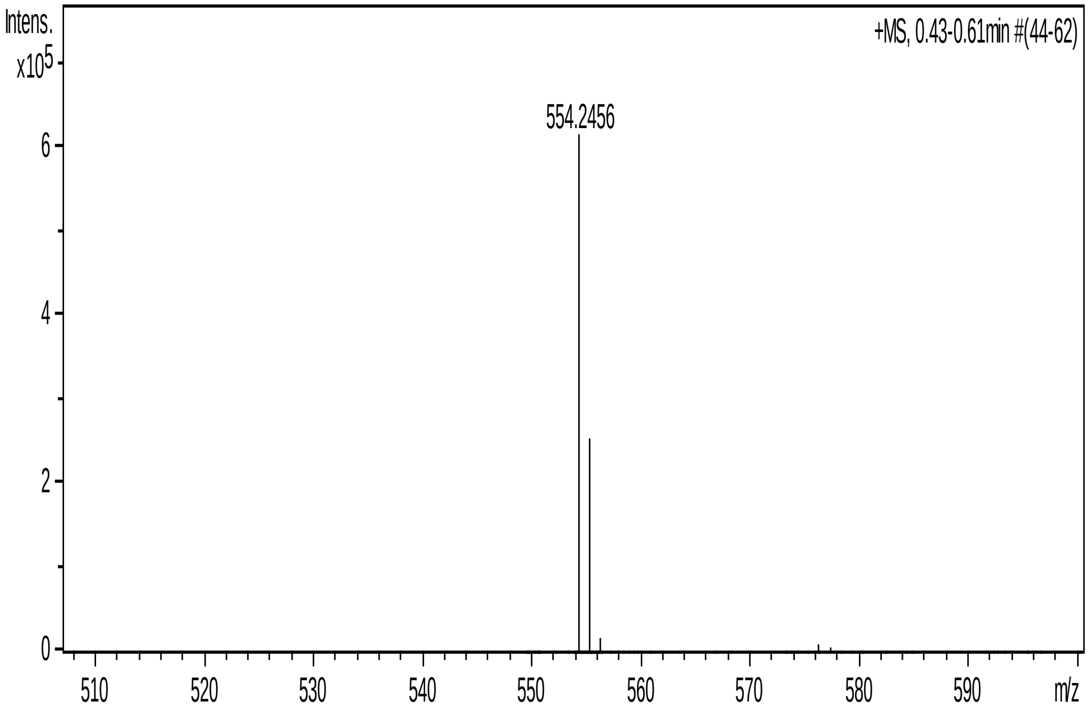

Copy of HRMS of **4p**

5-Chloro-1''-methyl-2'-(4-methylbenzoyl)-6',10b'-dihydro-2'H,5'H-dispiro[indoline-3, 1'-pyrrolo[2,1-a]isoquinoline-3',3''-indoline]-2,2''-dione (4q)

white solid, 63%, m.p. 238~240℃; ^{1}H NMR (600 MHz, DMSO-d_6) δ: 10.68 (s, 1H, NH), 7.88 (brs, 1H, ArH), 7.50 (d, J = 7.2Hz, 1H, ArH), 7.41~7.38 (m, 1H, ArH), 7.14~7.07 (m, 7H, ArH), 7.05~7.04 (m, 2H, ArH), 6.94~6.91 (m, 1H, ArH), 6.49 (d, J = 8.4Hz, 1H, ArH), 6.22 (d, J = 7.8Hz, 1H, ArH), 5.61 (s, 1H, CH), 4.97 (s, 1H, CH), 3.32 (s, 3H, CH_3), 2.86~2.82 (m, 2H, CH), 2.65~2.61 (m, 1H, CH), 2.58~2.54 (m, 1H, CH), 2.22 (s, 3H, CH_3); ^{13}C NMR (150 MHz, DMSO-d_6) δ: 196.0, 178.6, 175.8, 144.8, 143.2, 140.5, 134.5, 134.5, 133.8, 132.0, 129.9, 128.9, 128.7, 128.4, 126.9, 126.4, 125.8, 125.7, 125.6, 124.2, 123.0, 122.3, 110.4, 108.5, 70.6, 69.1, 66.2, 58.7, 41.5, 29.1, 26.0, 20.9; IR(KBr) υ: 3175, 3054, 2930, 2832, 1711, 1681, 1613, 1473, 1444, 1374, 1335, 1278, 1229, 1182, 1128, 1108, 1040, 942, 901, 769, 743cm^{-1}; MS (m/z): HRMS (ESI) Calcd. for $C_{35}H_{29}ClN_3O_3$ ([M+H]$^+$): 574.1892, found: 574.1893.

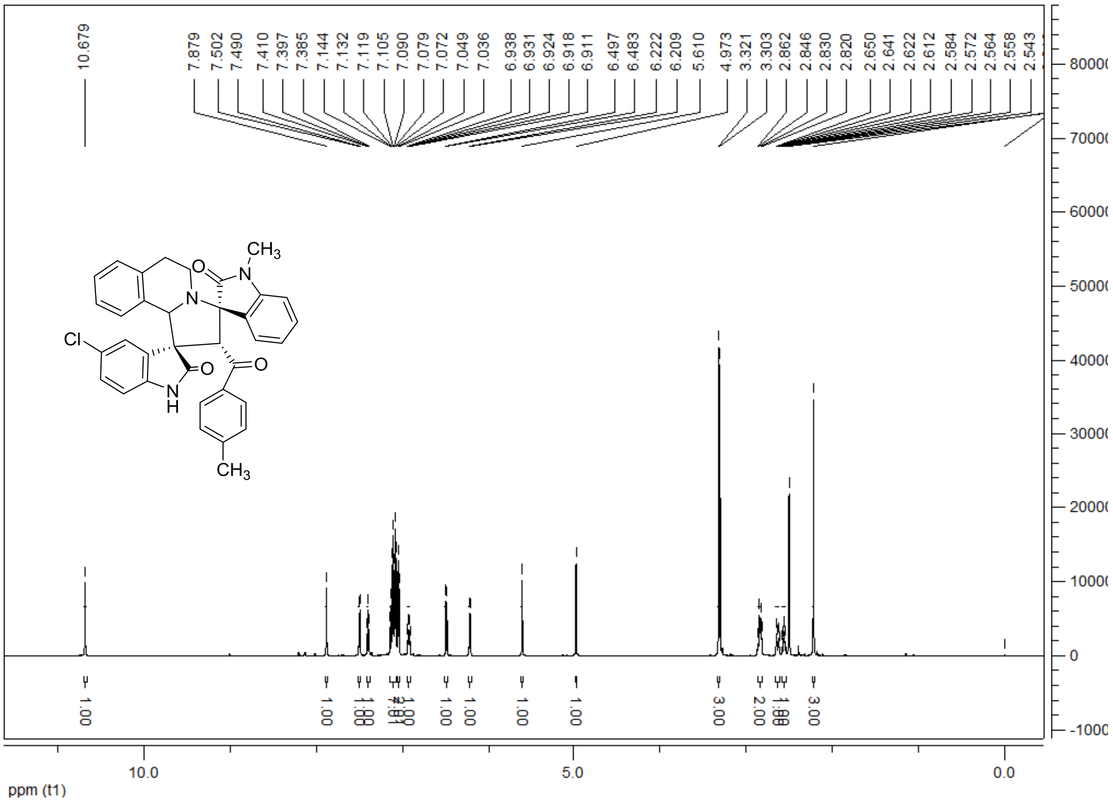

Copy of ^{1}H NMR spectrum of **4q** in DMSO-d_6

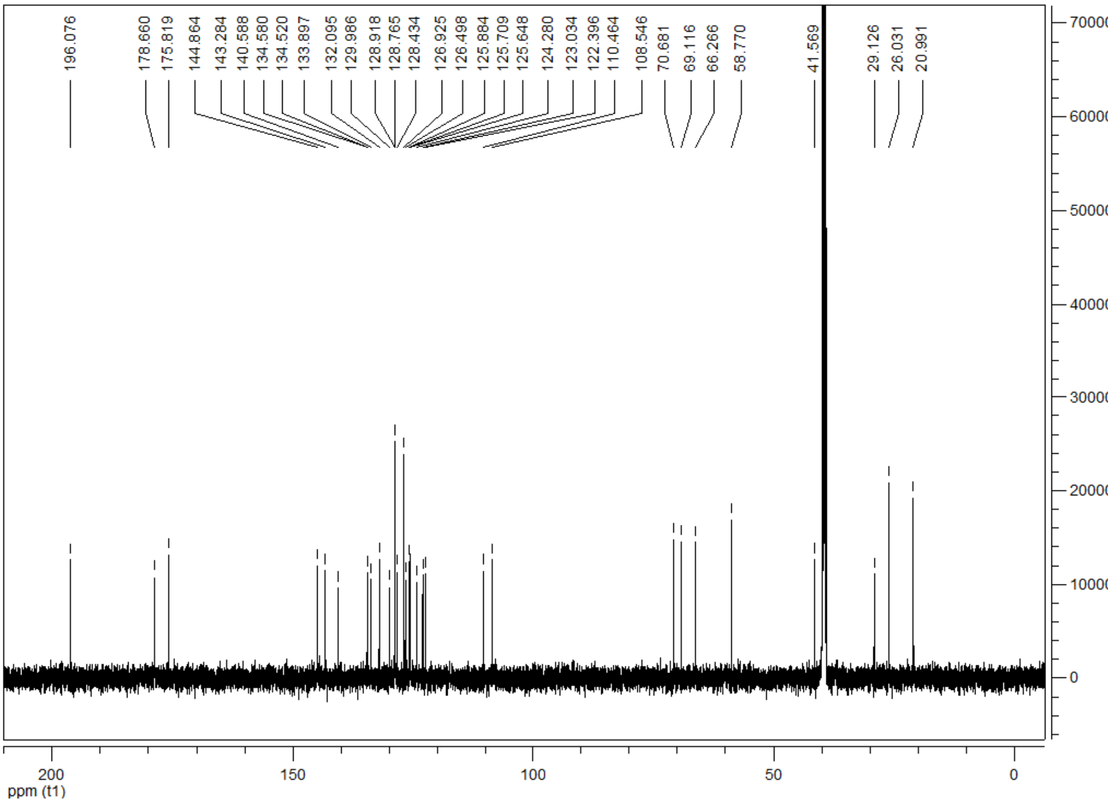

Copy of ^{13}C NMR spectrum of **4q** in DMSO-d_6

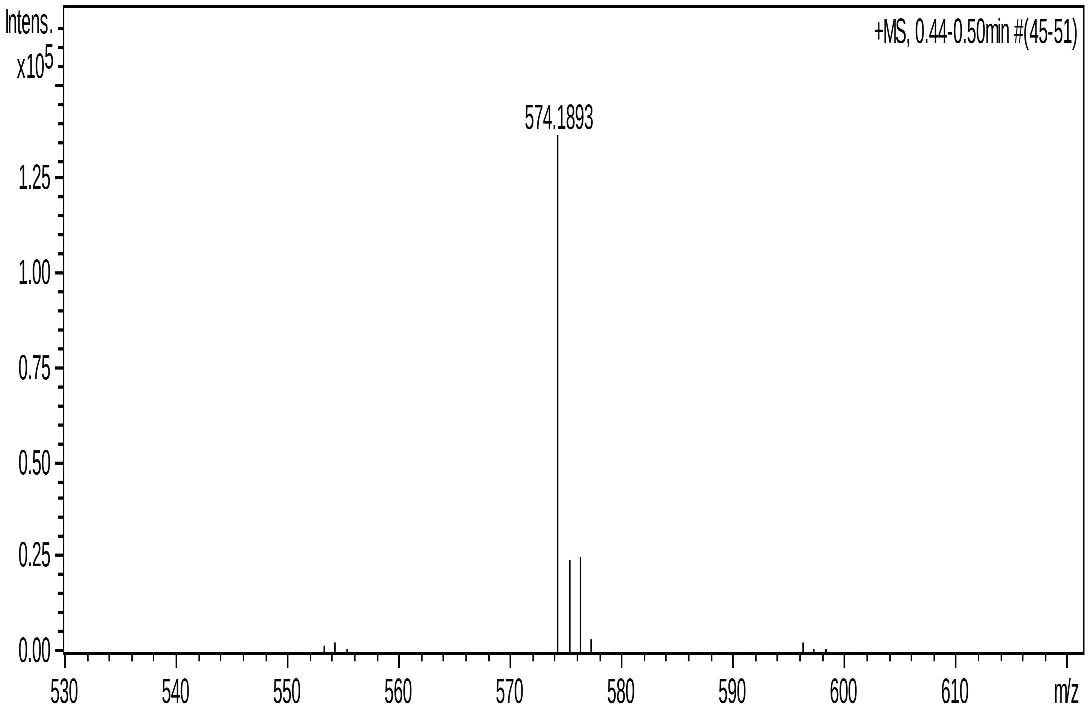

Copy of HRMS of **4q**

1-Benzyl-2'-(4-chlorobenzoyl)-1'',5-dimethyl-6',10b'-dihydro-2'H,5'H-dispiro[indoline-3,1'-pyrrolo[2,1-a]isoquinoline-3',3''-indoline]-2,2''-dione (4r)

white solid, 58%, m.p. 178~180℃; ^{1}H NMR (600 MHz, DMSO-d_6) δ: 7.71 (brs, 1H, ArH), 7.53~7.52 (m, 1H, ArH), 7.41~7.40 (m, 1H, ArH), 7.31 (brs, 3H, ArH), 7.22 (brs, 2H, ArH), 7.14~7.02 (m, 8H, ArH), 6.90~6.89 (m, 1H, ArH), 6.66 (m, 1H, ArH), 6.53~6.52 (m, 1H, ArH), 6.02~6.00 (m, 1H, ArH), 5.70 (s, 1H, CH), 4.99 (s, 1H, CH), 4.94 (d, J = 15.0Hz, 1H, CH), 4.17 (d, J = 15.6Hz, 1H, CH), 3.32 (s, 3H, CH_3), 2.86~2.85 (m, 2H, CH), 2.66~2.56 (m, 2H, CH), 2.24 (s, 3H, CH_3); ^{13}C NMR (150 MHz, DMSO-d_6) δ: 195.8, 177.3, 175.5, 144.8, 140.0, 137.5, 135.8, 135.1, 134.4, 134.3, 131.8, 129.9, 129.0, 128.9, 128.8, 128.4, 128.1, 128.0, 127.4, 126.4, 126.0, 125.9, 125.6, 124.3, 123.3, 122.3, 108.4, 108.2, 70.4, 68.7, 66.9, 57.8, 43.9, 41.5, 29.1, 26.0, 20.9; IR(KBr) υ: 3436, 3030, 2922, 2822, 1712, 1612, 1495, 1472, 1427, 1346, 1262, 1194, 1129, 1089, 1035, 1005, 939, 853, 821, 747, 701cm^{-1}; MS (m/z): HRMS (ESI) Calcd. for $C_{42}H_{35}ClN_3O_3$ ($[M+H]^+$): 664.2361, found: 664.2361.

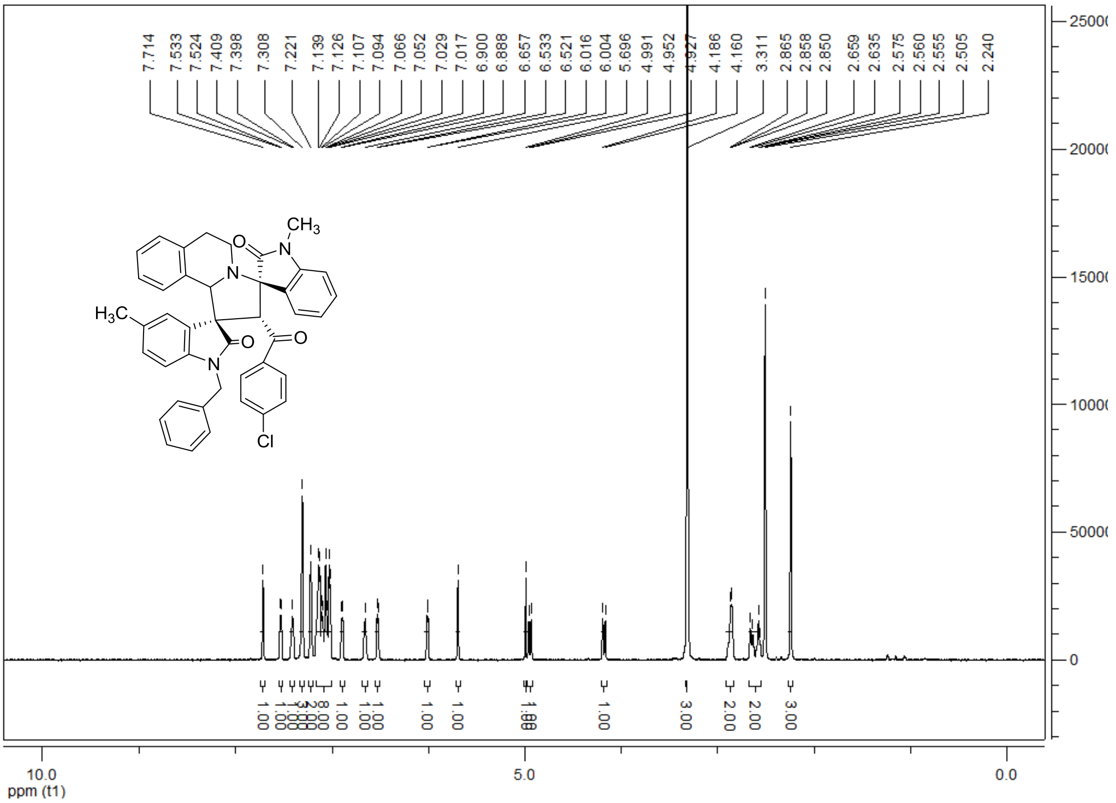

Copy of ^{1}H NMR spectrum of **4r** in DMSO-d_6

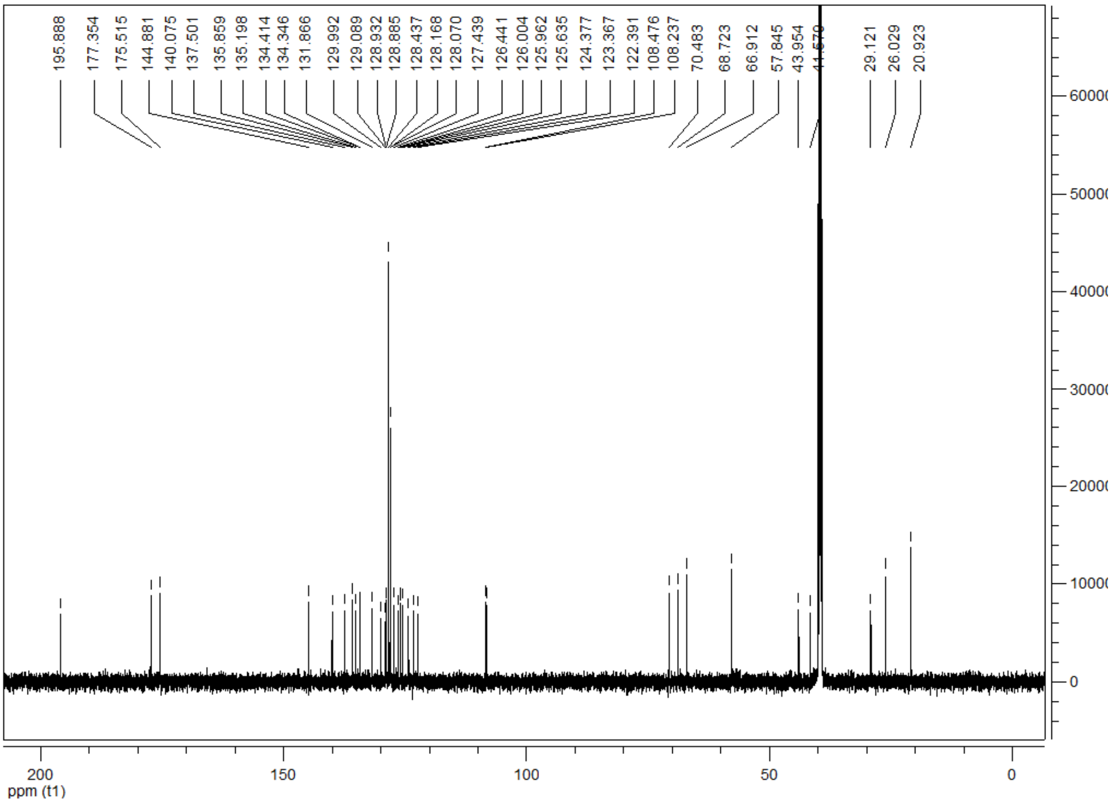

Copy of ^{13}C NMR spectrum of **4r** in DMSO-d_6

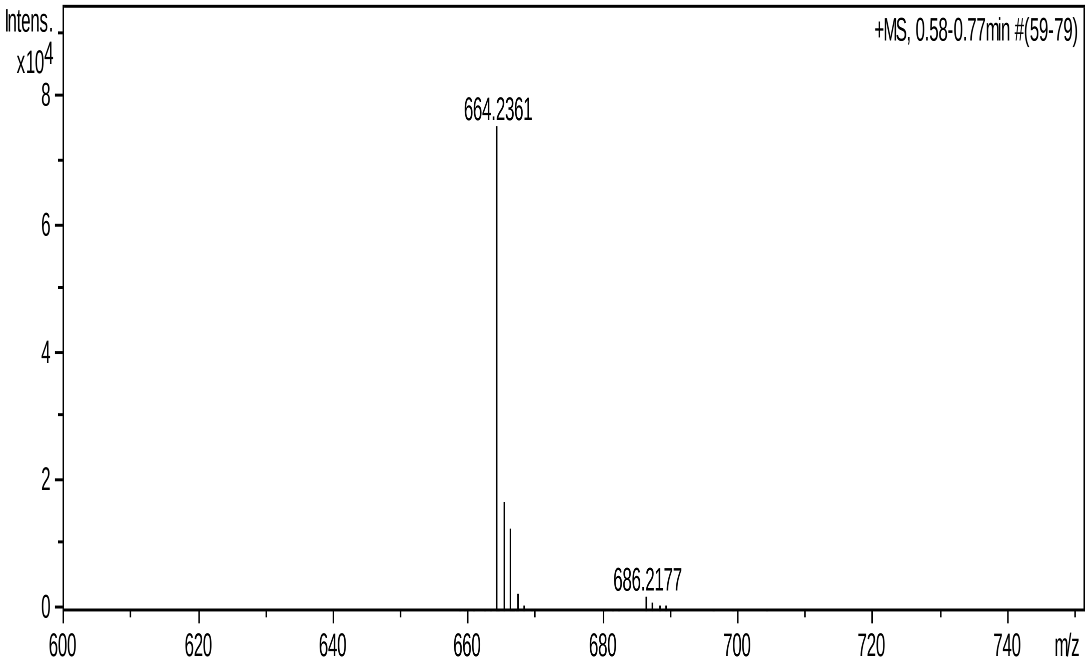

Copy of HRMS of **4r**

1''-Benzyl-5,5''-dimethyl-2'-(4-methylbenzoyl)-6',10b'-dihydro-2'H,5'H-dispiro[indoline-3,1'-pyrrolo[2,1-a]isoquinoline-3',3''-indoline]-2,2''-dione (4s)

white solid, 79%, m.p. 198~200℃; ^{1}H NMR (400 MHz, DMSO-d_6) δ: 10.43 (s, 1H, NH), 7.64 (s, 1H, ArH), 7.53 (d, J = 7.6Hz, 2H, ArH), 7.38~7.34 (m, 2H, ArH), 7.31 (brs, 1H, ArH), 7.29~7.26 (m, 1H, ArH), 7.12 (d, J = 8.4Hz, 2H, ArH), 7.07~7.05 (m, 3H, ArH), 7.02 (d, J = 8.0Hz, 2H, ArH), 6.91~6.87 (m, 1H, ArH), 6.82 (d, J = 7.6Hz, 1H, ArH), 6.72 (d, J = 8.0Hz, 1H, ArH), 6.38 (d, J = 7.6Hz, 1H, ArH), 6.25 (d, J = 7.6Hz, 1H, ArH), 5.62 (s, 1H, CH), 5.11 (d, J = 16.4Hz, 1H, CH), 5.00 (d, J = 15.6Hz, 2H, CH), 2.88~2.83 (m, 1H, CH), 2.73~2.70 (m, 1H, CH), 2.63~2.56 (m, 2H, CH), 2.29 (s, 3H, CH_3), 2.22 (s, 6H, CH_3); ^{13}C NMR (100 MHz, DMSO-d_6) δ: 196.7, 179.4, 176.0, 143.3, 142.0, 139.6, 137.1, 135.6, 134.9, 134.6, 131.9, 131.0, 130.6, 130.3, 129.2, 129.1, 129.0, 128.9, 127.7, 127.6, 127.4, 126.9, 126.7, 126.5, 126.0, 125.1, 123.5, 109.3, 109.1, 70.8, 69.4, 66.8, 58.5, 56.4, 43.2, 41.9, 29.8, 21.4, 21.0; IR(KBr) υ: 3200, 3027, 2972, 2915, 2833, 1705, 1679, 1605, 1497, 1431, 1341, 1212, 1163, 1125, 1048, 878, 813, 754, 705cm^{-1}; MS (m/z): HRMS (ESI) Calcd. for $C_{43}H_{38}N_3O_3$ ($[M+H]^+$): 644.2908, found: 644.2916.

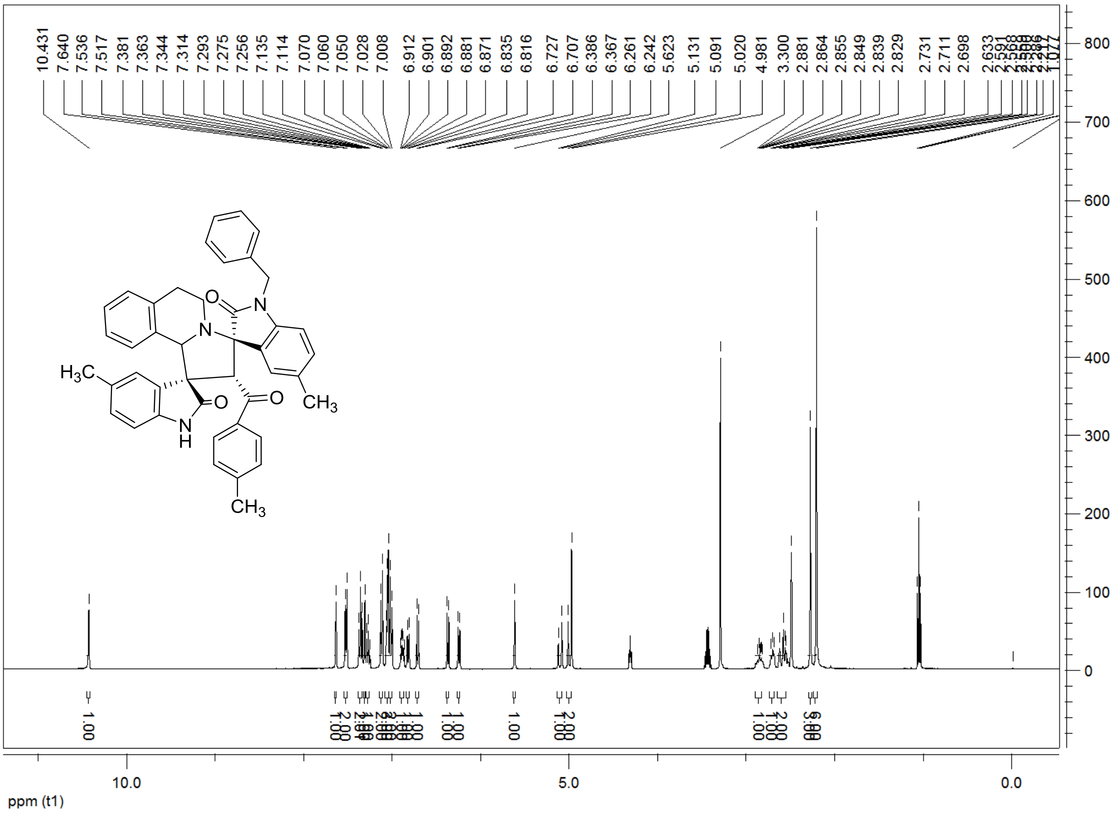

Copy of ^{1}H NMR spectrum of **4s** in DMSO-d_6

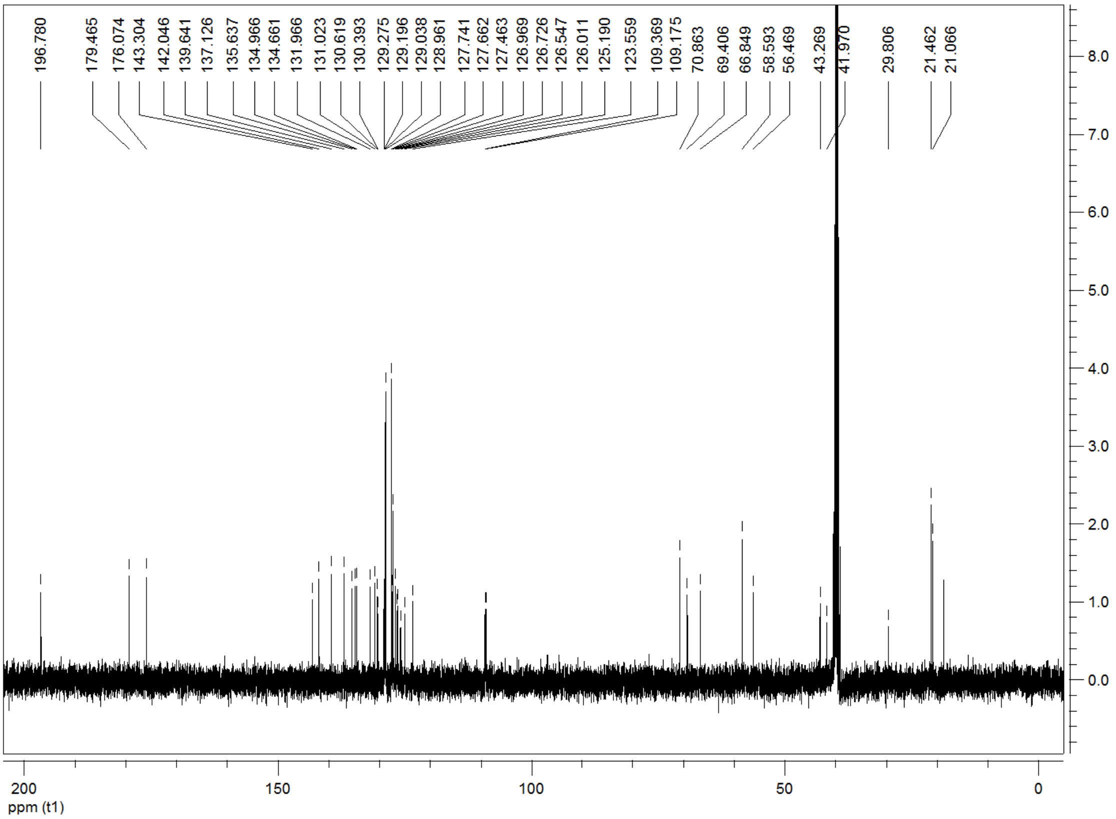

Copy of ^{13}C NMR spectrum of **4s** in DMSO-d_6

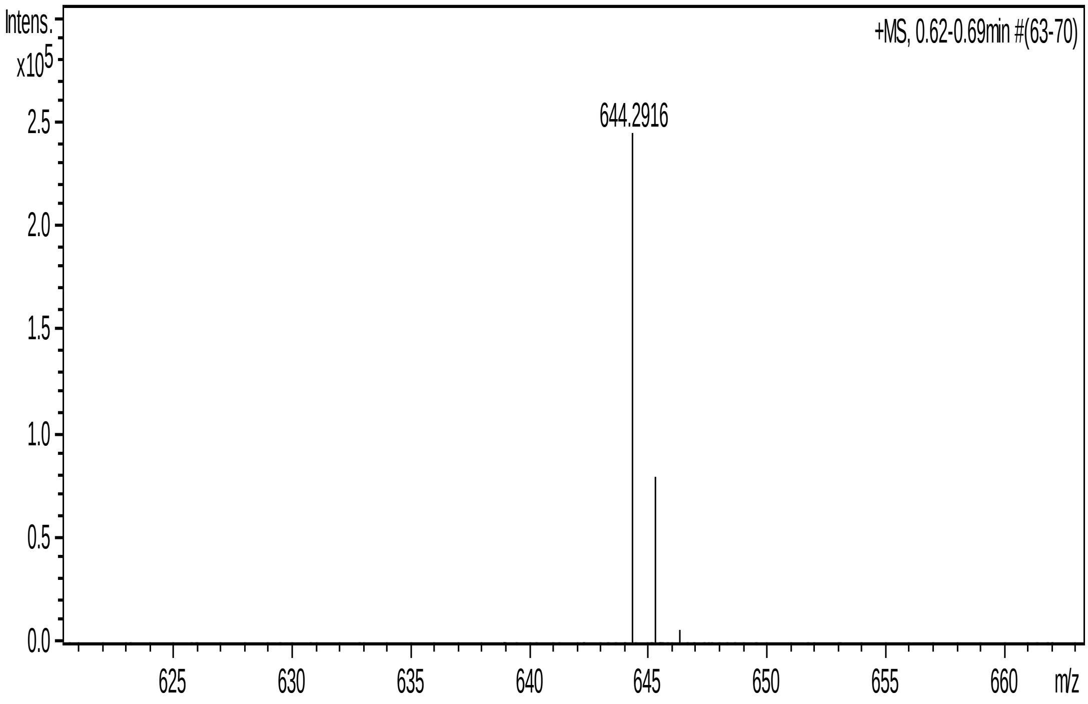

Copy of HRMS of **4s**

1''-Benzyl-5-chloro-2'-(4-methoxybenzoyl)-5''-methyl-6',10b'-dihydro-2'H,5'H-dispiro [indoline-3,1'-pyrrolo[2,1-a]isoquinoline-3',3''-indoline]-2,2''-dione (4t)

white solid, 80%, m.p. 198~199℃; ^{1}H NMR (400 MHz, DMSO-d_6) δ: **major isomer**: 10.71 (s, 1H, NH), 7.89 (brs, 1H, ArH), 7.52 (d, J = 7.6Hz, 2H, ArH), 7.38~7.25 (m, 6H, ArH), 7.11~7.07 (m, 4H, ArH), 6.96~6.92 (m, 1H, ArH), 6.78 (d, J = 8.4Hz, 2H, ArH), 6.73 (d, J = 8.0Hz, 1H, ArH), 6.54 (d, J = 8.4Hz, 1H, ArH), 6.24 (d, J = 7.6Hz, 1H, ArH), 5.63 (s, 1H, CH), 5.11 (d, J = 16.4Hz, 1H, CH), 5.01 (d, J = 17.2Hz, 2H, CH), 3.73 (s, 3H, OCH_3), 2.89~2.74 (m, 2H, CH), 2.65~2.56 (m, 2H, CH), 2.29 (s, 3H, CH_3); **minor isomer:** 10.98 (s, 1H, NH), 7.04 (brs, 1H, ArH), 6.65 (d, J = 9.2Hz, 1H, ArH), 6.60 (d, J = 8.4Hz, 1H, ArH), 5.21 (s, 1H, CH), 3.71 (s, 3H, OCH_3), 2.15 (s, 3H, CH_3). **ratio of major/minor = 90:10**; ^{13}C NMR (100 MHz, DMSO-d_6) δ: 194.5, 178.6, 175.6, 162.7, 141.4, 140.4, 136.4, 134.6, 134.4, 132.0, 131.4, 129.9, 129.1, 129.1, 128.8, 128.4, 128.3, 127.1, 127.1, 126.3, 125.9, 125.7, 125.5, 125.5, 124.7, 122.8, 113.4, 110.4, 108.9, 70.5, 68.9, 65.9, 58.4, 55.3, 42.7, 41.4, 29.1, 20.4; IR(KBr) υ: 3189, 3025, 2912, 2835, 1707, 1676, 1601, 1498, 1472, 1436, 1341, 1257, 1219, 1171, 1033, 981, 943, 895, 846, 815, 748, 702cm^{-1}; MS (m/z): HRMS (ESI) Calcd. for $C_{42}H_{34}ClN_3O_4$ ($[M+H]^+$): 680.2311, found: 680.2313.

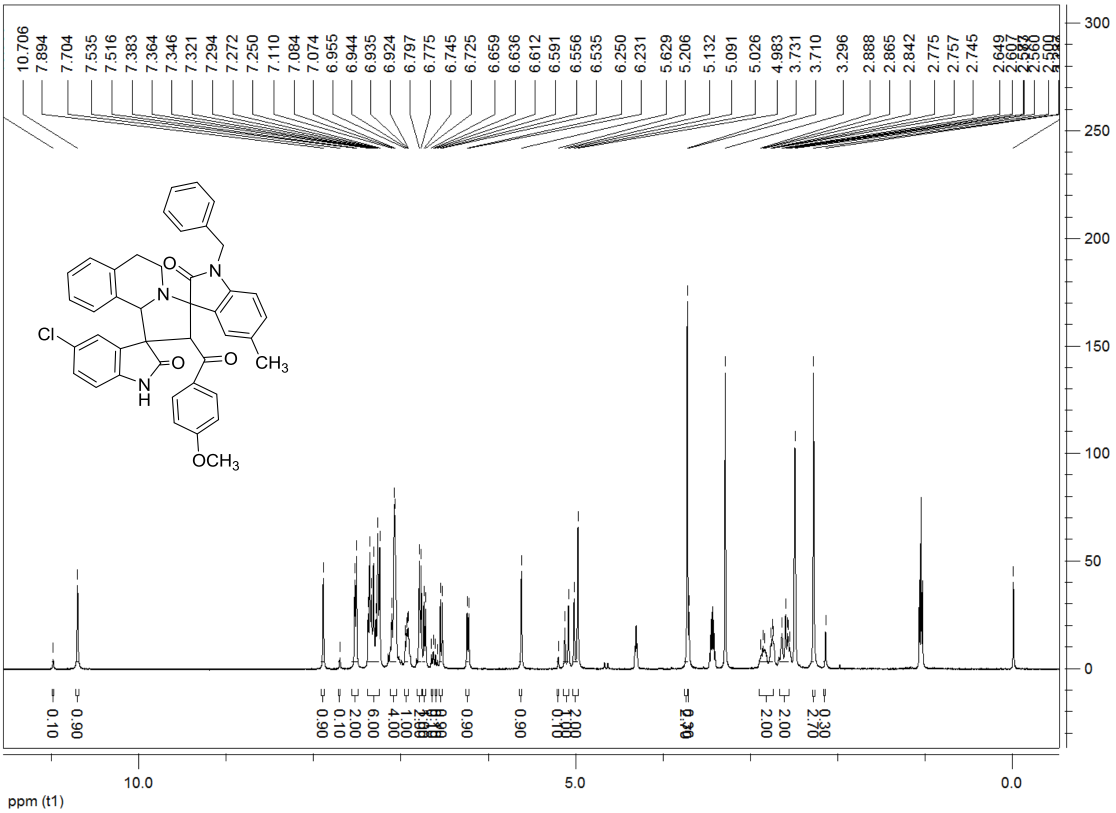

Copy of ^{1}H NMR spectrum of **4t** in DMSO-d_6

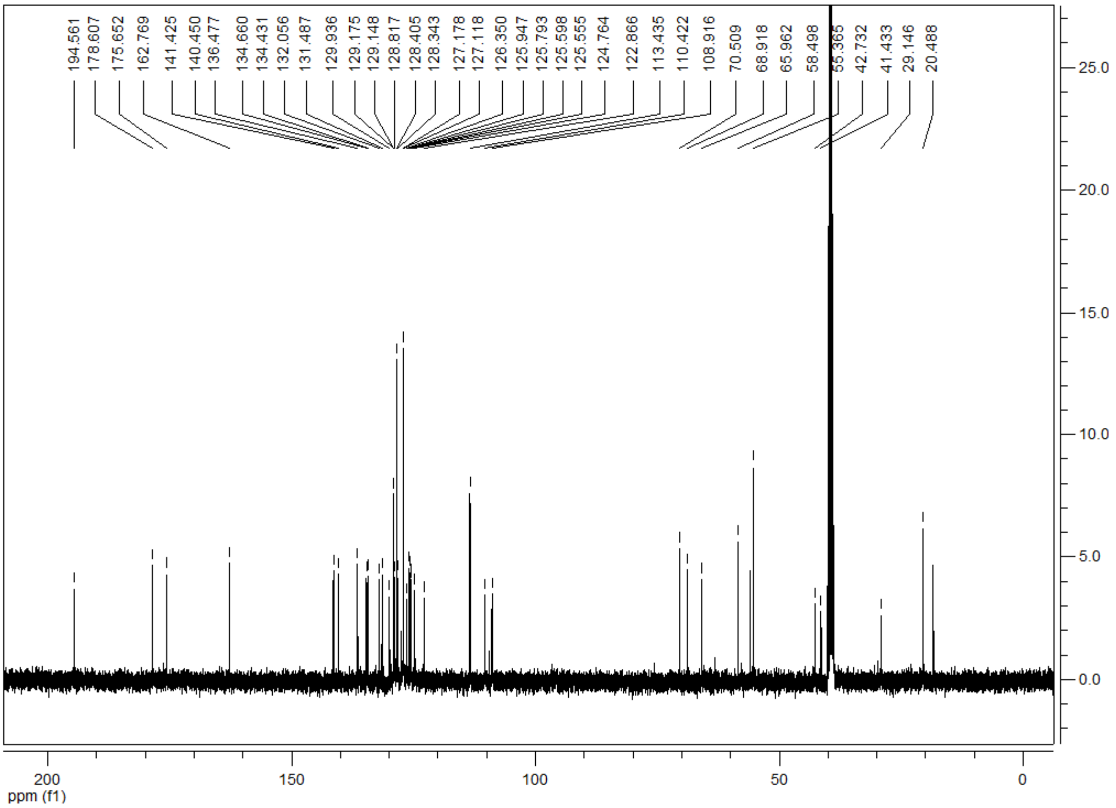

Copy of ^{13}C NMR spectrum of **4t** in DMSO-d_6

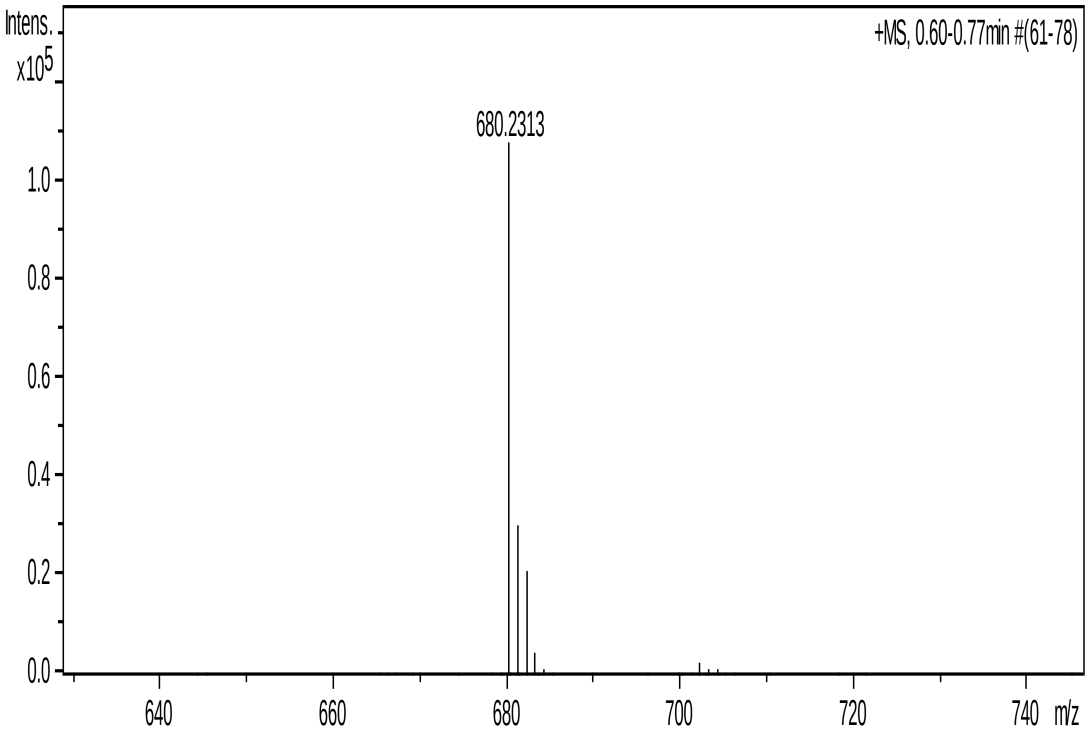

Copy of HRMS of **4t**

1''-Benzyl-5-chloro-5''-methyl-2'-(4-methylbenzoyl)-6',10b'-dihydro-2'H,5'H-dispiro[indoline-3,1'-pyrrolo[2,1-a]isoquinoline-3',3''-indoline]-2,2''-dione (4u)

white solid, 62%, m.p. 178~180℃; ^{1}H NMR (600 MHz, DMSO-d_6) δ: **major isomer**: 10.70 (s, 1H, NH), 7.89 (brs, 1H, ArH), 7.36~7.28 (m, 6H, ArH), 7.16~7.00 (m, 7H, ArH), 6.95~6.87 (m, 2H, ArH), 6.75 (d, *J* = 7.8Hz, 1H, ArH), 6.50 (d, *J* = 8.4Hz, 1H, ArH), 6.23 (d, *J* = 7.2Hz, 1H, ArH), 5.62 (s, 1H, CH), 5.15~5.10 (m, 1H, CH), 5.03~5.01 (m, 2H,CH), 2.89~2.76 (m, 2H,CH), 2.29 (s, 3H, CH_3), 2.24 (s, 3H, CH_3); **minor isomer:** 11.02 (s, 1H, NH), 7.70 (brs, 1H, ArH), 7.54~7.52 (m, 6H, ArH), 6.83 (d, *J* = 7.8Hz, 1H, ArH), 6.62 (d, *J* = 7.2Hz, 1H, ArH), 6.57 (d, *J* = 7.2Hz, 1H, ArH), 5.20 (s, 1H, CH), 4.97 (d, *J* = 15.6Hz, 1H, CH), 4.62 (d, *J* = 15.6Hz, 1H, CH), 2.20 (s, 3H, CH_3), 2.15 (s, 3H, CH_3). **ratio of major/minor = 75:25**; ^{13}C NMR (150 MHz, DMSO-d_6) δ: 196.2, 194.9, 178.6, 177.3, 176.4, 175.7, 143.2, 141.5, 141.4, 140.9, 140.5, 136.5, 135.9, 134.7, 134.6, 134.5, 134.3, 133.9, 132.2, 132.1, 131.7, 131.6, 130.0, 129.8, 128.9, 128.9, 128.8, 128.7, 128.6, 128.5, 128.4, 127.8, 127.5, 127.2, 127.2, 127.0, 127.0, 126.7, 126.6, 126.4, 126.0, 125.9, 125.8, 125.7, 125.5, 125.5, 124.9, 124.8, 124.7, 123.2, 122.9, 110.5, 109.0, 70.5, 69.0, 68.8, 66.1, 65.6, 63.7, 58.4, 57.7, 43.2, 42.8, 41.5, 41.5, 29.2, 21.0, 20.7, 20.5; IR(KBr) υ: 3403, 3230, 3028, 2922, 2825, 1713, 1685, 1606, 1496, 1474, 1435, 1342, 1288, 1220, 1180, 1120, 1079, 1008, 942, 810, 743cm^{-1}; MS (*m/z*): HRMS (ESI) Calcd. for $C_{42}H_{35}ClN_3O_3$ ([M+H]$^+$): 664.2361, found: 664.2356.

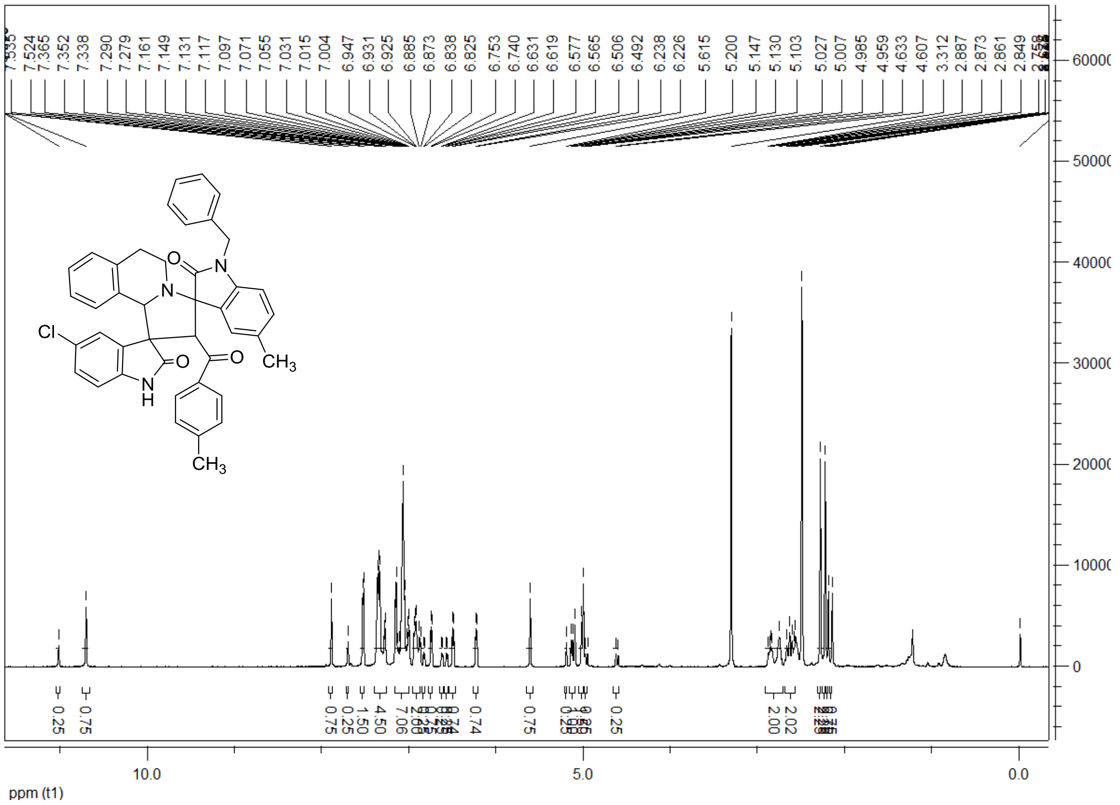

Copy of ^{1}H NMR spectrum of **4u** in DMSO-d_6

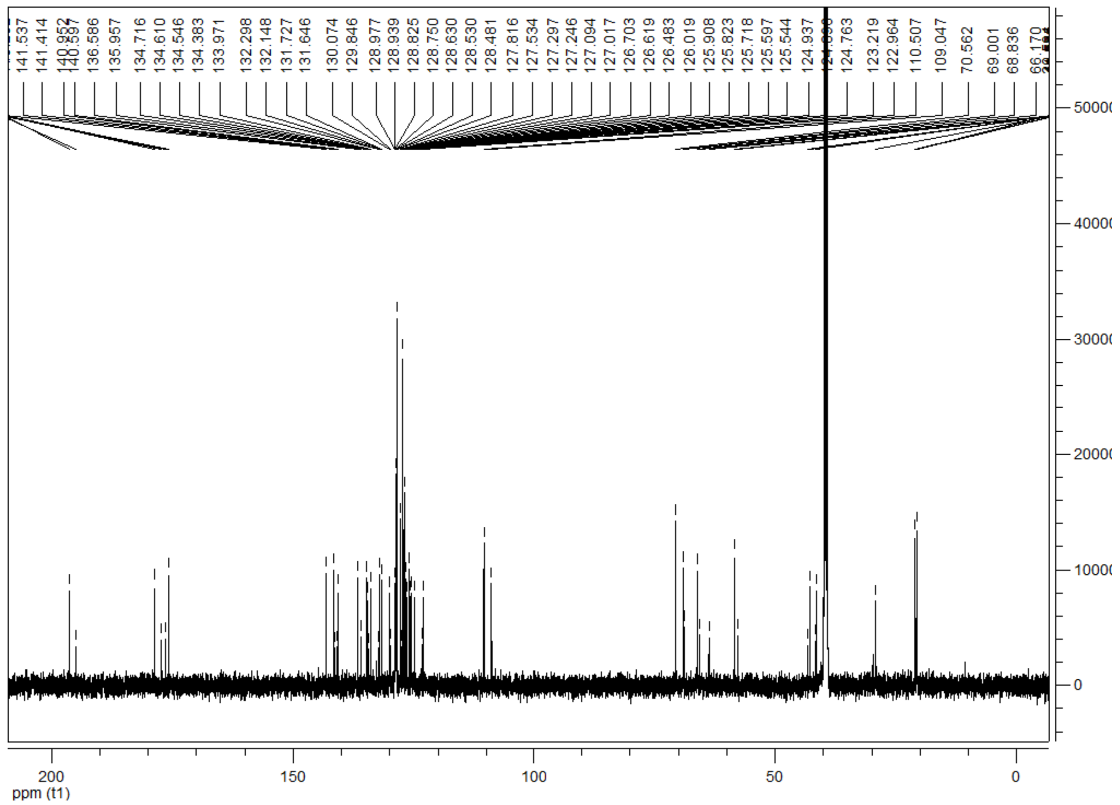

Copy of ^{13}C NMR spectrum of **4u** in DMSO-d_6

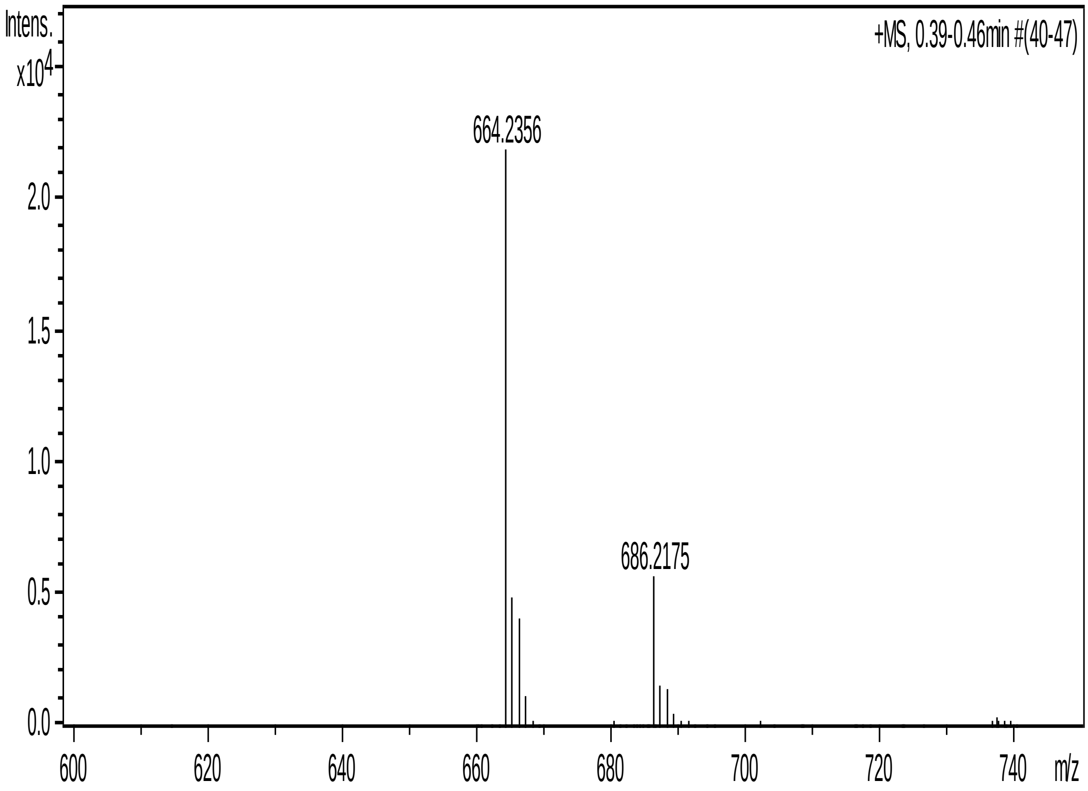

Copy of HRMS of **4u**

1''-Benzyl-5,5''-dichloro-2'-(4-methylbenzoyl)-6',10b'-dihydro-2'H,5'H-dispiro[indoline-3,1'-pyrrolo[2,1-a]isoquinoline-3',3''-indoline]-2,2''-dione (4v)

white solid, 76%, m.p. 242~243℃; ^{1}H NMR (400 MHz, DMSO-d_6) δ: 10.71 (s, 1H, NH), 7.84 (s, 1H, ArH), 7.54 (brs, 3H, ArH), 7.38~7.29 (m, 4H, ArH), 7.19~6.89 (m, 9H, ArH), 6.49 (d, *J* = 8.4Hz, 1H, ArH), 6.23 (d, *J* = 7.2Hz, 1H, ArH), 5.57 (s, 1H, CH), 5.17~5.05 (m, 3H, CH), 2.88~2.56 (m, 4H, CH), 2.24 (s, 3H, CH_3); ^{13}C NMR (100 MHz, DMSO-d_6) δ: 196.8, 178.8, 175.9, 143.6, 143.2, 141.0, 136.6, 134.9, 134.9, 134.3, 132.4, 130.2, 129.4, 129.0, 128.9, 128.5, 127.8, 127.7, 127.6, 127.0, 126.9, 126.3, 126.2, 125.8, 125.1, 123.3, 111.1, 111.0, 70.9, 69.2, 66.8, 58.5, 43.4, 42.0, 29.7, 21.4; IR(KBr) υ: 3391, 3192, 2935, 2843, 1717, 1677, 1609, 1483, 1432, 1336, 1290, 1253, 1218, 1175, 1119, 1077, 1038, 980, 898, 815, 784, 740cm^{-1}; MS (*m/z*): HRMS (ESI) Calcd. for $C_{41}H_{31}N_3NaO_3$ ([M+Na]$^+$): 706.1635, found: 706.1627.

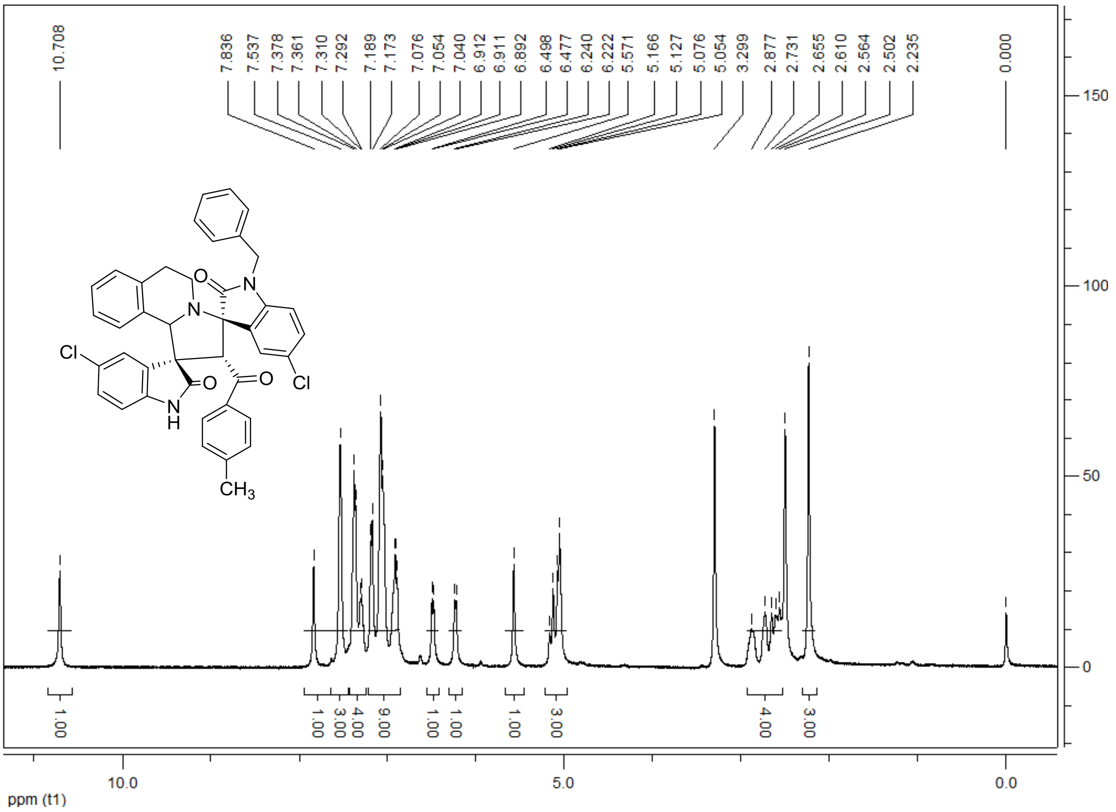

Copy of ^{1}H NMR spectrum of **4v** in DMSO-d_6

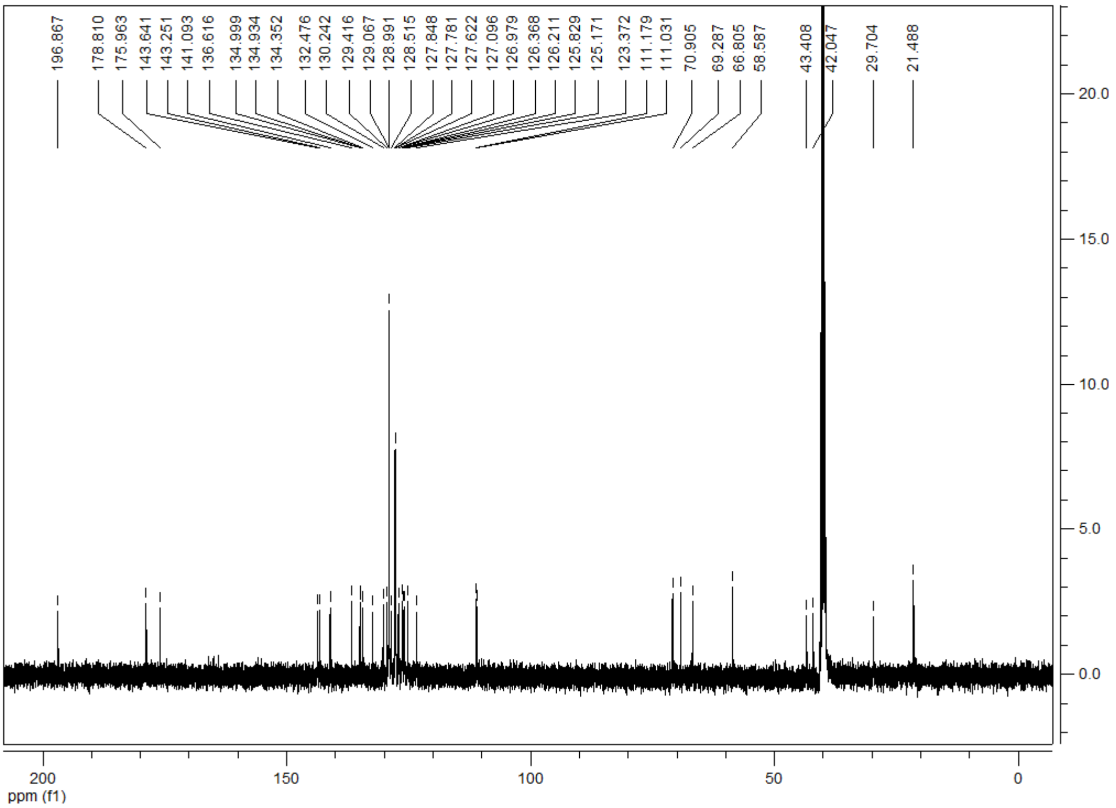

Copy of ^{13}C NMR spectrum of **4v** in DMSO-d_6

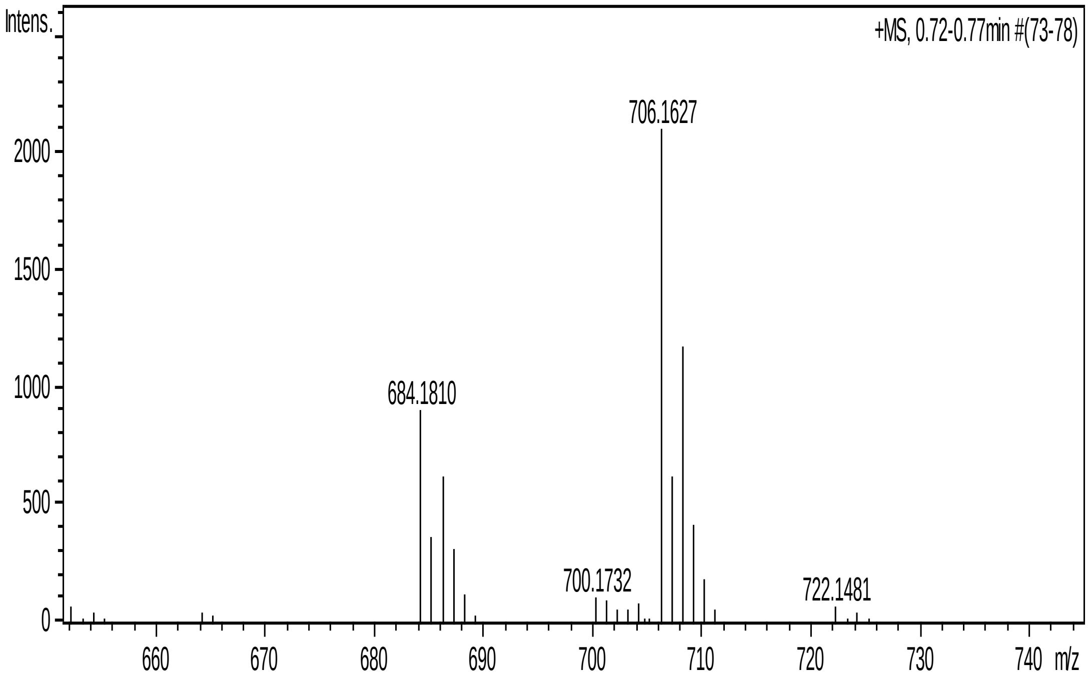

Copy of HRMS of **4v**

1,1''-Dibenzyl-2'-(4-chlorobenzoyl)-5,5''-dimethyl-6',10b'-dihydro-2'H,5'H-dispiro[indoline-3,1'-pyrrolo[2,1-a]isoquinoline-3',3''-indoline]-2,2''-dione (4w)

white solid, 60%, m.p. 150~152℃; ^{1}H NMR (600 MHz, DMSO-d_6) δ: 7.72 (s, 1H, ArH), 7.54~7.52 (m, 2H, ArH), 7.37~7.35 (m, 3H, ArH), 7.31~7.28 (m, 4H, ArH), 7.24 (brs, 2H, ArH), 7.15~7.14 (m, 2H, ArH), 7.09~7.06 (m, 5H, ArH), 6.90 (d, J = 7.2Hz, 1H, ArH), 6.74 (d, J = 7.8Hz, 1H, ArH), 6.65 (m, 1H, ArH), 6.54 (d, J = 7.2Hz, 1H, ArH), 6.13 (d, J = 7.2Hz, 1H, ArH), 5.70 (s, 1H, CH), 5.11 (d, J = 16.2Hz, 1H, CH), 5.02 (d, J = 13.2Hz, 2H, CH), 4.95 (d, J = 15.6Hz, 1H, CH), 4.18 (d, J = 15.6Hz, 1H, CH), 2.89~2.87 (m, 1H, CH), 2.79~2.77 (m, 1H, CH), 2.65~2.59 (m, 2H, CH), 2.30 (s, 3H, CH_3), 2.24 (s, 3H, CH_3); ^{13}C NMR (150 MHz, DMSO-d_6) δ: 196.0, 177.3, 175.4, 141.5, 140.0, 137.4, 136.5, 135.8, 135.2, 134.4, 134.4, 131.8, 131.5, 130.0, 129.1, 128.9, 128.5, 128.4, 128.2, 128.2, 128.0, 127.4, 127.2, 127.2, 126.4, 126.1, 125.9, 125.6, 125.1, 123.3, 108.9, 108.2, 70.3, 68.6, 66.8, 57.5, 44.0, 42.8, 41.5, 29.2, 20.9, 20.6; IR(KBr) υ: 3435, 3028, 2917, 1711, 1593, 1496, 1431, 1345, 1293, 1193, 1165, 1088, 1003, 809, 743cm^{-1}; MS (m/z): HRMS (ESI) Calcd. for $C_{49}H_{41}ClN_3O_3$ ($[M+H]^+$): 754.2831, found: 754.2822.

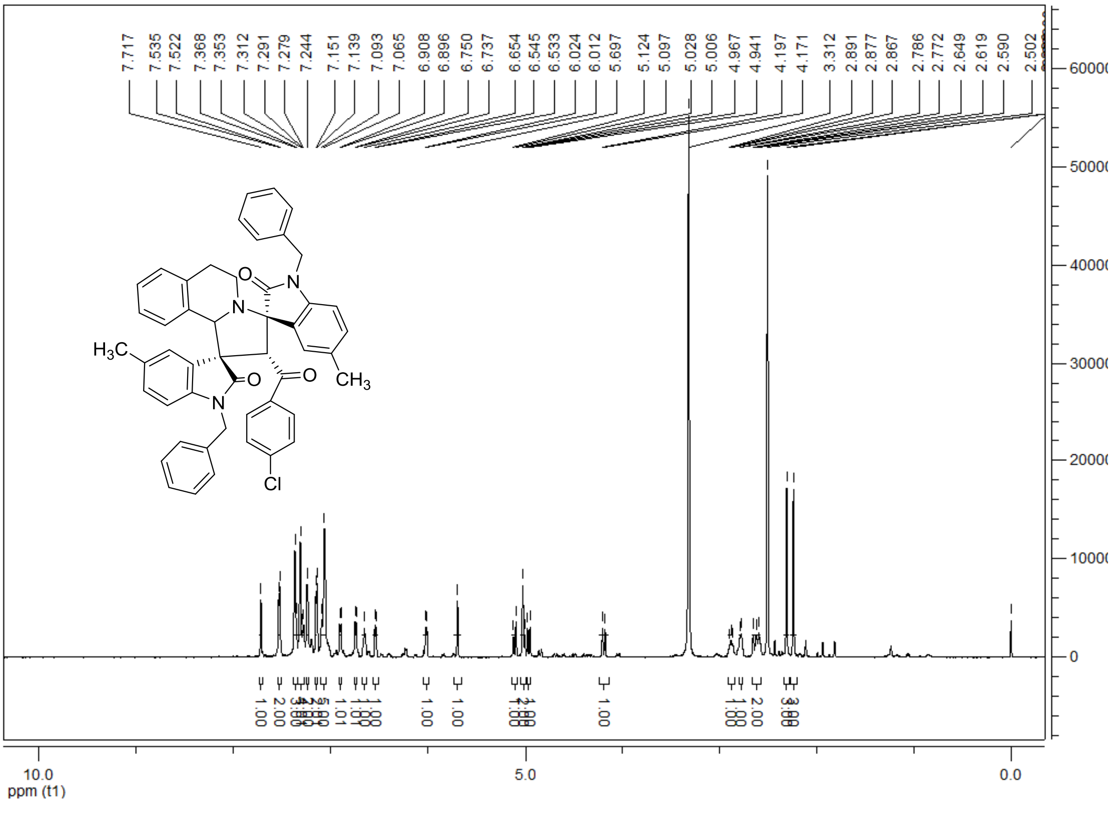

Copy of ^{1}H NMR spectrum of **4w** in DMSO-d_6

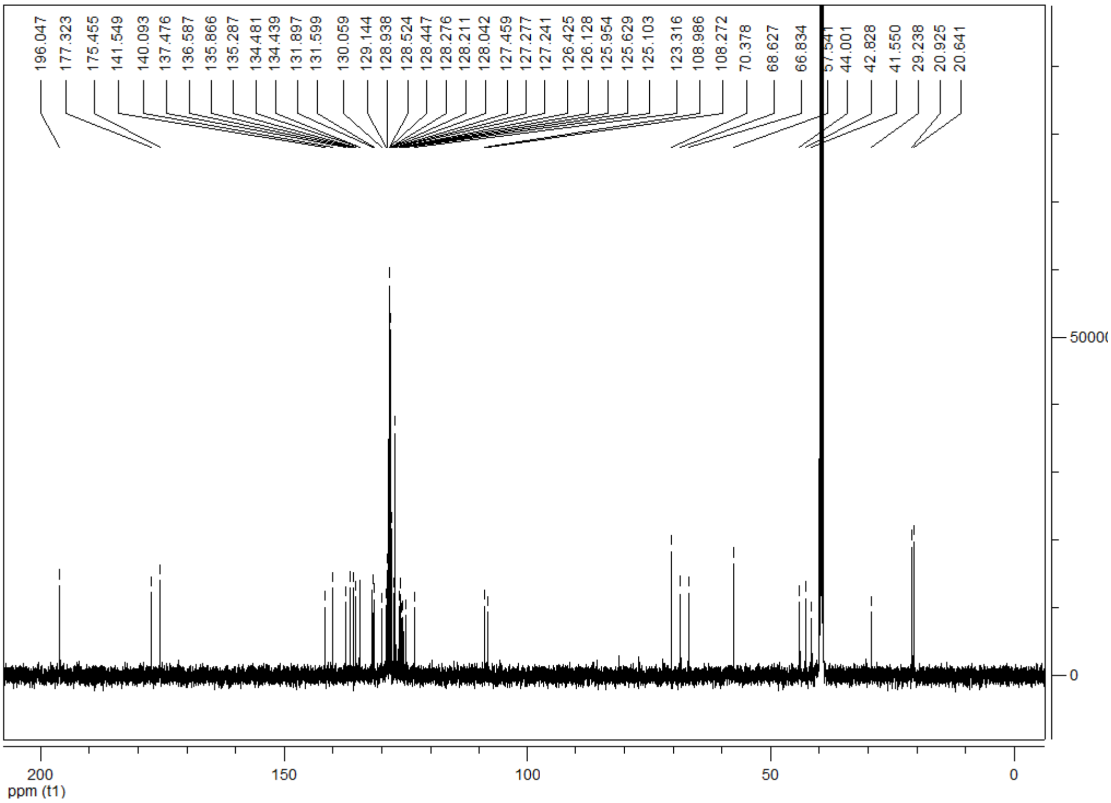

Copy of ^{13}C NMR spectrum of **4w** in DMSO-d_6

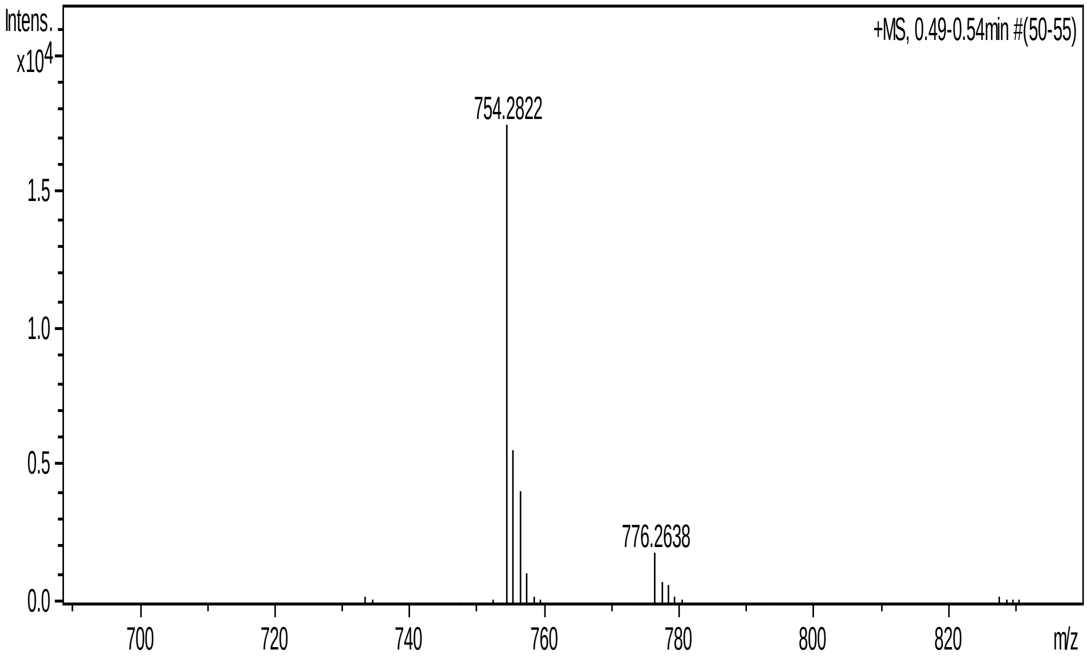

Copy of HRMS of **4w**

1-Butyl-5-chloro-5''-methyl-2'-(4-methylbenzoyl)-6',10b'-dihydro-2'H,5'H-dispiro[indoline-3,1'-pyrrolo[2,1-a]isoquinoline-3',3''-indoline]-2,2''-dione (4x)

white solid, 50%, m.p. 225~226℃; ^{1}H NMR (400 MHz, DMSO-d_6) δ: 10.43 (s, 1H, NH), 7.55 (s, 1H, ArH), 7.45~7.42 (m, 2H, ArH), 7.15 (d, J = 8.4Hz, 1H, ArH), 7.10 (d, J = 8.0Hz, 2H, ArH), 7.06~7.05 (m, 2H, ArH), 6.98 (d, J = 8.0Hz, 2H, ArH), 6.91~6.86 (m, 1H, ArH), 6.81 (d, J = 8.4Hz, 1H, ArH), 6.35 (d, J = 7.6Hz, 1H, ArH), 6.23 (d, J = 7.6Hz, 1H, ArH), 5.54 (s, 1H, CH), 4.92 (s, 1H, CH), 3.92~3.85 (m, 1H, CH), 3.79~3.72 (m, 1H, CH), 2.91~2.85 (m, 1H, CH), 2.68~2.53 (m, 3H, CH), 2.20 (s, 6H, CH_3), 1.74~1.66 (m, 2H, CH), 1.47~1.41 (m, 2H, CH), 0.97 (t, J = 7.2Hz, 3H, CH_3); ^{13}C NMR (150 MHz, DMSO-d_6) δ: 196.2, 178.8, 175.0, 143.2, 142.7, 139.1, 135.0, 134.4, 134.1, 130.5, 130.0, 129.7, 128.8, 128.7, 128.6, 128.4, 127.0, 126.2, 126.1, 125.9, 125.5, 124.2, 123.0, 110.0, 108.7, 70.0, 68.6, 66.5, 57.7, 41.4, 29.2, 28.9, 20.9, 19.5, 13.6; IR(KBr) υ: 3435, 3029, 2929, 2869, 1709, 1608, 1489, 1431, 1341, 1293, 1265, 1184, 1118, 1041, 1010, 949, 814, 749cm^{-1}; MS (m/z): HRMS (ESI) Calcd. for $C_{39}H_{37}ClN_3O_3$ ([M+H]$^+$): 630.2518, found: 630.2513.

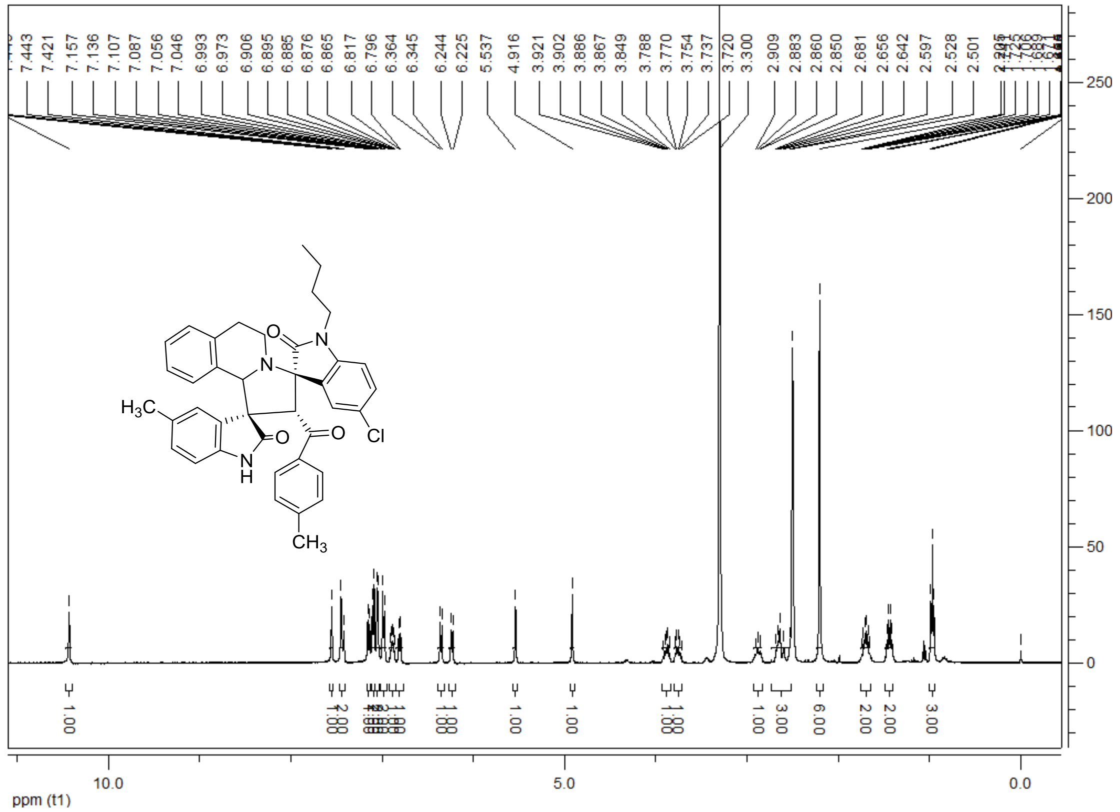

Copy of ^{1}H NMR spectrum of **4x** in DMSO-d_6

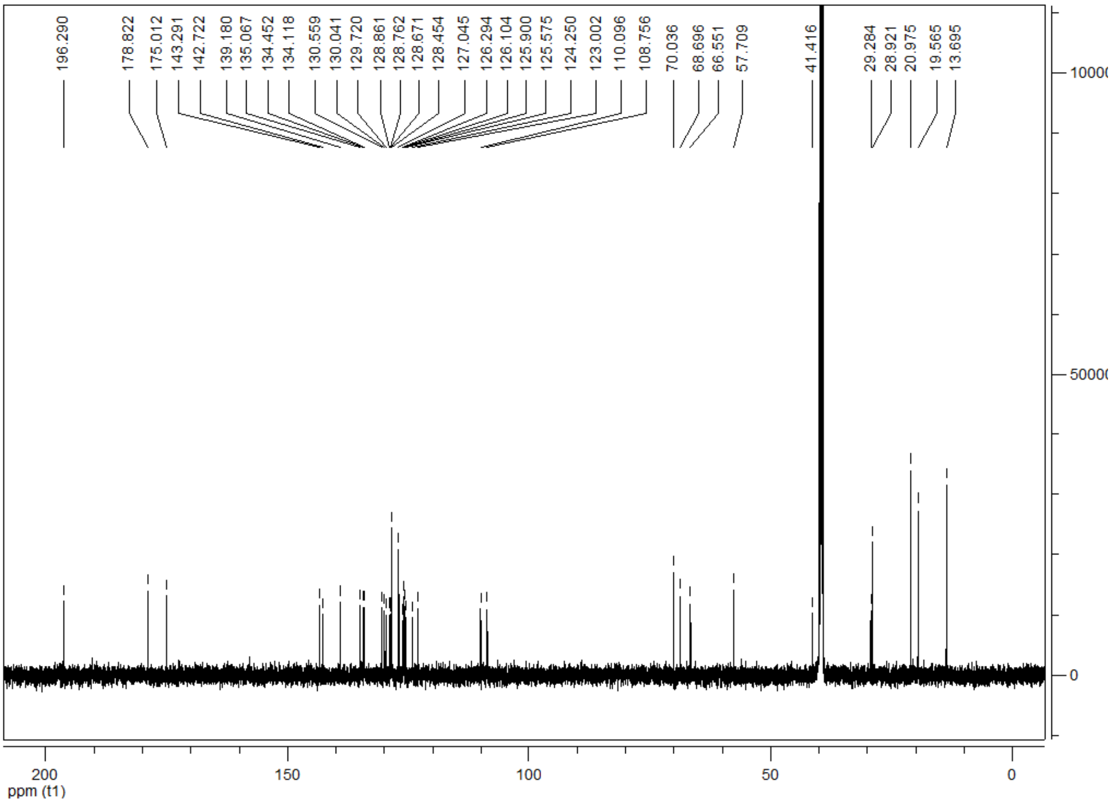

Copy of ^{13}C NMR spectrum of **4x** in DMSO-d_6

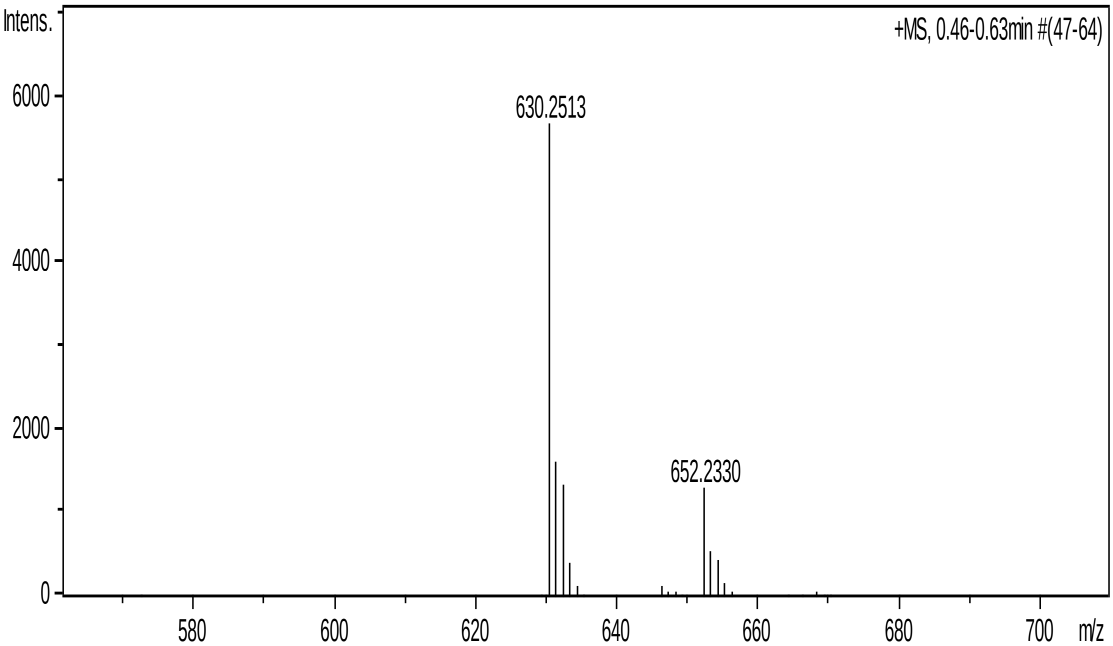

Copy of HRMS of **4x**
